# Synthesis of 4-amino-5-fluoropyrimidines and 5-amino-4-fluoropyrazoles from a β-fluoroenolate salt

**DOI:** 10.3762/bjoc.16.41

**Published:** 2020-03-20

**Authors:** Tobias Lucas, Jule-Philipp Dietz, Till Opatz

**Affiliations:** 1 Institute of Organic Chemistry, Johannes Gutenberg-University, Duesbergweg 10–14, 55128 Mainz, Germany

**Keywords:** cyclization, fluorinated heterocycles, fluorine, pyrazoles, pyrimidines

## Abstract

A synthesis of fluorinated pyrimidines under mild conditions from amidine hydrochlorides and the recently described potassium 2-cyano-2-fluoroethenolate was developed. A broad substrate scope was tested and mostly excellent yields were obtained. The synthesis of fluorinated aminopyrazoles from the same fluorinated precursor could be demonstrated but proceeded with lower efficiency.

## Introduction

Due to its extremely widespread application in virtually all areas of synthetic organic chemistry, fluorine is considered a “magic” element. It confers metabolic stability and other unique properties to organic molecules and a large fraction of modern pharmaceuticals [1–6] and agrochemicals [7] contain at least one fluorine atom [8–15]. One of the approaches for their synthesis is the use of small fluorinated building blocks which avoids the often cumbersome regioselective fluorination at a later synthetic stage [15–19].

**Figure 1:**
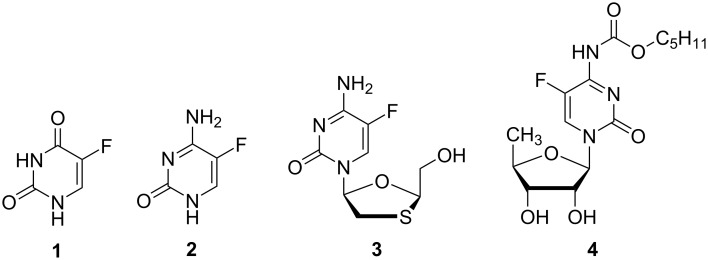
The structures of 5-fluorouracil (**1**), 5-fluorocytosine (**2**), emtricitabine (**3**) and capecitabine (**4**).

Fluorinated pyrimidines, pyrimidine analogues and pyrazoles [20–21] play a prominent role in several clinically important pharmaceuticals [22–25]. These compounds act against HIV or HIV-related diseases [26], such as opportunistic fungal infections, and represent nucleoside analogues which can either act as antimetabolites or as nucleoside reverse transcriptase inhibitors (NRTIs). Two of the most common 5-fluoropyrimidines are the cytostatic 5-fluorouracil (**1**) [27] and the antimycotic prodrug 5-fluorocytosine (**2**) [28–29], which is also part of the anti-HIV agents emtricitabine [30] (ingredient of Truvada^®^) and capecitabine [31] (Xeloda^®^) (see Figure 1) [32–33].

**Scheme 1:**
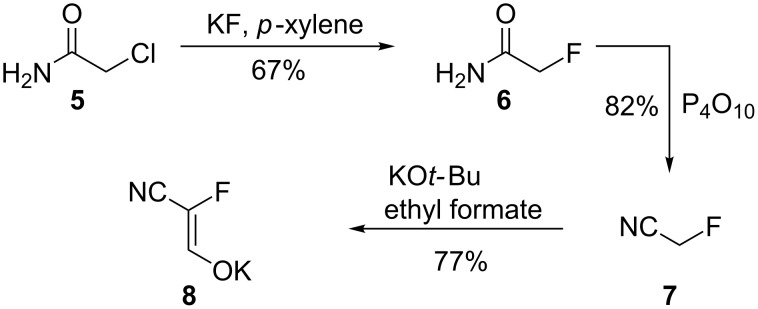
Synthesis of potassium (*Z*)-2-cyano-2-fluoroethenolate (**8**) by Dietz et al. [36].

While the introduction of fluorine into preexisting heterocycles can require difficult-to-handle, expensive and highly corrosive electrophilic fluorine species [16,34–35], the present approach employs a fluorinated C_3_ building block recently described and used by our laboratory for the synthesis of **2** [36]. Herein, we present the synthesis of fluorinated pyrimidines and pyrazoles from the same precursor. Potassium (*Z*)-2-cyano-2-fluoroethenolate (**8**) was synthesized in three steps starting from chloroacetamide (**5**). First, a Finkelstein halogen exchange reaction and a dehydration reaction were combined to obtain fluoroacetonitrile (**7**) in 82% yield [36]. The latter then underwent a Claisen condensation with ethyl formate to obtain **8** in 77% yield with a purity of 90%. The product contained about 10% (^1^H NMR) of potassium formate, which could not easily be removed without risking the decomposition of the enolate salt (Scheme 1).

As the fluorine atom is part of a building block, harsh conditions for a late-stage fluorination can be avoided, and even products with sensitive functionalities are accessible.

## Results and Discussion

### Synthesis of fluorinated pyrimidines

To synthesize 2-alkyl- and 2-aryl-substituted 5-fluoro-4-aminopyrimidines, the reaction of **8** with various amidines was investigated. Different formamidinium and guanidinium salts were tested (Table 1) to evaluate the effect of the counter ion. The hydrochlorides furnished the highest yields and no undesired byproducts were formed. In contrast to the related reaction with guanidinium salts [36], the reactions with amidine hydrochlorides did not require any basic additives.

**Table 1:** Testing of different counter ions.

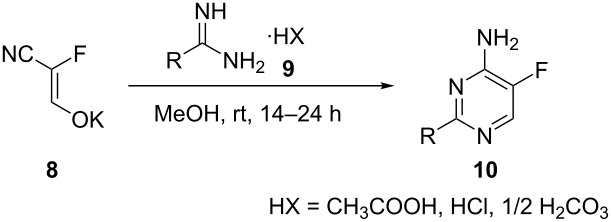				

Entry	Starting material	R	Counter ion	Yield

1	**9a**	H	acetate	78% (**10a**)
2	**9b**	H	chloride	85% (**10a**)
3	**9c**	NH_2_	carbonate	90% (**10b**)
4	**9d**	NH_2_	chloride	94% (1**0b**)

**Scheme 2:**
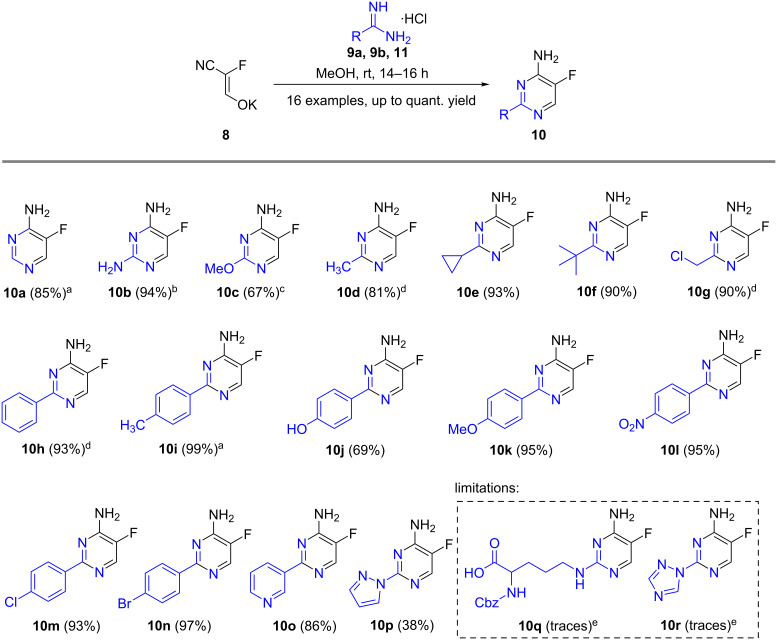
Scope of the cyclization reaction. All yields are those of the purified products. ^a^No further purification was required to obtain analytically pure material. ^b^Use of sodium methoxide. ^c^Using *O*-methylisourea hemisulfate. ^d^Reaction time of 24 h. ^e^Product detected by LC–MS.

Under these improved conditions, the scope of the reaction for producing alkyl-substituted pyrimidine derivatives was investigated (Scheme 2). Starting from formamidine hydrochloride, compound **10a** was synthesized in 85% yield. A variety of amidines with different steric hindrance such as methyl, cyclopropyl and *tert*-butyl substitution was used, and all substituents were well tolerated, providing the corresponding fluorinated aminoalkylpyrimidines (**10d**–**f**) in yields of 81–93%.

In spite of its potential electrophilic reactivity, the chloromethyl derivative **10g** was obtained in excellent yield (90%) and could be used for further modifications to construct more complex products. Subsequently, benzamidine derivatives with a broad variety of substituents on the benzenoid core were evaluated as the nucleophiles. Benzamidine hydrochloride itself yielded compound **10h** with an excellent yield of 93%. A variety of substituted products including electron-donating (compounds **10i**–**k**) and electron-withdrawing groups (compound **10l**) were formed in nearly quantitative yields (95–99%). Only the 4-hydroxyphenyl derivative **10j** was obtained in a moderate yield (69%) presumably due to competing polymerization of the enolate.

The 4-chloro and 4-bromo derivatives (**10m** and **10n**) were also obtained in excellent yields of 93% and 97%, respectively, and represent potential substrates for subsequent cross-coupling reactions, which would provide structurally more complex products.

Amidines with heterocyclic substituents were also evaluated. While the pyridine-substituted derivative **10o** furnished a yield of 86%, the pyrazolo derivative **10p** could only be obtained in a moderate yield of 38%. This might be explained by the tendency of the pyrazolo moiety to act as a leaving group and a following addition–elimination reaction with methanol can take place (the corresponding 2-methoxy-substituted product was detected by LC–MS). This side reaction could also be observed in the attempted synthesis of the triazolo derivative **10r**, where the desired pyrimidine could only be detected in traces by LC–MS. Another limitation of the substrate scope was observed in the cyclization with an *N*_α_-protected arginine, where the desired pyrimidine **10q** was also only observed in trace amounts while the majority of the starting material remained unchanged.

### Synthesis of fluorinated aminopyrazoles

As in the cyclocondensation with amidines, the reaction of **8** with other bifunctional nucleophiles could also result in the formation of fluorinated heterocycles. While attempts to obtain an aminooxazole upon reaction with hydroxylamine were unsuccessful, substituted hydrazines turned out to provide moderate yields of fluorinated pyrazoles. Phenylhydrazine (**12a**) was chosen as the model substrate for optimization (Table 2).

**Table 2:** Synthesis of fluorinated pyrazoles.

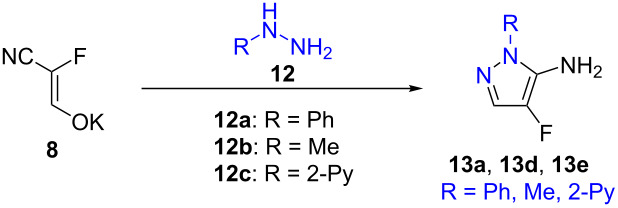					

Entry	R	Solvent	*T* [°C]	Additive	Results

1	Ph	MeOH	rt	–	traces of **13a** and **13b**^a^
2	Ph	MeOH	5–10	–	polymerization
3	Ph	MeOH	−10	–	polymerization
4	Ph^b^	MeOH	−10	–	polymerization
5	Ph	MeOH	5–10	AcOH	polymerization
6	Ph	MeOH	0–5	NaOMe	**13a**–**c**^a^
7	Ph	MeOH	0–5	DBU	polymerization
8	Ph	MeOH	0–5	NaOH_aq_	**13a** (19%)^c^
9	Ph	H_2_O	rt	–	traces of **13a**^a^
10	Ph	H_2_O	rt	AcOH	polymerization
11	Ph^b^	H_2_O	rt	NaOH_aq_	**13a** (36%)^c^
12	Me	H_2_O	rt	–	**13d** (41%)^c^
13	2-Py	H_2_O	rt	–	**13e** (20%)^c^

^a^Detected by LC–MS. ^b^The corresponding hydrochloride was used. ^c^Isolated yield.

**Scheme 3:**
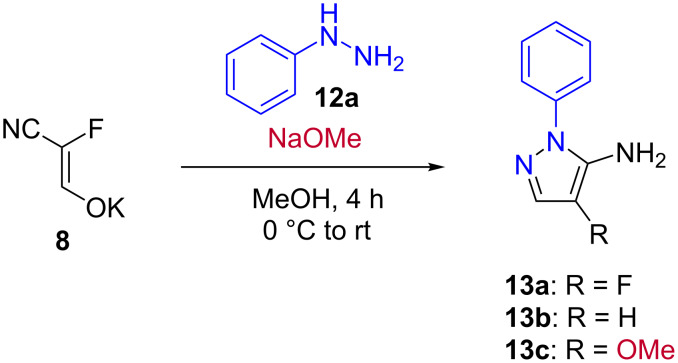
Cyclization with phenylhydrazine (**12a**) to obtain the desired pyrazole **13a** and the byproducts **13b** and **13c** (detected via LC–MS).

In the first reactions, only traces of the desired compound **13a** were observed. Changing the temperature did not increase the yield. Acidic conditions were tested next but phenylhydrazine hydrochloride and acetic acid again gave only traces of **13a** and polymerization of the enolate took place. To stabilize the starting material, basic conditions were tested as well and various bases were used. With sodium methoxide, only traces of compound **13a** could be detected and in addition, the dehalogenated- and methoxy-substituted derivatives (**13b** and **13c**) were observed (Scheme 3). This prompted us to investigate the use of non-nucleophilic bases, but with DBU, polymerization of the fluoroenolate took place.

When using aqueous sodium hydroxide as the base, a 19% yield could be achieved. Switching the solvent to water led to an improvement of the yield to 36% (Table 2). In water, but without using a base, the *N*-methylpyrazole **13d** and the *N*-(2-pyridyl)-substituted derivative **13e** could be obtained in 41% and 21% yield, respectively.

The formation of the other possible aminopyrazole regioisomer was not observed, which could be explained by the preferential attack of the less hindered nitrogen atom of the hydrazine on the more reactive carbon atom of the fluoroenolate in a Michael-type addition. The cyclization did not require a deprotonation of the RNH moiety.

## Conclusion

In summary, a synthesis of fluorinated pyrimidines under mild conditions using fluoroenolate **8** and amidines in a cyclocondensation was developed. A series of substrates carrying aliphatic and aromatic substituents was successfully tested and various functional groups were tolerated. The products could be mostly isolated in excellent yields. Furthermore, the synthesis of fluorinated pyrazoles from the same fluorinated enolate was demonstrated, albeit in only low to moderate yields. Nevertheless, the presented method provides a simple and short route to hitherto unknown compounds of this type.

## Experimental

See Supporting Information File 1 for full experimental data.

## Supporting Information

File 1 Chemical procedures and analytical data, including copies of ^1^H NMR and ^13^C NMR spectra.
