# Supplementary material for: Synthesis of 4-amino-5-fluoropyrimidines and 5-amino-4-fluoropyrazoles from a β-fluoroenolate salt
Source: Beilstein J Org Chem. 2020 Mar 20;16:445–50. doi: 10.3762/bjoc.16.41 (PMC7113542; doi:10.3762/bjoc.16.41)
Supplement: File 1 — Chemical procedures and analytical data, including copies of 1H NMR and 13C NMR spectra. [file Beilstein_J_Org_Chem-16-445-s001.pdf]

BEILSTEIN JOURNAL OF ORGANIC CHEMISTRY

Supporting Information

for

Synthesis of 4-amino-5-fluoropyrimidines and 5-amino-4-fluoropyrazoles from a β-fluoroenolate salt

Tobias Lucas, Jule-Philipp Dietz and Till Opatz

Beilstein J. Org. Chem. **2020,** *16,* 445–450. doi:10.3762/bjoc.16.41

Chemical procedures and analytical data, including copies of ^{1}H NMR and ^{13}C NMR spectra

License and Terms: This is a supporting information file under the terms of the Creative Commons Attribution License (http://creativecommons.org/licenses/by/4.0). Please note that the reuse, redistribution and reproduction in particular requires that the authors and source are credited.
The license is subject to the *Beilstein Journal of Organic Chemistry* terms and conditions: (https://www.beilstein-journals.org/bjoc)

Experimental Section

General Information

Unless stated otherwise, all chemicals were obtained from commercial suppliers and used without further purification. Deuterated solvents were purchased from Deutero GmbH (Kastellaun, Germany). Dry methanol was purchased from Acros Organics. Anhydrous THF was freshly distilled over sodium. The eluents for column chromatography cyclohexane and ethyl acetate were purchased in technical grade and distilled prior to use. All air or moisture sensitive reactions were performed under an inert atmosphere of argon in glassware that was oven dried using standard Schlenk techniques. Reaction temperatures referred to the temperature of the particular cooling or heating bath. Chromatographic purification was performed using flash column chromatography of the indicated solvent system on silica gel (35–70 µm, Acros Organics) unless otherwise noted. Alternatively, purification was performed on an Isolera Flash Purification System (Biotage) with an integrated diode array detector. Thin-layer chromatography (TLC) was carried out on silica plates (TLC Silica 60 F_{254}, *Merck*). UV active compounds were visualized using UV light (λ = 254 nm und λ = 365 nm). All NMR spectra were recorded on the following spectrometers: Bruker Avance-III HD (^{1}H-NMR: 300 MHz, ^{13}C-NMR: 75.5 MHz, ^{19}F-NMR: 282 MHz), Bruker Avance-II (^{1}H-NMR: 400 MHz, ^{13}C-NMR: 100.6 MHz, ^{19}F-NMR: 377 MHz), Bruker Avance-III (^{1}H-NMR: 600 MHz, ^{13}C-NMR: 151.1 MHz, ^{19}F-NMR: 565 MHz). Chemical shifts are referenced to residual solvent signals ([D]chloroform: 7.26 ppm and 77.16 ppm, [D_6]DMSO: 2.50 ppm and 39.52 ppm for ^{1}H-NMR and ^{13}C-NMR respectively) and reported in parts per million (ppm) relative to tetramethylsilane (^{1}H, ^{13}C) and trichlorofluoromethane (^{19}F). Infrared spectra were recorded on a spectrometer (Bruker Tensor 27) equipped with a diamond ATR unit. Electron spray ionization (ESI) mass spectra were recorded on a 1200-series HPLC-system or a 1260-series Infinity II HPLC-system (Agilent-Technologies) with binary pump and integrated diode array detector coupled to a LC/MSD-Trap-XTC-mass spectrometer (Agilent-Technologies) or a LC/MSD Infinitylab LC/MSD (G6125B LC/MSD). High resolution mass spectra were recorded on a 6545 Q-ToF-mass spectrometer with LockSpray-interface. melting points were determined by using a Krüss-Optronic KSP 1 N digital melting point meter.

Procedures and analytical data

5-Fluoropyrimidine-2,4-diamine (10b): The compound was synthesized after a modified procedure by *Dietz et al*. [1]. Potassium-(*Z*)-2-cyano-2-fluoroethenolate (**8**) (188 mg, 1.5 mmol, 1.0 eq, corrected for formate content) was added was added to a suspension of guanidine hydrochloride (287 mg, 3.0 mmol, 2.0 eq) in dry methanol (15 mL) under argon atmosphere. A 5.4 m solution of sodium methoxide (0.6 mL, 2.0 mmol, 2.0 eq) was added dropwise at rt and the mixture was stirred for 16 h. After completion the solvent was removed in vacuo. The residue was taken up in water and ethyl acetate, the phases were separated and the aqueous phase was extracted three times with ethyl acetate. The organic layers were combined, washed with brine, dried over sodium sulfate and all volatiles were removed in vacuo. The crude product was washed with cold water (2 mL) and ethyl acetate (2 mL) and was dried in vacuo. Yield: 181 mg (1.4 mmol, 94%), slight yellow solid, R_f = 0.13 (EtOAc), melting Point: 156.5–160.7 °C. ^{1}H-NMR, COSY (300 MHz, [D_6]DMSO): *δ*/ppm = 7.64 (d, 1H, *J* = 3.9 Hz, H-6),

6.63 (s, 2H, NH_2), 5.80 (s, 2H, NH_2). ^{13}C-NMR, HSQC, HMBC (75 MHz, [D_6]DMSO): δ/ppm = 159.8 (d, J = 2.9 Hz, C-2), 153.6 (d, J = 12.3 Hz, C-4), 140.0 (d, J = 239.4 Hz, C-5), 140.0 (d, J = 18.4 Hz, C-6). ^{19}F-NMR (282 MHz, [D_6]DMSO): δ/ppm = –171.3 (d, J = 3.9 Hz). IR (ATR): $\bar{\nu}$ [cm^{-1}] = 3317, 2989, 2179, 1604, 1433, 1208, 774, 566. MS (ESI): *m/z* (%) = 129.1 (100) $[M+H]^+$. HR-MS (ESI): 129.0570 ($[M+H]^+$, calc.: 129.0571). The analytical data are consistent with those reported in the literature [1].

5-Fluoro-2-methoxypyrimidine-4-amine (10c): The compound was synthesized after a modified procedure by *Dietz et al*. [1]. Potassium-(Z)-2-cyano-2-fluoroethenolate (**8**) (0.766 g, 6.12 mmol, 1.0 eq, corrected for formate content) was added to a suspension of *O*-methylisourea hemisulfate (1.130 g, 9.18 mmol, 1.5 eq) in dry methanol (15 mL) under argon atmosphere. The mixture was stirred for 14 h and the solvent was removed in vacuo. The residue was taken up in water and ethyl acetate, the phases were separated and the aqueous phase was extracted three times with ethyl acetate. The organic layers were combined, washed with brine, dried over sodium sulfate and all volatiles were removed in vacuo. The crude product was purified by column chromatography (^{c}Hex/EtOAc, Isolera Flash Purification System, gradient from 0 to 100% EtOAc). Yield: 0.584 g (4.08 mmol, 67%), colorless solid, R_f = 0.27 (^{c}Hex/EtOAc 1:1). M.p. 190.4–191.3 °C. ^{1}H-NMR, COSY (300 MHz, [D_6]DMSO): δ/ppm = 7.93 (d, 1H, J = 3.5 Hz, H-6), 7.27 (s, 2H, NH_2), 3.74 (s, 3H, CH_3). ^{13}C-NMR, HSQC, HMBC (75 MHz, [D_6]DMSO): δ/ppm = 160.5 (d, J = 1.5 Hz, C-2), 154.9 (d, J = 13.7 Hz, C-4), 142.1 (d, J = 245.3 Hz, C-5), 139.7 (d, J = 20.4 Hz, C-6), 54.1 (s, CH_3). ^{19}F-NMR (282 MHz, [D_6]DMSO): δ/ppm = –165.9 (d, J = 3.5 Hz). IR (ATR): $\bar{\nu}$ [cm^{-1}] = 3361, 3314, 3143, 165, 1456, 1392, 1352, 777. MS (ESI): *m/z* (%) = 144.0 (100) $[M+H]^+$. HR-MS (ESI): 144.0566 ($[M+H]^+$, calc.: 144.0568). The analytical data are consistent with those reported in the literature [1].

General Procedure for the synthesis of pyrimidine-derivatives from amidine hydrochlorides:

The amidine hydrochloride (2.0 eq) and potassium-(*Z*)-2-cyano-2-fluoroethenolate (**8**) were suspended in dry methanol (15 mL) under argon atmosphere. The reaction mixture was stirred for 16 h at room temperature, all volatiles were removed in vacuo and the residue was taken up in water and ethyl acetate. The aqueous phase was extracted three times with ethyl acetate, washed with brine, dried over sodium sulfate and the solvent was removed in vacuo. The crude product was purified by column chromatography (^{c}Hex/EtOAc, Isolera Flash Purification System, gradient from 0 to 100% EtOAc).

5-Fluoropyrimidine-4-amine (10a): The title compound was prepared according to the general procedure from formamidine hydrochloride (242 mg, 3.0 mmol, 2.0 eq) and potassium-(*Z*)-2-cyano-2-fluoroethenolate (**8**) (188 mg, 1.5 mmol, 1.0 eq, corrected for formate content). No further purification was required to obtain analytically pure material. Yield: 141 mg (1.3 mmol, 85%), slight yellow solid, R_f = 0.08 (^{c}Hex/EtOAc 1:1), Melting Point: 181.9–183.3 °C. ^{1}H-NMR, COSY (300 MHz, [D_6]DMSO): δ/ppm = 8.18 (d, 1H, J = 3.2 Hz, H-2), 8.13 (d, 1H, J = 4.0 Hz, H-6), 7.26 (s, 2H, NH_2). ^{13}C-NMR, HSQC, HMBC (75 MHz, [D_6]DMSO): δ/ppm = 153.8 (d, J = 7.7 Hz, C-2), 153.5 (d, J = 10.6 Hz, C-4), 145.1 (d, J = 257.3 Hz, C-5), 139.0 (d, J = 16.7 Hz, C-6). ^{19}F-NMR (282 MHz, [D_6]DMSO): δ/ppm = –153.5 (appt, J = 3.6 Hz). IR (ATR): $\bar{\nu}$ [cm^{-1}] = 3269, 3086, 1671, 1607, 1339, 1218, 916, 602. MS (ESI): *m/z* (%) = 114.1 (100) $[M+H]^+$. HR-MS (ESI): 114.0467 ($[M+H]^+$, calc.: 114.0462). The analytical data are consistent with those reported in the literature [2].

5-Fluoro-2-methylpyrimidine-4-amine (10d): The title compound was prepared according to the general procedure from acetamidine hydrochloride (756 mg, 8.00 mmol, 2.0 eq) and potassium-(*Z*)-2-cyano-2-fluoroethenolate (**8**) (499 mg, 4.00 mmol, 1.0 eq, corrected for formate content). The reaction mixture was stirred for 24 h. Yield: 410 mg (3.23 mmol, 81%), colorless solid, R_f = 0.07 (^{c}Hex/EtOAc 1:1). M.p. 154.3–156.3 °C. ^{1}H-NMR, COSY (300 MHz, [D$_6$]DMSO): *δ*/ppm = 7.99 (d, 1H, *J* = 3.9 Hz, H-6), 7.11 (s, 2H, NH$_2$), 2.30 (d, 3H, *J* = 0.9 Hz, CH$_3$). ^{13}C-NMR, HSQC, HMBC (75 MHz, [D$_6$]DMSO): *δ*/ppm = 162.2 (d, *J* = 7.1 Hz, C-2), 153.1 (d, *J* = 11.0 Hz, C-4), 143.9 (d, *J* = 253.6 Hz, C-5), 138.9 (d, *J* = 16.9 Hz, C-6), 24.7 (d, *J* = 2.6 Hz, CH$_3$). ^{19}F-NMR (282 MHz, [D$_6$]DMSO): *δ*/ppm = –159.9 (d, *J* = 3.9 Hz). IR (ATR): $\tilde{\nu}$ [cm^{-1}] = 3235, 3064, 1667, 1605, 1498, 1448, 1414, 1199. MS (ESI): *m/z* (%) = 128.1 (100) [M+H]$^+$. HR-MS (ESI): 128.0622 ([M+H]$^+$, calc.: 128.0619).

2-Cyclopropyl-5-fluoropyrimidine-4-amine (10e): The title compound was prepared according to the general procedure from Cyclopropanecarboxamidine hydrochloride (965 mg, 8.00 mmol, 2.0 eq) and potassium-(*Z*)-2-cyano-2-fluoroethenolate (**8**) (499 mg, 3.99 mmol, 1.0 eq, corrected for formate content). Yield: 568 mg (3.71 mmol, 93%), colorless solid, R_f = 0.33 (^{c}Hex/EtOAc 1:1). M.p. 127.1–127.9 °C. ^{1}H-NMR, COSY (300 MHz, [D$_6$]DMSO): *δ*/ppm = 7.96 (d, 1H, *J* = 3.9 Hz, H-6), 7.06 (s, 2H, NH$_2$), 1.94–1.83 (m, 1H, CH, cyclopropyl), 0.89–0.79 (m, 4H, 2 x CH$_2$, cyclopropyl). ^{13}C-NMR, HSQC, HMBC (75 MHz, [D$_6$]DMSO): *δ*/ppm = 165.7 (d, *J* = 6.1 Hz, C-2), 153.1 (d, *J* = 11.1 Hz, C-4), 143.8 (d, *J* = 252.2 Hz, C-5), 138.8 (d, *J* = 17.2 Hz, C-6), 17.1 (d, *J* = 2.7 Hz, CH, cyclopropyl), 9.1 (s, CH$_2$, cyclopropyl). ^{19}F-NMR (282 MHz, [D$_6$]DMSO): *δ*/ppm = –160.0 (d, *J* = 3.9 Hz). IR (ATR): $\tilde{\nu}$ [cm^{-1}] = 3317, 3132, 1644, 1594, 1492, 1455, 528, 463. MS (ESI): *m/z* (%) = 154.0(100) [M+H]$^+$. HR-MS (ESI): 154.0774 ([M+H]$^+$, calc.: 154.0775).

2-*tert*-Butyl-5-fluoropyrimidine-4-amine (10f): The title compound was prepared according to the general procedure from pivalamidine hydrochloride (1.093 g, 8.00 mmol, 2.0 eq) and potassium-(*Z*)-2-cyano-2-fluoroethenolate (**8**) (0.500 g, 4.00 mmol, 1.0 eq, corrected for formate content). Yield: 0.605 g (3.58 mmol, 90%), colorless solid, R_f = 0.42 (^{c}Hex/EtOAc 3:1), Melting Point: 72.8–75.5 °C. ^{1}H-NMR, COSY (300 MHz, [D$_6$]DMSO): *δ*/ppm = 8.05 (d, 1H, *J* = 3.7 Hz, H-6), 7.04 (s, 2H, NH$_2$), 1.24 (s, 9H, 3 x CH$_3$). ^{13}C-NMR, HSQC, HMBC (75 MHz, [D$_6$]DMSO): *δ*/ppm = 171.1 (d, *J* = 6.5 Hz, C-2), 152.9 (d, *J* = 10.9 Hz, C-4), 143.7 (d, *J* = 253.9 Hz, C-5), 138.5 (d, *J* = 17.0 Hz, C-6), 38.3 (d, *J* = 1.9 Hz, C$_q$, ^{t}Bu), 29.6 (s, 3 x CH$_3$, ^{t}Bu). ^{19}F-NMR (282 MHz, [D$_6$]DMSO): *δ*/ppm = –159.6 (d, *J* = 3.7 Hz). IR (ATR): $\tilde{\nu}$ [cm^{-1}] = 3497, 3099, 2956, 1645, 1491, 1172, 675, 429. MS (ESI): *m/z* (%) = 170.1 (100) [M+H]$^+$. HR-MS (ESI): 170.1091 ([M+H]$^+$, calc.: 170.1088).

2-(Chloromethyl)-5-fluoropyrimidine-4-amine (10g): The title compound was prepared according to the general procedure from 2-chloroacetamidine hydrochloride (520 mg, 4.0 mmol, 2.0 eq) and potassium-(*Z*)-2-cyano-2-fluoroethenolate (**8**) (252 mg, 2.0 mmol, 1.0 eq, corrected for formate content). The reaction mixture was stirred for 24 h. Yield: 284 mg (1.8 mmol, 90%), colorless solid, R_f = 0.32 (^{c}Hex/EtOAc 1:1). M.p. 142.7–143.9 °C. ^{1}H-NMR, COSY (300 MHz, [D$_6$]DMSO): *δ*/ppm = 8.14 (d, 1H, *J* = 3.8 Hz, H-6), 7.44 (s, 2H, NH$_2$), 4.48 (s, 2H, CH$_2$). ^{13}C-NMR, HSQC, HMBC (75 MHz, [D$_6$]DMSO): *δ*/ppm = 160.1 (d, *J* = 6.7 Hz, C-2), 153.8 (d, *J* = 11.9 Hz, C-4), 144.2 (d, *J* = 256.9 Hz, C-5), 139.2 (d, *J* = 17.9 Hz, C-6), 46.8 (d, *J* = 2.6 Hz, CH$_2$). ^{19}F-NMR (282 MHz,

[D_6]DMSO): δ/ppm = –156.0 (d, *J* = 3.8 Hz). IR (ATR): $\bar{\nu}$ [cm^{-1}] = 3459, 3312, 3179, 2853, 1731, 1483, 1395, 699. MS (ESI): *m/z* (%) = 162.0 (100) $[M+H]^+$. HR-MS (ESI): 162.0233 ($[M+H]^+$, calc.: 162.0229).

5-Fluoro-2-phenylpyrimidine-4-amine (10h): The title compound was prepared according to the general procedure from benzamidine hydrochloride (1.250 g, 7.98 mmol, 2.0 eq) and potassium-(*Z*)-2-cyano-2-fluoroethenolate (**8**) (0.501 g, 4.00 mmol, 1.0 eq, corrected for formate content). The reaction mixture was stirred for 24 h. Yield: 0.702 g (3.71 mmol, 93%), colorless crystals, R_f = 0.59 (^{c}Hex/EtOAc 1:1). M.p. 142.2–144.7 °C. ^{1}H-NMR, COSY (300 MHz, [D_6]DMSO): δ/ppm = 8.25 (d, 1H, *J* = 3.6 Hz, H-6), 8.24–8.19 (m, 2H, H-2',6'), 8.24–8.19 (m, 3H, H-3',4',5'), 7.34 (s, 2H, NH_2). ^{13}C-NMR, HSQC, HMBC (75 MHz, [D_6]DMSO): δ/ppm = 158.6 (d, *J* = 6.5 Hz, C-2), 153.4 (d, *J* = 10.9 Hz, C-4), 144.4 (d, *J* = 253.9 Hz, C-5), 139.5 (d, *J* = 17.0 Hz, C-6), 137.2 (s, C-1'), 129.8 (s, C-4'), 128.2 (s, C-3',5'), 127.4 (s, C-2',6'). ^{19}F-NMR (282 MHz, [D_6]DMSO): δ/ppm = –157.2 (d, *J* = 3.6 Hz). IR (ATR): $\bar{\nu}$ [cm^{-1}] = 3458, 3310, 3171, 2901, 1639, 1483, 1396, 700. MS (ESI): *m/z* (%) = 190.0 (100) $[M+H]^+$. HR-MS (ESI): 190.0777 ($[M+H]^+$, calc.: 190.0775). The analytical data are not consistent with those reported in the literature [3].

5-Fluoro-2-(4-methylphenyl)pyrimidine-4-amine (10i): The title compound was prepared according to the general procedure from 4-methylbenzamidine hydrochloride (512 mg, 3.00 mmol, 2.0 eq) and potassium-(*Z*)-2-cyano-2-fluoroethenolate (**8**) (188 mg, 1.50 mmol, 1.0 eq, corrected for formate content). No further purification was required to obtain analytically pure material. Yield: 301 mg (1.48 mmol, 99%), colorless solid, R_f = 0.35 (^{c}Hex/EtOAc 3:1). M.p. 161.8–164.1 °C. ^{1}H-NMR, COSY (300 MHz, [D_6]DMSO): δ/ppm = 8.22 (d, 1H, *J* = 3.7 Hz, H-6), 8.14–8.09 (m, 2H, H-2',6'), 7.29 (s, 2H, NH_2), 8.27–8.22 (m, 2H, H-3',5'), 2.34 (s, 3H, CH_3). ^{13}C-NMR, HSQC, HMBC (75 MHz, [D_6]DMSO): δ/ppm = 158.7 (d, *J* = 6.2 Hz, C-2), 153.4 (d, *J* = 11.4 Hz, C-4), 144.3 (d, *J* = 255.9 Hz, C-5), 139.4 (d, *J* = 18.0 Hz, C-6), 139.4 (s, C-4'), 134.6 (s, C-1'), 128.8 (s,C-3',5'), 127.4 (s, C-2',6'), 20.8 (s, CH_3). ^{19}F-NMR (282 MHz, [D_6]DMSO): δ/ppm = –157.6 (d, *J* = 3.7 Hz). IR (ATR): $\bar{\nu}$ [cm^{-1}] = 3491, 3306, 3147, 3032, 1643, 1491, 1393, 772. MS (ESI): *m/z* (%) = 204.1 (100) $[M+H]^+$. HR--MS (ESI): 204.0930 ($[M+H]^+$, calc.: 240.0932).

4-(4-Amino-5-fluoropyrimidine-2-yl)phenol (10j): The title compound was prepared according to the general procedure from 4-hydroxybenzamidine hydrochloride (518 mg, 3.00 mmol, 2.0 eq) and potassium-(*Z*)-2-cyano-2-fluoroethenolate (**8**) (187 mg, 1.49 mmol, 1.0 eq, corrected for formate content). Yield: 211 mg (1.03 mmol, 69%), colorless solid, R_f = 0.38 (^{c}Hex/EtOAc 3:1). M.p. > 198.7 °C (decomposition). ^{1}H-NMR, COSY (300 MHz, [D_6]DMSO): δ/ppm = 9.77 (s, 1H, OH), 8.16 (d, 1H, *J* = 3.7 Hz, H-6), 8.09–8.02 (m, 2H, H-2',6'), 7.19 (s, 2H, NH_2), 6.83–6.76 (m, 2H, H-3',5'). ^{13}C-NMR, HSQC, HMBC (75 MHz, [D_6]DMSO): δ/ppm = 159.1 (s, C-4'), 158.8 (d, *J* = 6.1 Hz, C-2), 153.2 (d, *J* = 11.3 Hz, C-4), 144.0 (d, *J* = 254.3 Hz, C-5), 139.3 (d, *J* = 18.0 Hz, C-6), 129.1 (s, H-2',6'), 128.3 (s, C-1'), 114.9 (s, C-3',5'). ^{19}F-NMR (282 MHz, [D_6]DMSO): δ/ppm = –158.9 (d, *J* = 3.7 Hz). IR (ATR): $\bar{\nu}$ [cm^{-1}] = 3454, 3342, 1689, 1601, 1490, 1405, 1246, 781. MS (ESI): *m/z* (%) = 206.0 (100) $[M+H]^+$. HR-MS (ESI): 206.0725 ($[M+H]^+$, calc.: 206.0724).

5-Fluoro-2-(4-methoxyphenyl)pyrimidine-4-amine (10k): The title compound was prepared according to the general procedure from 4-methoxybenzamidine hydrochloride (560 mg, 3.00 mmol,

2.0 eq) and potassium-(*Z*)-2-cyano-2-fluoroethenolate (**8**) (189 mg, 1.51 mmol, 1.0 eq, corrected for formate content). Yield: 314 mg (1.43 mmol, 95%), colorless crystals, R_f = 0.23 (^{c}Hex/EtOAc 3:1). M.p. 146.2–147.1 °C. ^{1}H-NMR, COSY (300 MHz, [D$_6$]DMSO): *δ*/ppm = 8.20 (d, 1H, *J* = 3.7 Hz, H-6), 8.19–8.14 (m, 2H, H-2',6'), 7.26 (s, 2H, NH_2), 7.02–6.96 (m, 2H, H-3',5'), 3.80 (s, 3H, CH_3). ^{13}C-NMR, HSQC, HMBC (75 MHz, [D$_6$]DMSO): *δ*/ppm = 160.7 (s, C-4'), 158.5 (d, *J* = 6.2 Hz, C-2), 153.3 (d, *J* = 11.5 Hz, C-4), 144.1 (d, *J* = 255.0 Hz, C-5), 139.4 (d, *J* = 17.7 Hz, C-6), 129.8 (s, C-1'), 129.0 (s, C-2',6'), 113.5 (s, C-3',5'), 55.1 (s, CH_3). ^{19}F-NMR (282 MHz, [D$_6$]DMSO): *δ*/ppm = –158.4 (d, *J* = 3.7 Hz). IR (ATR): $\tilde{\nu}$ [cm^{-1}] = 3421, 3320, 3165, 3083, 1650, 1516, 1391, 784. MS (ESI): *m/z* (%) = 220.1 (100) [M+H]$^+$. HR-MS (ESI): 220.0878 ([M+H]$^+$, calc.: 220.0881).

5-Fluoro-2-(4-nitrophenyl)pyrimidine-4-amine (10l): The title compound was prepared according to the general procedure from 4-nitrobenzamidine hydrochloride (605 mg, 3.00 mmol, 2.0 eq) and potassium-(*Z*)-2-cyano-2-fluoroethenolate (**8**) (187 mg, 1.49 mmol, 1.0 eq, corrected for formate content). Yield: 333 mg (1.42 mmol, 95%), slight yellow solid, R_f = 0.28 (^{c}Hex/EtOAc 3:1). M.p. 277.6–280.9 °C. ^{1}H-NMR, COSY (300 MHz, [D$_6$]DMSO): *δ*/ppm = 8.47–8.42 (m, 2H, H-2',6'), 8.35–8.30 (m, 3H, H-3',5', H-6), 7.54 (s, 2H, NH_2). ^{13}C-NMR, HSQC, HMBC (75 MHz, [D$_6$]DMSO): *δ*/ppm = 156.6 (d, *J* = 6.1 Hz, C-2), 153.7 (d, *J* = 12.0 Hz, C-4), 148.2 (s, C-4'), 144.8 (d, *J* = 258.5 Hz, C-5), 143.1 (s, C-1'), 139.6 (d, *J* = 18.4 Hz, C-6), 128.5 (s,C-2',6'), 123.6 (s, C-3',5'). ^{19}F-NMR (282 MHz, [D$_6$]DMSO): *δ*/ppm = –155.1 (d, *J* = 3.6 Hz). IR (ATR): $\tilde{\nu}$ [cm^{-1}] = 3425, 3324, 3167, 3030, 1665, 1596, 1343, 707. MS (ESI): *m/z* (%) = 235.0 (100) [M+H]$^+$. HR-MS (ESI): 235.0624 ([M+H]$^+$, calc.: 235.0626).

2-(4-Chlorophenyl)-5-fluoropyrimidine-4-amine (10m): The title compound was prepared according to the general procedure from 4-chlorobenzamidine hydrochloride (1.530 g, 8.0 mmol, 2.0 eq) and potassium-(*Z*)-2-cyano-2-fluoroethenolate (**8**) (0.502 g, 4.0 mmol, 1.0 eq, corrected for formate content). Yield: 0.823 g (3.7 mmol, 93%), colorless crystals, R_f = 0.38 (^{c}Hex/EtOAc 3:1). M.p. 187.1–188.2 °C. ^{1}H-NMR, COSY (300 MHz, [D$_6$]DMSO): *δ*/ppm = 8.26 (d, 1H, *J* = 3.6 Hz, H-6), 8.24–8.19 (m, 2H, H-2',6'), 7.55–7.49 (m, 2H, H-3',5'), 7.39 (s, 2H, NH_2). ^{13}C-NMR, HSQC, HMBC (75 MHz, [D$_6$]DMSO): *δ*/ppm = 157.6 (d, *J* = 6.4 Hz, C-2), 153.5 (d, *J* = 11.9 Hz, C-4), 144.5 (d, *J* = 256.7 Hz, C-5), 139.5 (d, *J* = 18.2 Hz, C-6), 136.1 (s, C-1'), 134.7 (s, C-4'), 129.1 (s,C-2',6'), 128.3 (s, C-3',5'). ^{19}F-NMR (282 MHz, [D$_6$]DMSO): *δ*/ppm = –156.7 (d, *J* = 3.6 Hz). IR (ATR): $\tilde{\nu}$ [cm^{-1}] = 3452, 3298, 3115, 1645, 1596, 1487, 1086, 774. MS (ESI): *m/z* (%) = 224.1 (100) [M+H]$^+$, 226.0 (32) [M+H]$^+$. HR-MS (ESI): 224.0388 ([M+H]$^+$, calc.: 224.0385).

2-(4-Bromophenyl)-5-fluoropyrimidine-4-amine (10n): The title compound was prepared according to the general procedure from 4-bromobenzamidine hydrochloride (702 mg, 2.98 mmol, 2.0 eq) and potassium-(*Z*)-2-cyano-2-fluoroethenolate (**8**) (188 mg, 1.50 mmol, 1.0 eq, corrected for formate content). Yield: 388 mg (1.45 mmol, 97%), colorless crystals, R_f = 0.37 (^{c}Hex/EtOAc 3:1). M.p. 186.9–188.1 °C. ^{1}H-NMR, COSY (300 MHz, [D$_6$]DMSO): *δ*/ppm = 8.25 (d, 1H, *J* = 3.6 Hz, H-6), 8.19–8.12 (m, 2H, H-2',6'), 7.69–7.62 (m, 2H, 2H-3',5'), 7.40 (s, 2H, NH_2). ^{13}C-NMR, HSQC, HMBC (75 MHz, [D$_6$]DMSO): *δ*/ppm = 157.7 (d, *J* = 6.5 Hz, C-2), 153.5 (d, *J* = 10.9 Hz, C-4), 144.5 (d, *J* = 253.9 Hz, C-5), 139.5 (d, *J* = 17.0 Hz, C-6), 136.5 (s, C-1'), 131.3 (s, C-3',5'), 129.4 (s, C-2',6'), 123.6 (s, C-4'). ^{19}F-NMR (282 MHz, [D$_6$]DMSO): *δ*/ppm = –156.6 (d, *J* = 3.6 Hz). IR (ATR): $\tilde{\nu}$ [cm^{-1}] = 3457, 3306, 3026,

1646, 1485, 1405, 771, 406. MS (ESI): *m/z* (%) = 268.2 (98) $[M+H]^+$, 269.9 (100) $[M+H]^+$. HR-MS (ESI): 267.9883 ($[M+H]^+$, calc.: 267.9880).

5-Fluoro-2-(pyridine-3-yl)pyrimidine-4-amine (10o): The title compound was prepared according to the general procedure from pyridine-3-carboxamidine hydrochloride (473 mg, 3.00 mmol, 2.0 eq) and potassium-(*Z*)-2-cyano-2-fluoroethenolate (**8**) (188 mg, 1.50 mmol, 1.0 eq, corrected for formate content). Yield: 245 mg (1.29 mmol, 86%), colorless solid, R_f = 0.26 (EtOAc). M.p. 186.8–188.2 °C. ^{1}H-NMR, COSY (300 MHz, [D$_6$]DMSO): *δ*/ppm = 9.35 (dd, 1H, *J* = 2.2 Hz, *J* = 0.9 Hz, H-2‘, pyridine), 8.64 (dd, 1H, *J* = 4.8 Hz, *J* = 1.7 Hz, H-6‘, pyridine), 8.48 (d app-t, 1H, *J* = 8.0 Hz, *J* = 2.0 Hz, H-4‘, pyridine), 8.29 (d, 1H, *J* = 3.7 Hz, H-6), 7.49 (ddd, 1H, *J* = 8.1 Hz, *J* = 4.8 Hz, *J* = 0.9 Hz, H-5‘, pyridine), 7.47 (s, 2H, NH_2). ^{13}C-NMR, HSQC, HMBC (75 MHz, [D$_6$]DMSO): *δ*/ppm = 157.0 (d, *J* = 6.4 Hz, C-2), 153.7 (d, *J* = 11.8 Hz, C-4), 150.5 (s, C-6‘, pyridine), 148.6 (s, C-2‘, pyridine), 144.6 (d, *J* = 257.1 Hz, C-5), 139.5 (d, *J* = 18.2 Hz, C-6), 134.6 (s, C-4‘, pyridine), 132.6 (s, C-3‘, pyridine), 123.4 (s, C-5‘, pyridine). ^{19}F-NMR (282 MHz, [D$_6$]DMSO): *δ*/ppm = –156.0 (d, *J* = 3.7 Hz). IR (ATR): $\tilde{\nu}$ [cm^{-1}] = 3312, 3093, 2988, 2902, 1657, 1497, 1407, 527. MS (ESI): *m/z* (%) = 191.1 (100) $[M+H]^+$. HR-MS (ESI): 191.0728 ($[M+H]^+$, calc.: 191.0728).

5-Fluoro-2-(1*H*-pyrazolo-1-yl)pyrimidine-4-amine (10p): The title compound was prepared according to the general procedure from 1-amidinopyrazole hydrochloride (1.173 g, 8.00 mmol, 2.0 eq) and potassium-(*Z*)-2-cyano-2-fluoroethenolate (**8**) (0.501 g, 4.00 mmol, 1.0 eq, corrected for formate content). Yield: 0.273 g (1.52 mmol, 38%), colorless solid, R_f = 0.07 (^{c}Hex/EtOAc 1:1). M.p. 53.1–55.4 °C. ^{1}H-NMR, COSY (300 MHz, [D$_6$]DMSO): *δ*/ppm = 8.43 (dd, 1H, *J* = 2.6 Hz, *J* = 0.8 Hz, H-5‘, pyrazole), 8.19 (d, 1H, *J* = 3.3 Hz, H-6), 7.73 (dd, 1H, *J* = 1.6 Hz, *J* = 0.8 Hz, H-3‘, pyrazole), 7.71 (s, 2H, NH_2), 6.49 (dd, 1H, *J* = 2.6 Hz, *J* = 1.6 Hz, H-4‘, pyrazole). ^{13}C-NMR, HSQC, HMBC (75 MHz, [D$_6$]DMSO): *δ*/ppm = 154.6 (d, *J* = 13.4 Hz, C-4), 150.7 (d, *J* = 3.3 Hz, C-2), 143.3 (d, *J* = 252.2 Hz, C-5), 142.0 (s, C-3‘, pyrazole), 139.7 (d, *J* = 20.3 Hz, C-6), 128.9 (s, C-5‘, pyrazole), 107.8 (s, C-4‘, pyrazole). ^{19}F-NMR (282 MHz, [D$_6$]DMSO): *δ*/ppm = –158.9 (d, *J* = 3.3 Hz). IR (ATR): $\tilde{\nu}$ [cm^{-1}] = 3495, 3281, 3158, 2925, 1641, 1444, 756, 461. MS (ESI): *m/z* (%) = 180.1 (100) $[M+H]^+$. HR-MS (ESI): 202.0498 ($[M+Na]^+$, calc.: 202.0499).

4-Fluoro-1-phenyl-1*H*-pyrazole-5-amine (13a): The compound was synthesized after a modified procedure by *Sturm* and *Armbrust* [4]. Phenylhydrazine hydrochloride (255 mg, 1.8 mmol, 1.1 eq) was slowly added to a stirred solution of potassium-(*Z*)-2-cyano-2-fluoroethenolate (**8**) (200 mg, 1.6 mmol, 1.0 eq, corrected for formate content) in water (15 mL) and was stirred for 1 h at room temperature. A solution of sodium hydroxide (154 mg, 3.8 mmol, 2.4 eq) in water (4 mL) was added and the suspension was stirred for 2 h. The aqueous phase was extracted three times with dichloromethane. The organic layers were combined, washed with brine, dried over sodium sulfate and all volatiles were removed in vacuo. The crude product was purified by column chromatography (^{c}Hex/EtOAc, Isolera Flash Purification System, gradient from 0 to 100% EtOAc). Yield: 101 mg (0.6 mmol, 36%), red solid, R_f = 0.17 (^{c}Hex/EtOAc 5:1). M.p. 58.4–60.8 °C. ^{1}H-NMR, COSY (600 MHz, [D$_6$]DMSO): *δ*/ppm = 7.61–7.58 (m, 2H, H-2’,6’), 7.51 (d, 1H, *J* = 4.5 Hz, H-3), 7.50–7.47 (m, 2H, H-3’,5’), 7.36–7.33 (m, 1H, H-4’), 5.26 (s, 2H, NH_2). ^{13}C-NMR, HSQC, HMBC (151 MHz, [D$_6$]DMSO): δ/ppm = 139.2 (s, C-1’), 135.5 (d, *J* = 234.1 Hz, C-4), 132.6 (d, *J* = 24.4 Hz, C-5), 129.0 (s, C-3’,5'), 127.0 (d, *J* = 9.8 Hz, C-3), 126.6 (s,

C-4'), 122.6 (s, C-2',6'). ^{19}F-NMR (282 MHz, [D$_6$]DMSO): *δ*/ppm = –187.3 (d, *J* = 4.5 Hz). IR (ATR): $\bar{\nu}$ [cm^{-1}] = 3400, 3153, 3061, 2923, 2853, 1953, 1496, 748. MS (ESI): *m/z* (%) = 178.0 (100) [M+H]$^+$. HR-MS (ESI): 178.0776 ([M+H]$^+$, calc.: 178.0775).

4-Fluoro-1-methyl-1*H*-pyrazole-5-amine (13d): The compound was synthesized after a modified procedure by *Sturm* and *Armbrust* [4]. Methylhydrazine (47 mg, 1.0 mmol, 1.0 eq) was slowly added to a stirred solution of potassium-(*Z*)-2-cyano-2-fluoroethenolate (**8**) (125 mg, 1.0 mmol, 1.0 eq, corrected for formate content) in water (10 mL) and was stirred for 3 h at room temperature. The aqueous phase was extracted three times with dichloromethane. The organic layers were combined, washed with brine, dried over sodium sulfate and all volatiles were removed in vacuo. The crude product was purified by column chromatography (^{c}Hex/EtOAc, Isolera Flash Purification System, gradient from 0 to 100% EtOAc). Yield: 47 mg (0.4 mmol, 41%), slightly red oil, R_f = 0.08 (^{c}Hex/EtOAc 2:1). ^1H-NMR, COSY (600 MHz, [D$_6$]DMSO): *δ*/ppm = 7.12 (d, *J* = 4.5 Hz, 1H, H-3), 5.06 (s, 2H, NH_2), 3.49 (d, *J* = 0.6 Hz, 3H, CH_3). ^{13}C-NMR, HSQC, HMBC (151 MHz, [D$_6$]DMSO): δ/ppm = 134.4 (d, *J* = 231.4 Hz, C-4), 132.2 (d, *J* = 23.8 Hz, C-3), 123.9 (d, *J* = 9.7 Hz, C-5), 35.1 (s, CH_3). ^{19}F-NMR (282 MHz, [D$_6$]DMSO): *δ*/ppm = –189.2 (d, *J* = 4.5 Hz). IR (ATR): $\bar{\nu}$ [cm^{-1}] = 3320, 3198, 2922, 2853, 1634, 1546, 1444, 1366, 1235. MS (ESI): *m/z* (%) = 116.1 (100) [M+H]$^+$. HR-MS (ESI): 116.0619 ([M+H]$^+$, calc.: 116.0616).

4-Fluoro-1-(pyridine-2-yl)-1H-pyrazole-5-amine (13e): The compound was synthesized after a modified procedure by *Sturm* and *Armbrust* [4]. 2-hydrazinopyridine (109 mg, 1.0 mmol, 1.0 eq) was slowly added to a stirred solution of potassium-(*Z*)-2-cyano-2-fluoroethenolate (**8**) (148 mg, 1.0 mmol, 1.0 eq, corrected for formate content) in water (10 mL) and was stirred for 3 h at room temperature. The aqueous phase was extracted three times with dichloromethane. The organic layers were combined, washed with brine, dried over sodium sulfate and all volatiles were removed in vacuo. The crude product was purified by column chromatography (^{c}Hex/EtOAc, Isolera Flash Purification System, gradient from 0 to 100% EtOAc). Yield: 37 mg (0.2 mmol, 21%), slightly yellow oil, R_f = 0.08 (^{c}Hex/EtOAc 2:1). ^1H-NMR, COSY (300 MHz, [D$_6$]DMSO): *δ*/ppm = 8.42 (ddd, *J* = 5.0, 1.9, 0.9 Hz, 1H, H-6'), 7.96 (ddd, *J* = 8.4, 7.3, 1.9 Hz, 1H, H-4'), 7.84 (d-pseudo-t, *J* = 8.4, 1.0 Hz, 1H, H-3'), 7.59 (d, *J* = 4.1 Hz, 1H, H-3), 7.29 (ddd, *J* = 7.3, 5.0, 1.1 Hz, 1H, H-5'), 6.64 (s, 2H, NH_2). ^{13}C-NMR, HSQC, HMBC (75 MHz, [D$_6$]DMSO): δ/ppm = 154.1 (s, C-2'),147.0 (s, C-6'), 139.4 (s, C-4'), 135.0 (d, *J* = 24.1 Hz, C-3), 133.5 (d, *J* = 233.3 Hz, C-4), 128.9 (d, *J* = 10.2 Hz, C-5), 120.6 (s, C-5'), 112.5 (s, C-3'). ^{19}F-NMR (282 MHz, [D$_6$]DMSO): *δ*/ppm = –189.9 (d, *J* = 4.1 Hz). IR (ATR): $\bar{\nu}$ [cm^{-1}] = 3375, 3279, 3065, 2930 1639, 1520, 1481, 1451, 1410, 1240. MS (ESI): *m/z* (%) = 179.0 (100) [M+H]$^+$. HR-MS (ESI): 179.0726 ([M+H]$^+$, calc.: 179.0728).

References

[1] Dietz, J.-P.; Derstine, B. P.; Ferenc, D.; Crawford, E. T.; Arduengo III, A. J.; Gupton, B. F.; McQuade, D. T.; Opatz, T. Eur. J. Org. Chem. 2019, 2019, 5519–5526.

[2] Aubart, K. M.; Benowitz, A. B.; Fang, Y.; Hoffman, J.; Karpinski, J. M.; Knox, A. N.; Liao, X.; Qin, D.; Shi, D.; Spletstoser J.T. Peptide deformylase inhibitors. U.S. Patent No. 8,901,119. **Dec. 2014.**

[3] Itoh, T.; Mase, T. *Tetrahedron Lett.* **2005**, *46*, 3573–3577.

[4] Sturm, H.-J.; Armbrust, H. *Justus Liebigs Ann. Chem.* **1969**, *729*, 139–145.

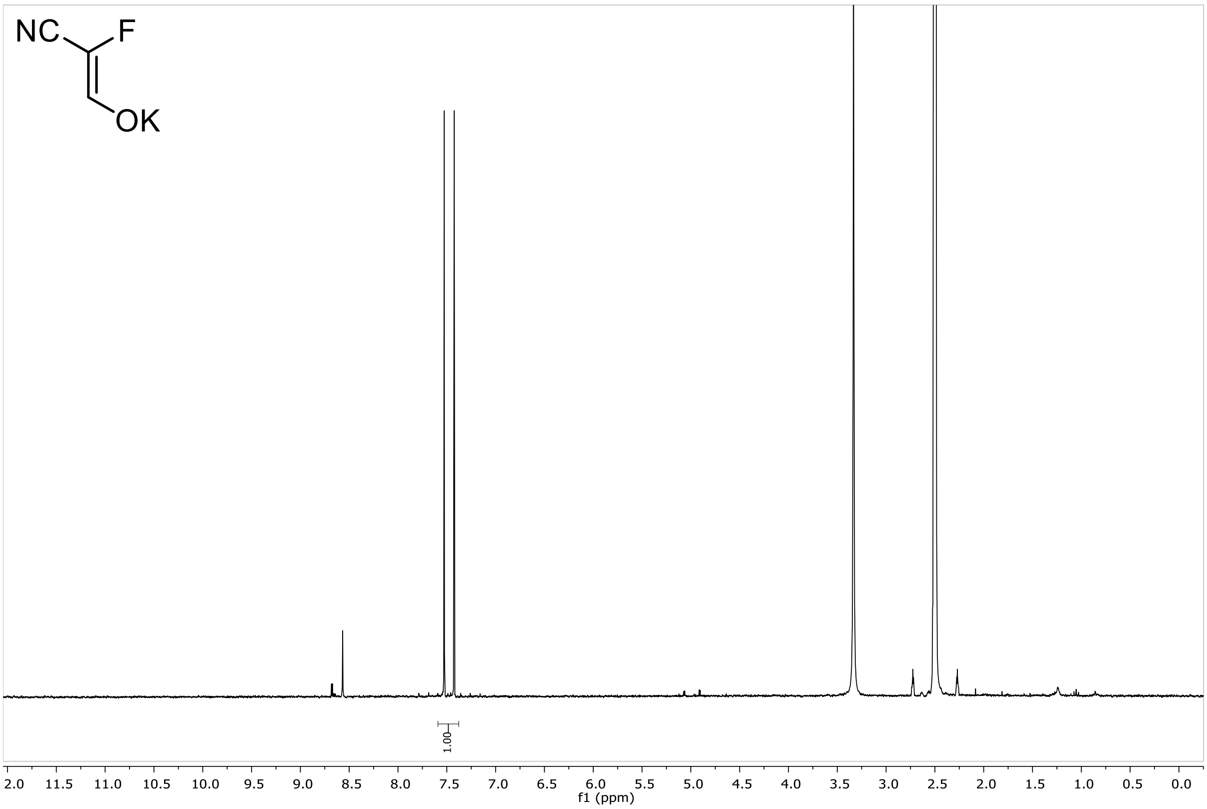

Figure S1: ^{1}H-NMR (300 MHz, DMSO-d_6): Potassium-(*Z*)-2-cyano-2-fluoroethenolate (**8**).

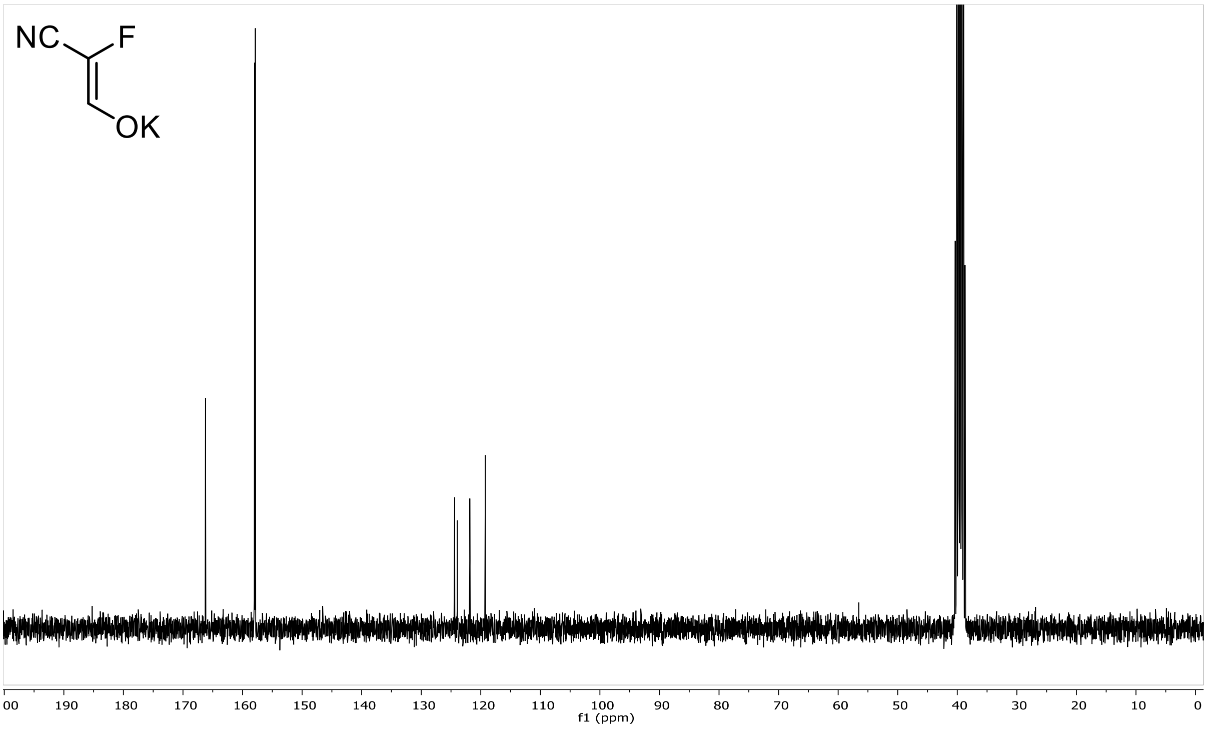

Figure S2: ^{13}C-NMR (75 MHz, DMSO-d_6): Potassium-(*Z*)-2-cyano-2-fluoroethenolate (**8**).

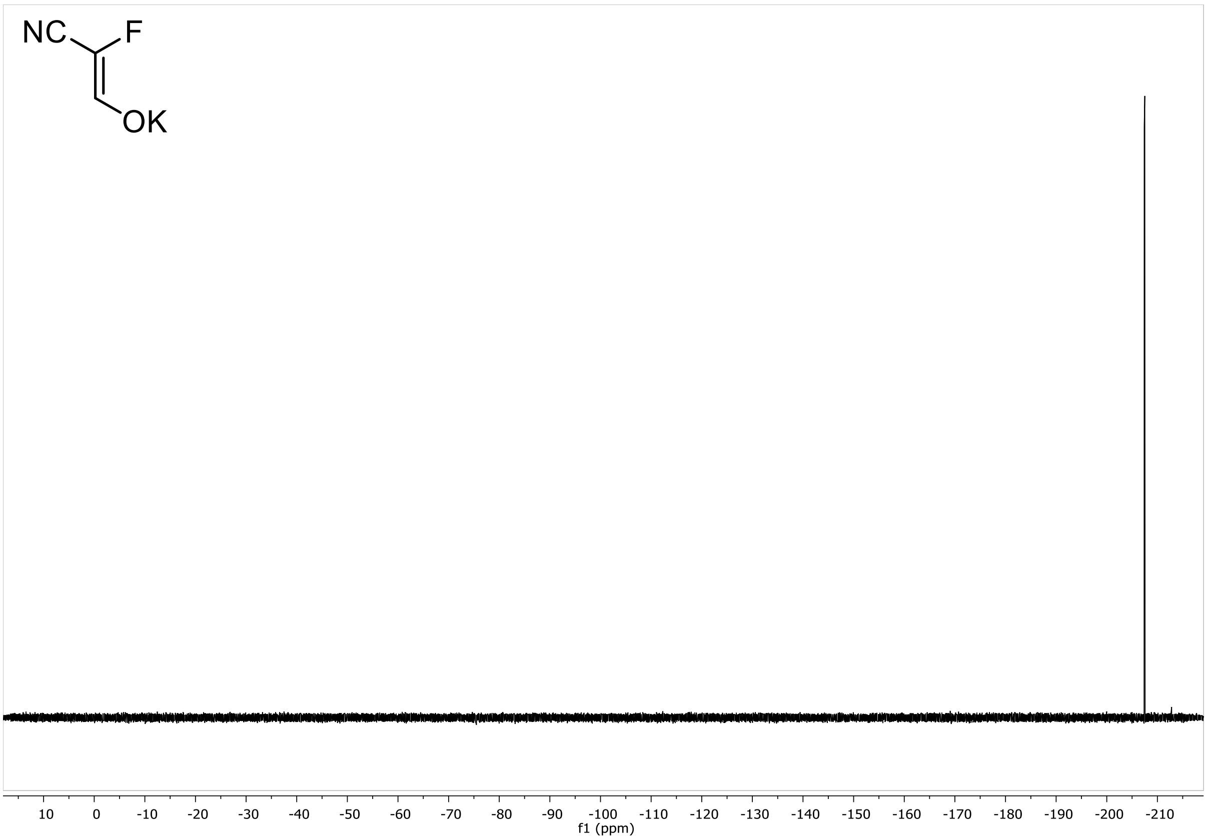

Figure S3: ^{19}F-NMR (282 MHz, DMSO-d_6): Potassium-(*Z*)-2-cyano-2-fluoroethenolate (**8**).

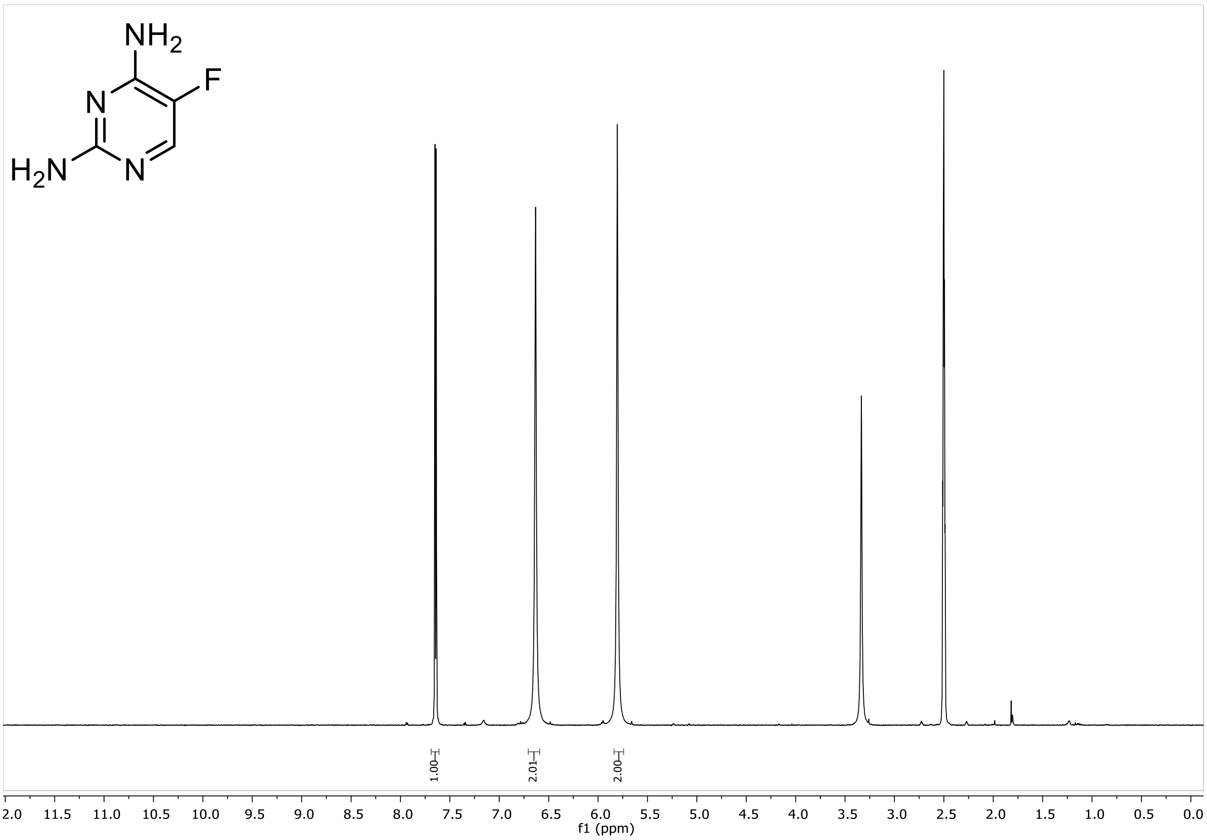

Figure S4: ^{1}H-NMR (300 MHz, DMSO-d_6): 5-Fluoropyrimidine-2,4-diamine (**10b**).

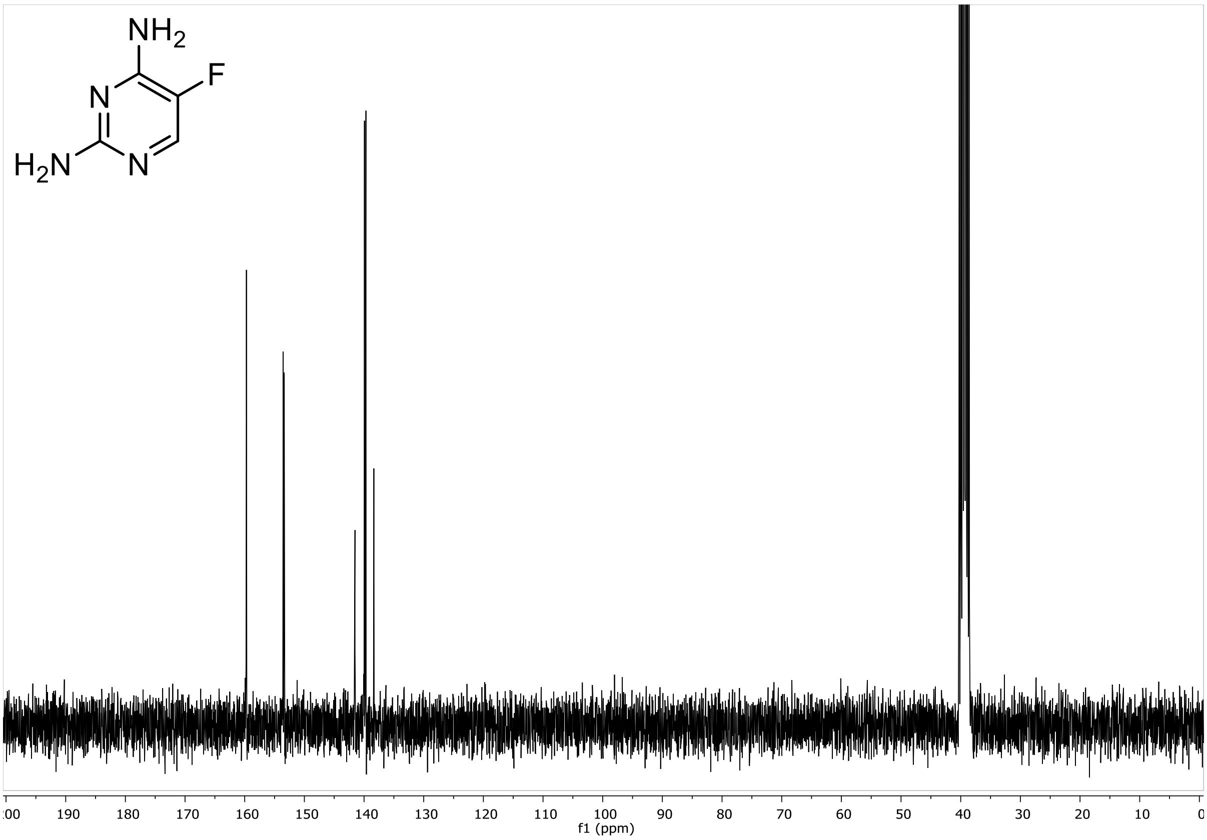

Figure S5: ^{13}C-NMR (75 MHz, DMSO-d_6): 5-Fluoropyrimidine-2,4-diamine (**10b**).

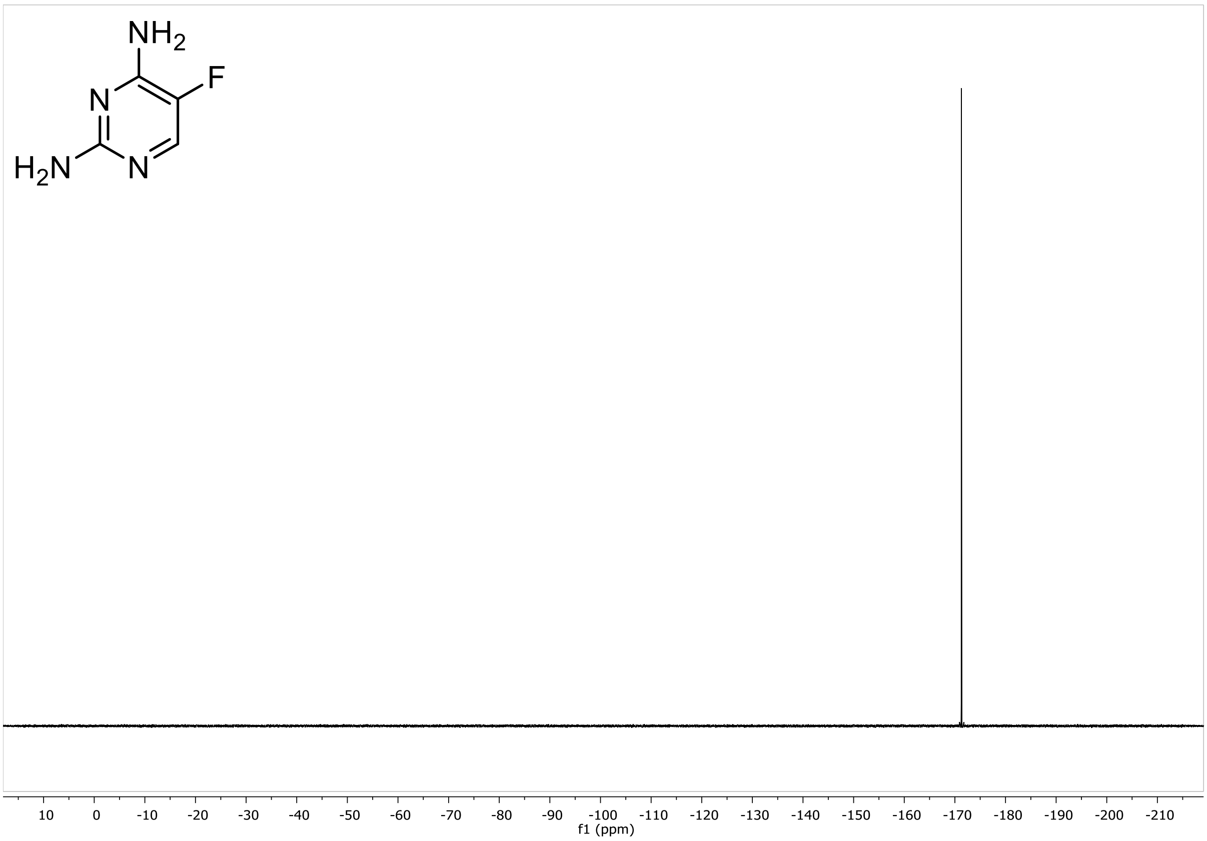

Figure S6: ^{19}F-NMR (282 MHz, DMSO-d_6): 5-Fluoropyrimidine-2,4-diamine (**10b**).

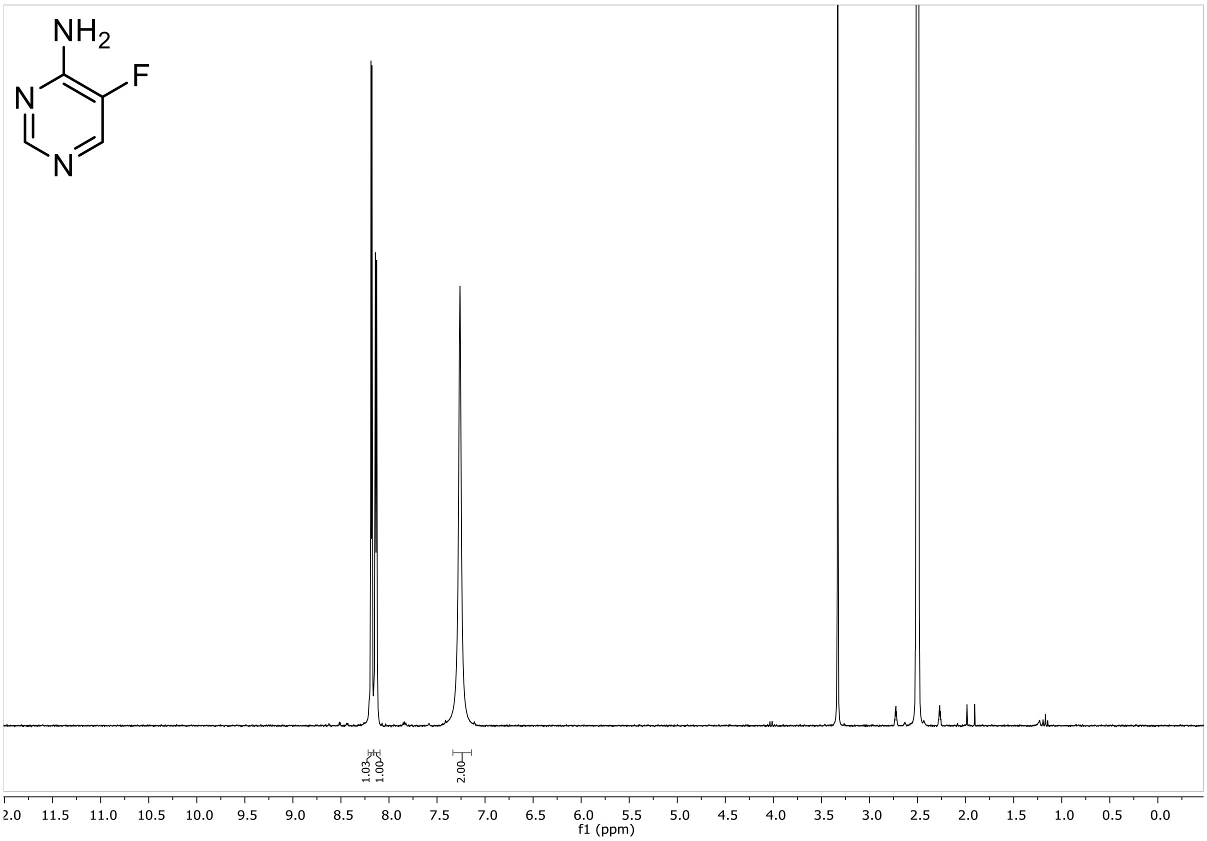

Figure S7: ^{1}H-NMR (300 MHz, DMSO-d_6): 5-Fluoropyrimidine-4-amine (**10a**).

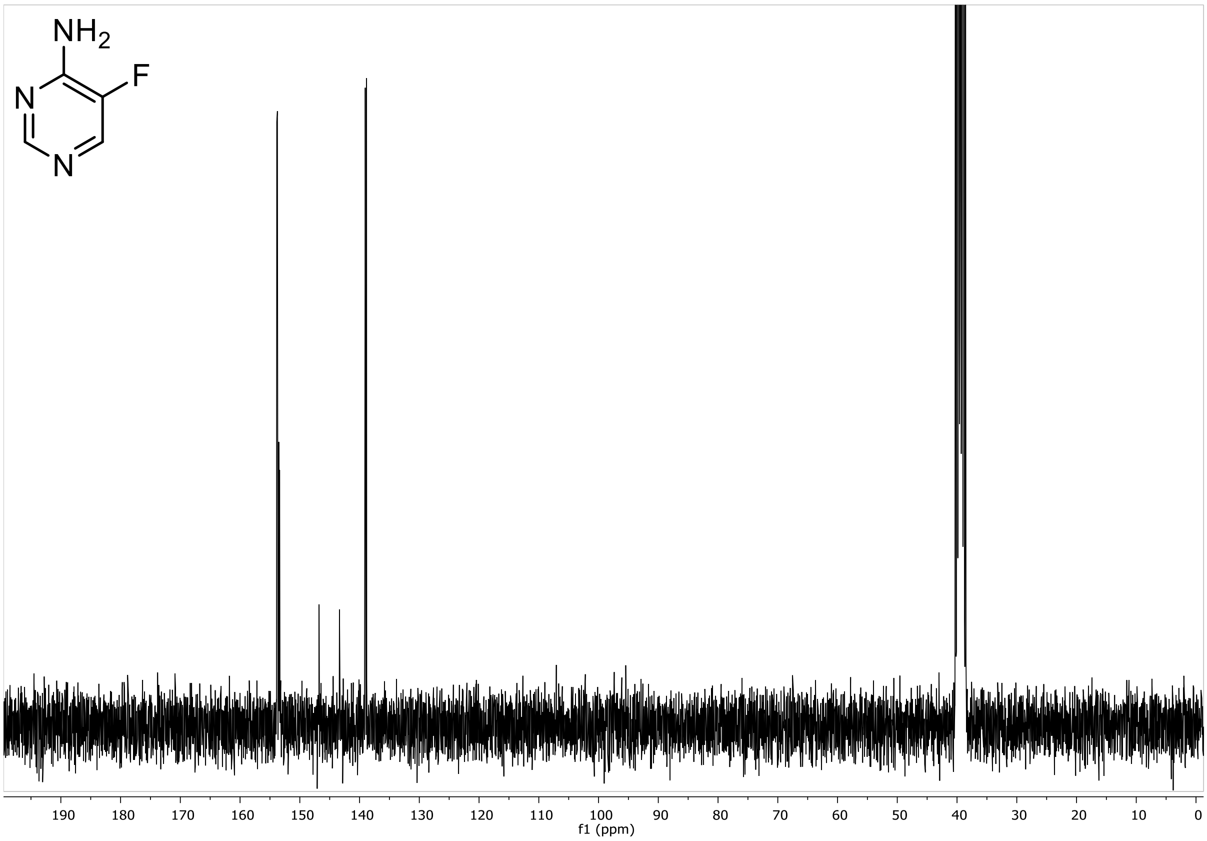

Figure S8: ^{13}C-NMR (75 MHz, DMSO-d_6): 5-Fluoropyrimidine-4-amine (**10a**).

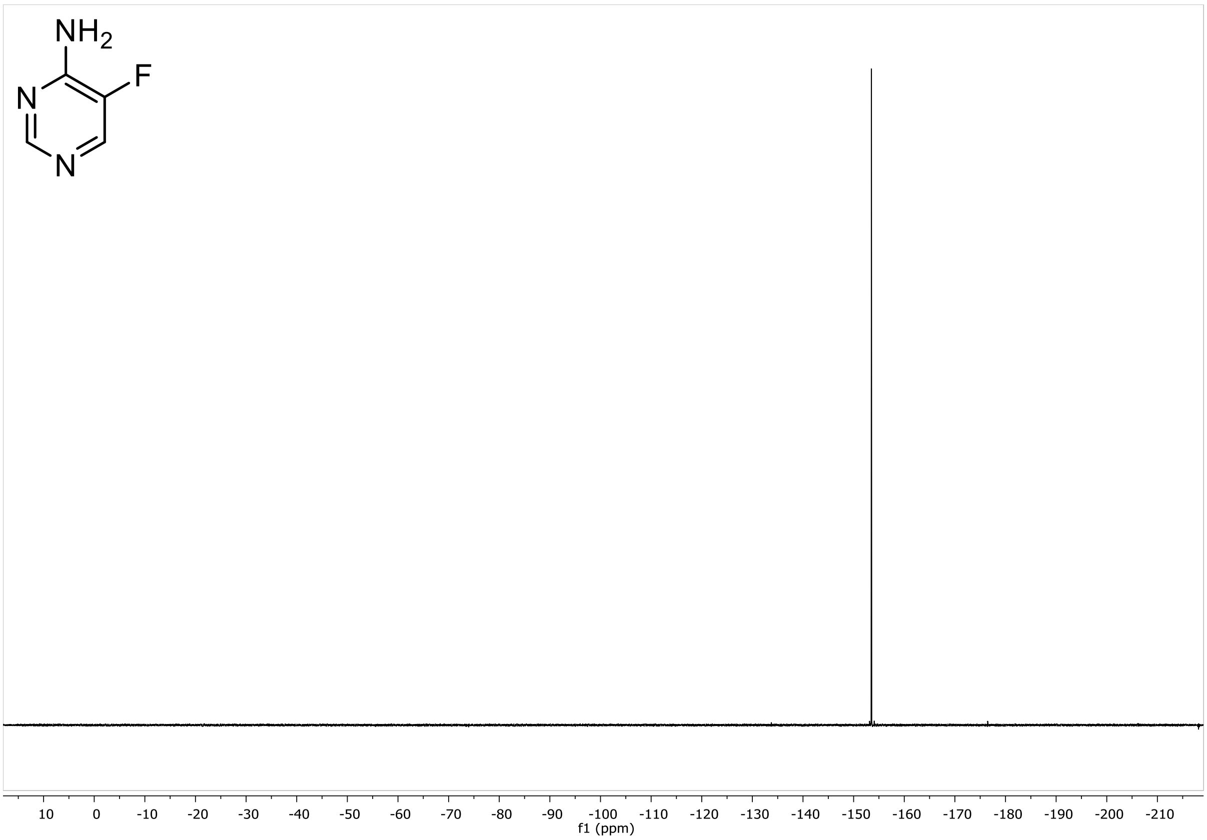

Figure S9: ^{19}F-NMR (282 MHz, DMSO-d6): 5-Fluoropyrimidine-4-amine (**10a**).

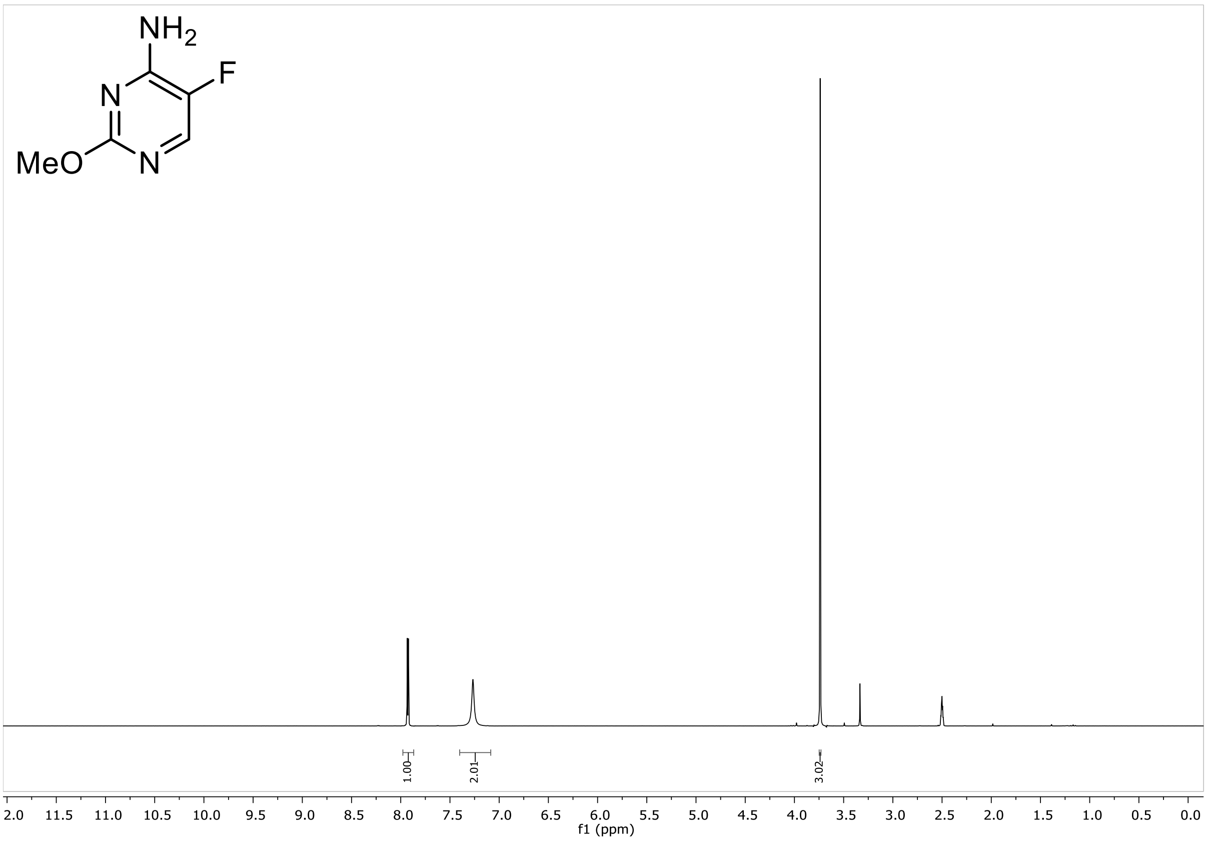

Figure S10: ^{1}H-NMR (300 MHz, DMSO-d6): 5-Fluoro-2-methoxypyrimidine-4-amine (**10c**).

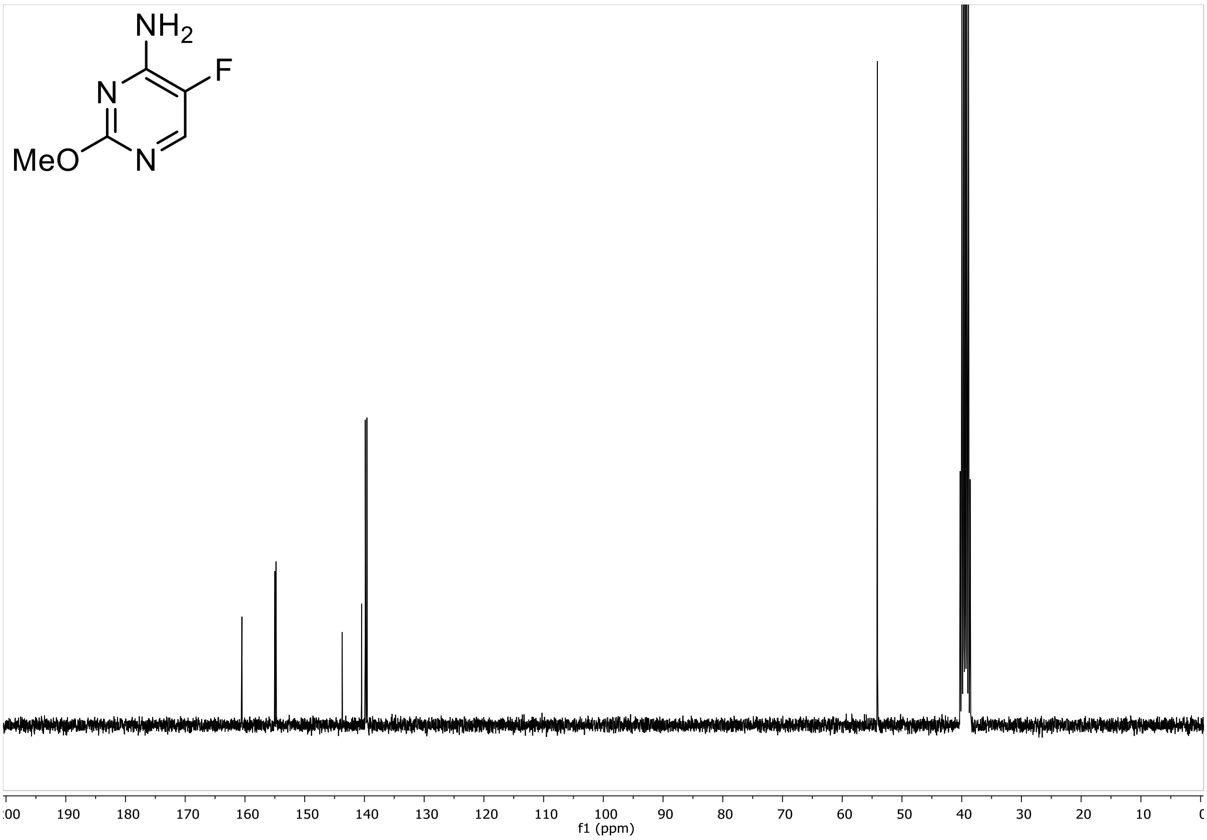

Figure S11: ^{13}C-NMR (75 MHz, DMSO-d$_6$): 5-Fluoro-2-methoxypyrimidine-4-amine (**10c**).

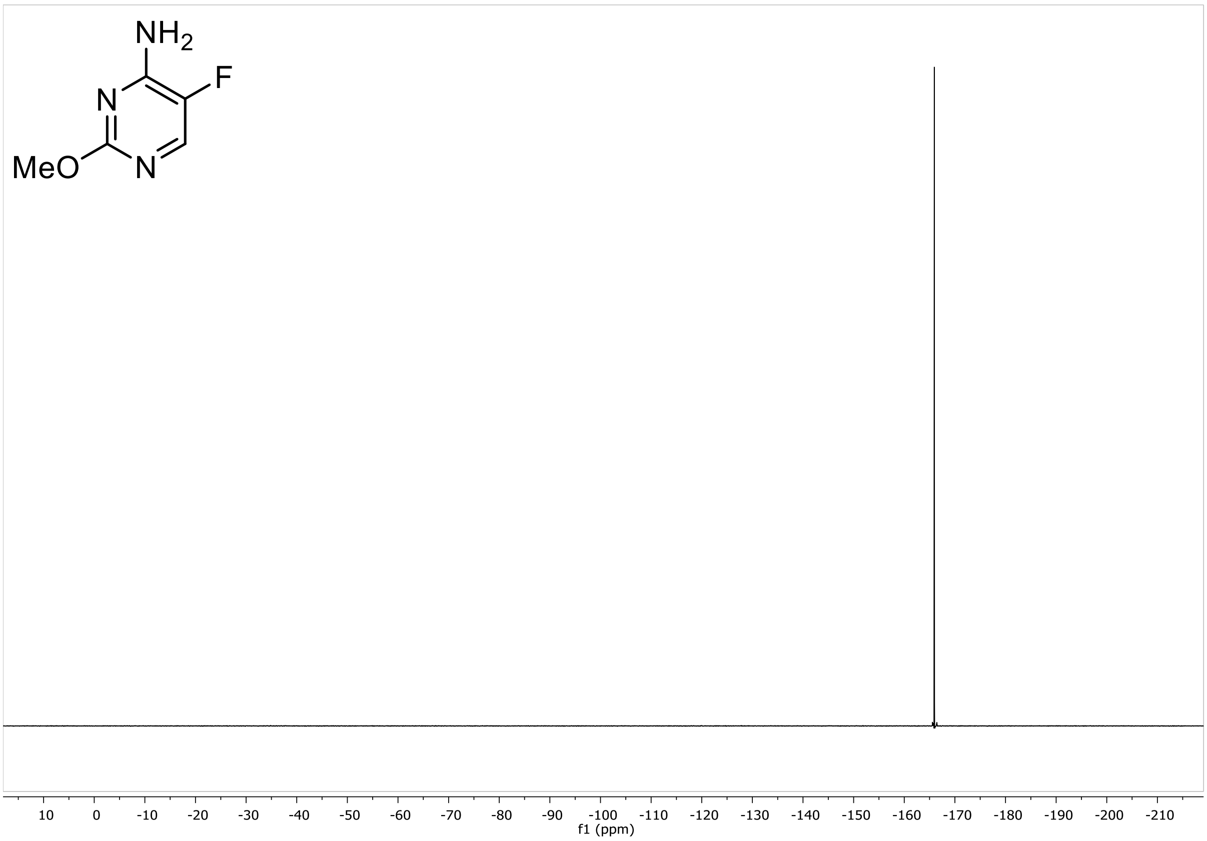

Figure S12: ^{19}F-NMR (282 MHz, DMSO-d$_6$): 5-Fluoro-2-methoxypyrimidine-4-amine (**10c**).

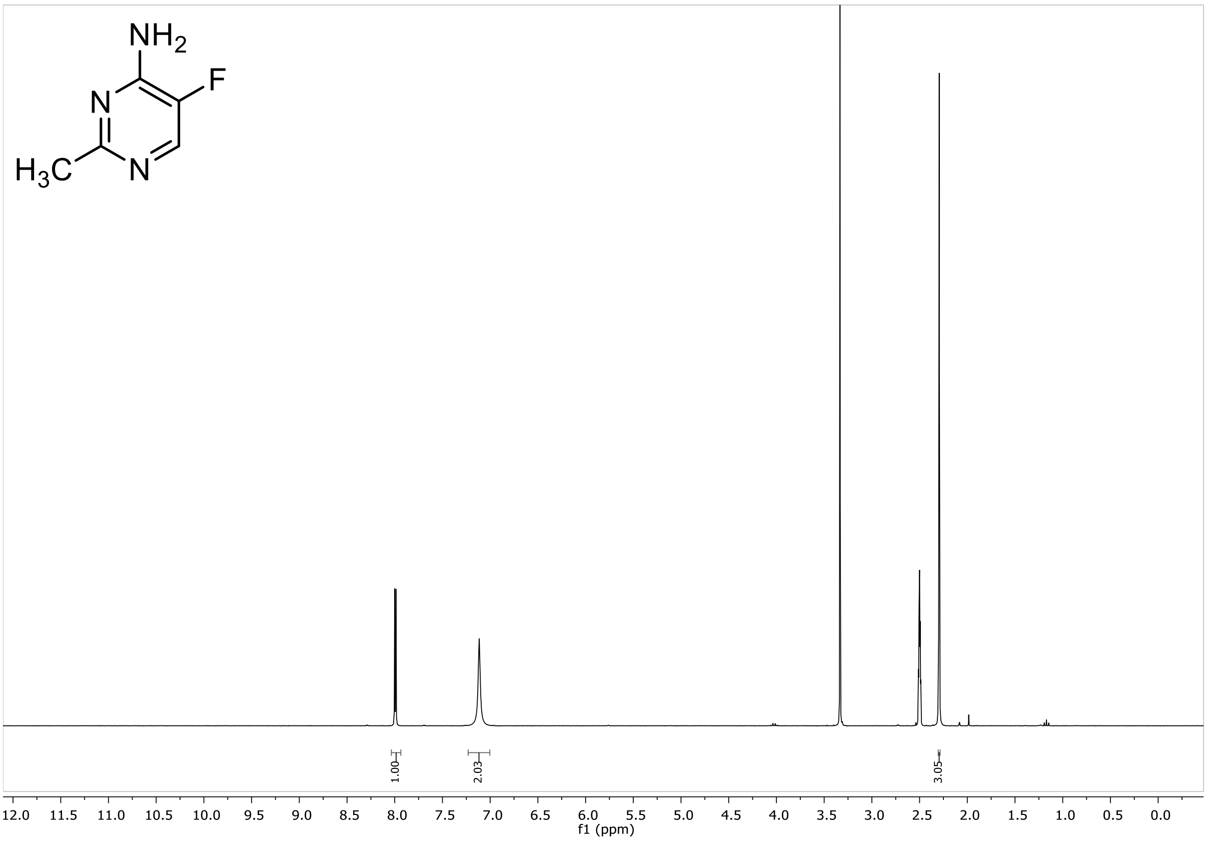

Figure S13: ^{1}H-NMR (300 MHz, DMSO-d_6): 5-Fluoro-2-methylpyrimidine-4-amine (**10d**).

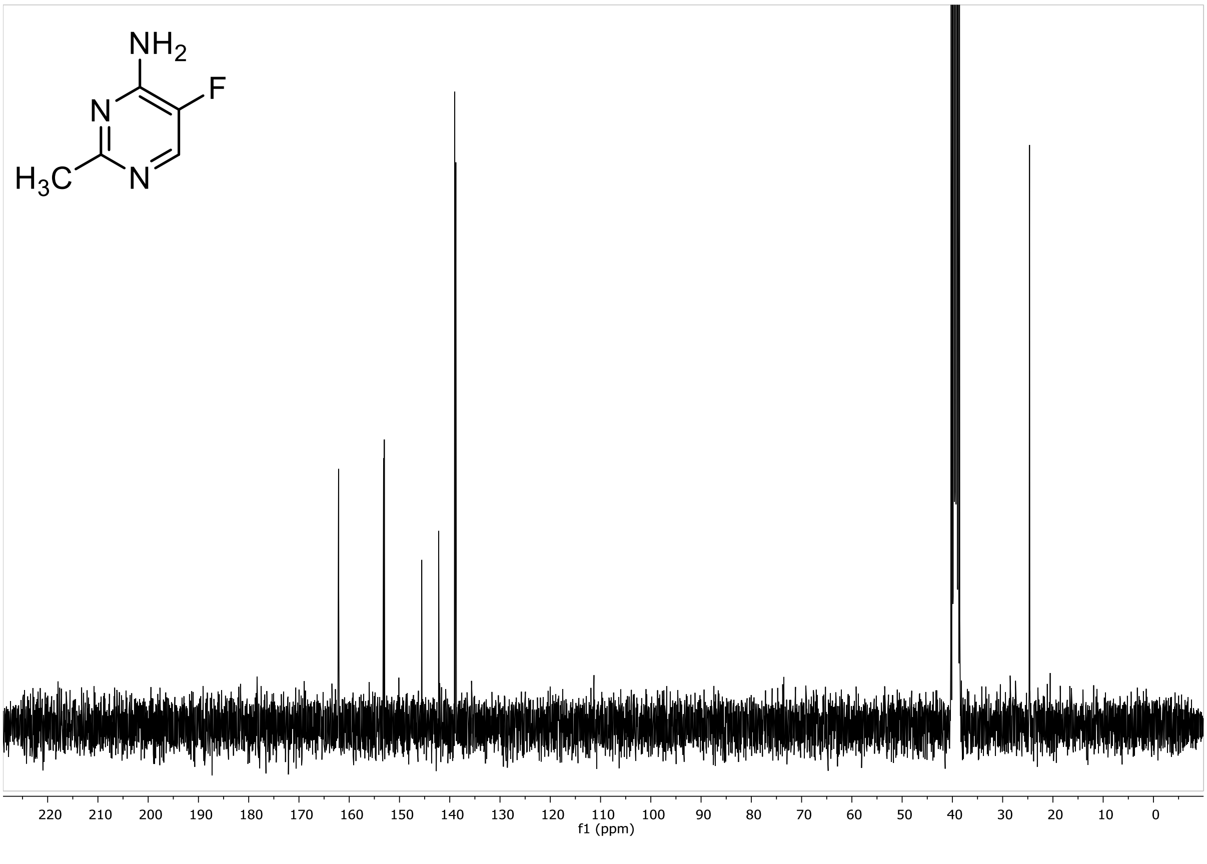

Figure S14: ^{13}C-NMR (75 MHz, DMSO-d_6): 5-Fluoro-2-methylpyrimidine-4-amine (**10d**).

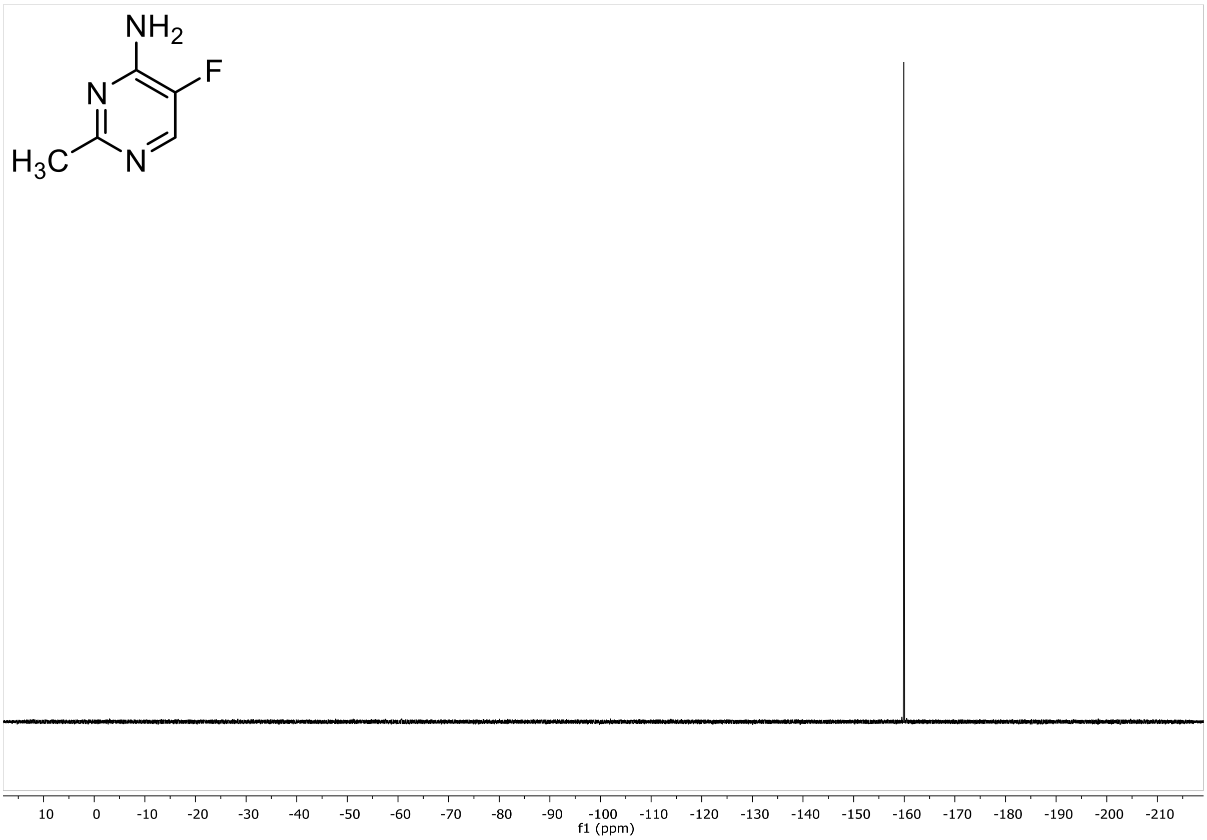

Figure S15: ^{19}F-NMR (282 MHz, DMSO-d_6): 5-Fluoro-2-methylpyrimidine-4-amine (**10d**).

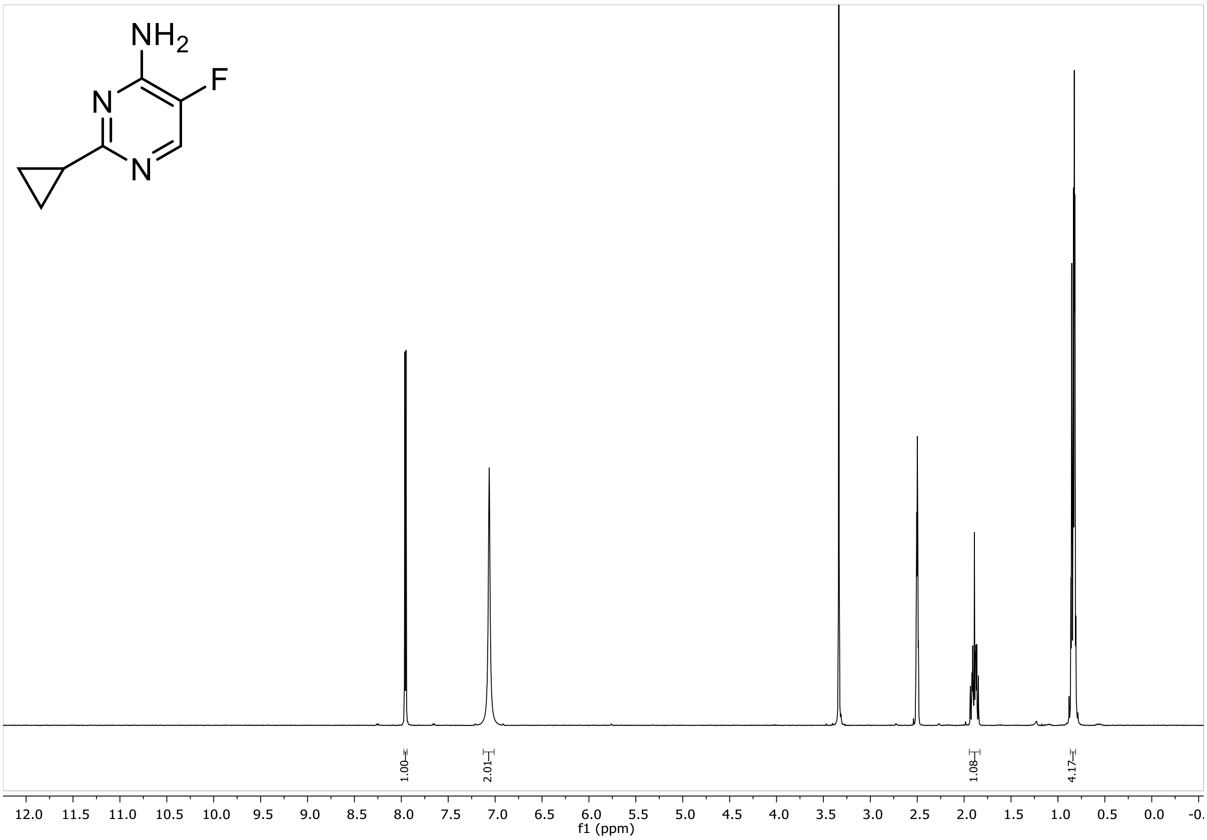

Figure S16: ^{1}H-NMR (300 MHz, DMSO-d_6): 2-Cyclopropyl-5-fluoropyrimidine-4-amine (**10e**).

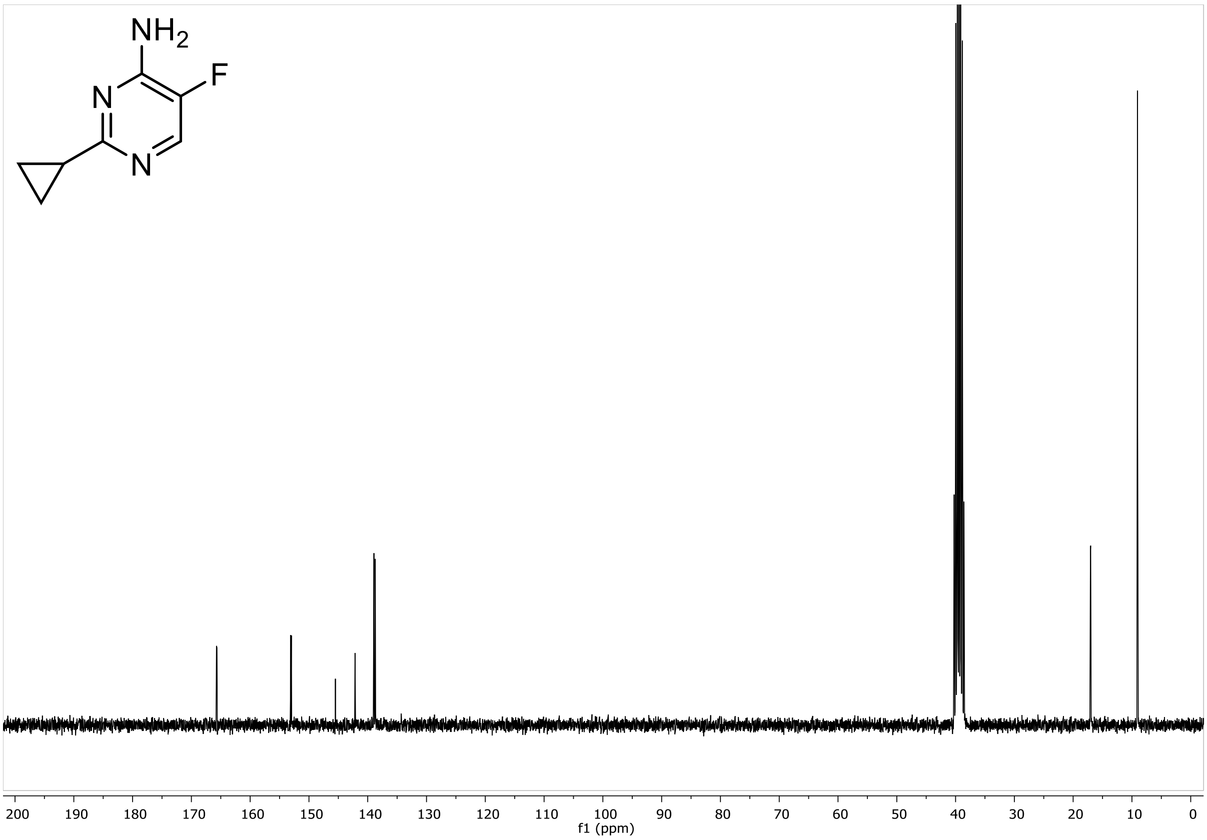

Figure S17: ^{13}C-NMR (75 MHz, DMSO-d_6): 2-Cyclopropyl-5-fluoropyrimidine-4-amine (**10e**).

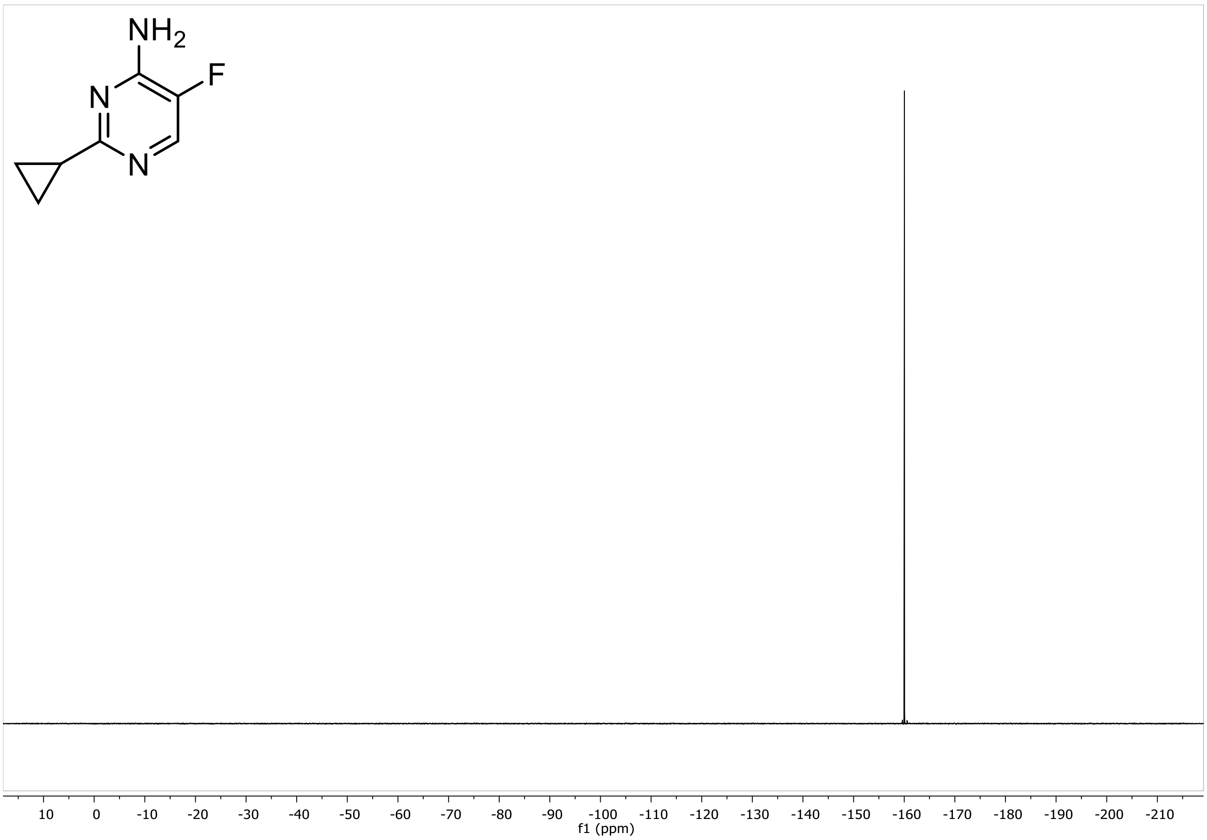

Figure S18: ^{19}F-NMR (282 MHz, DMSO-d_6): 2-Cyclopropyl-5-fluoropyrimidine-4-amine (**10e**).

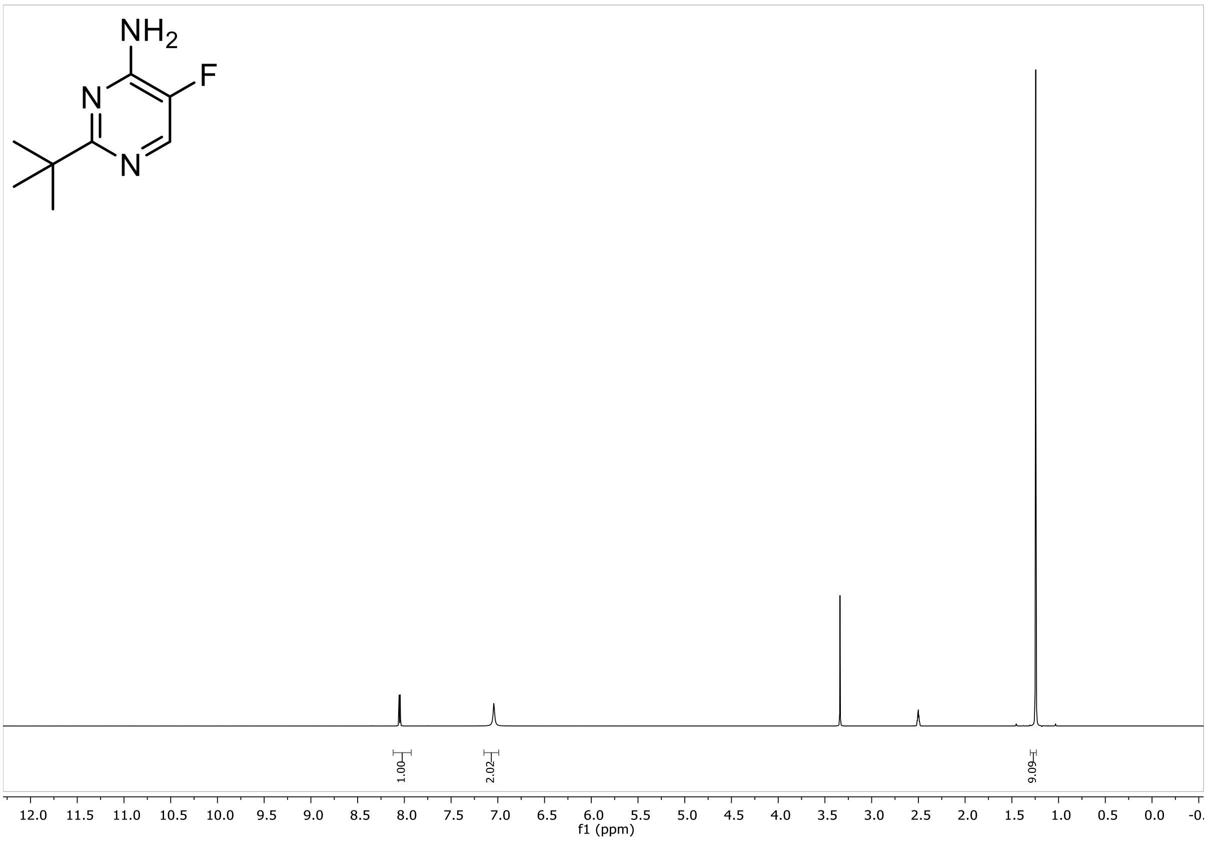

Figure S19: ^{1}H-NMR (300 MHz, DMSO-d6): 2-*tert*-Butyl-5-fluoropyrimidine-4-amine (**10f**).

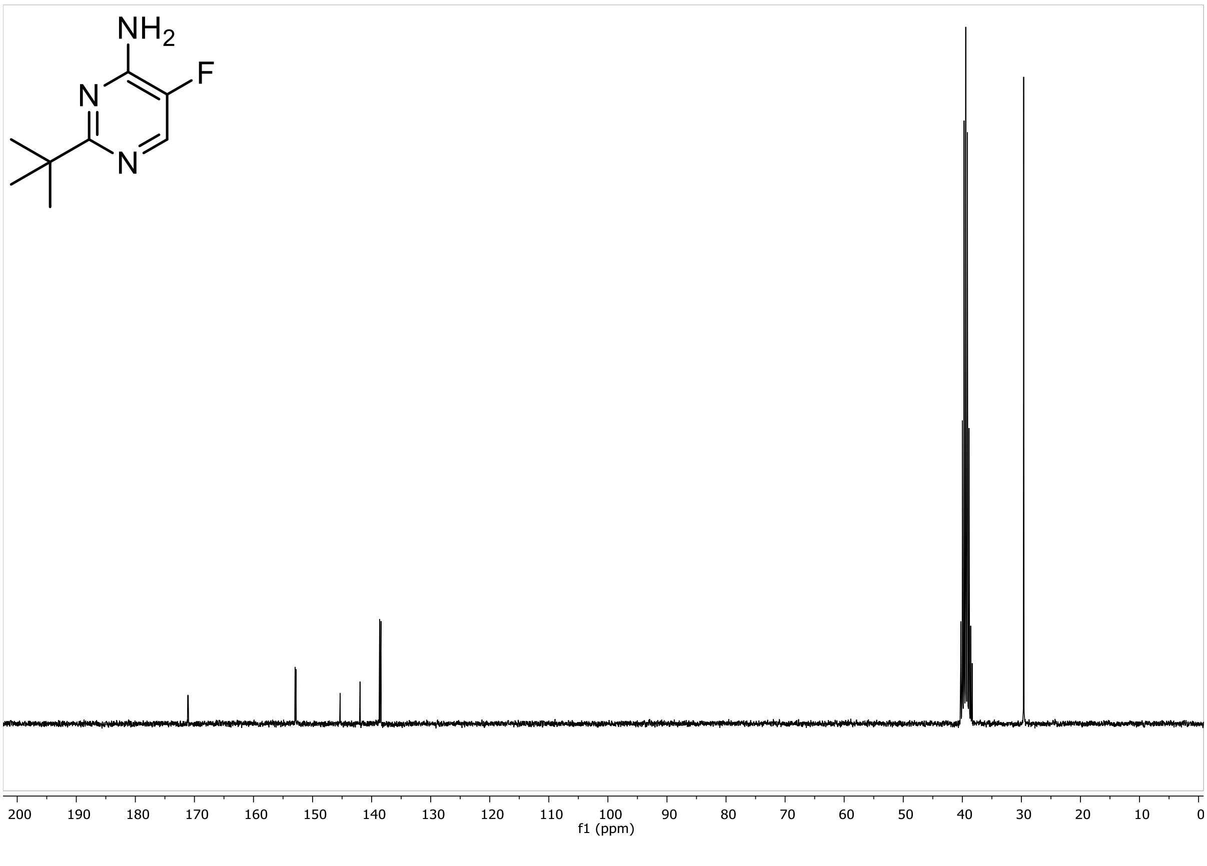

Figure S20: ^{13}C-NMR (75 MHz, DMSO-d6): 2-*tert*-Butyl-5-fluoropyrimidine-4-amine (**10f**).

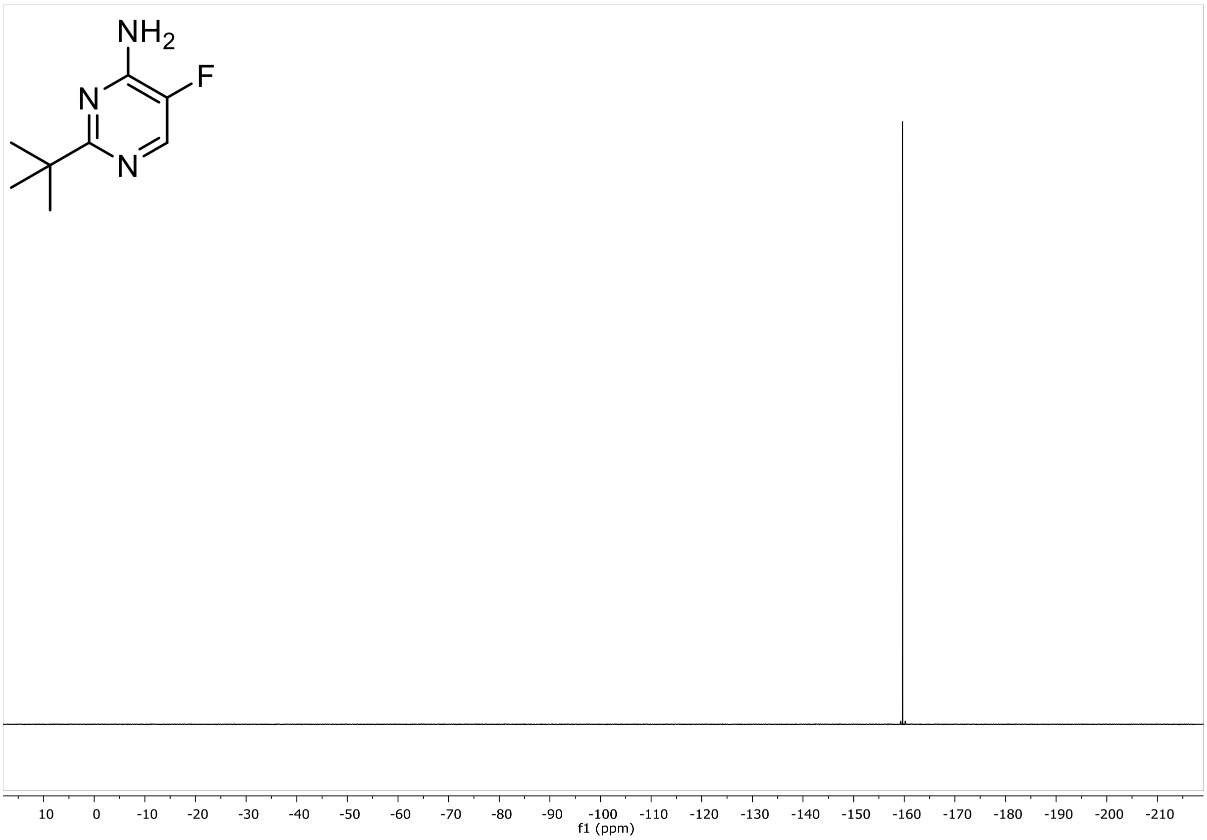

Figure S21: ^{19}F-NMR (282 MHz, DMSO-d$_6$): 2-*tert*-Butyl-5-fluoropyrimidine-4-amine (**10f**).

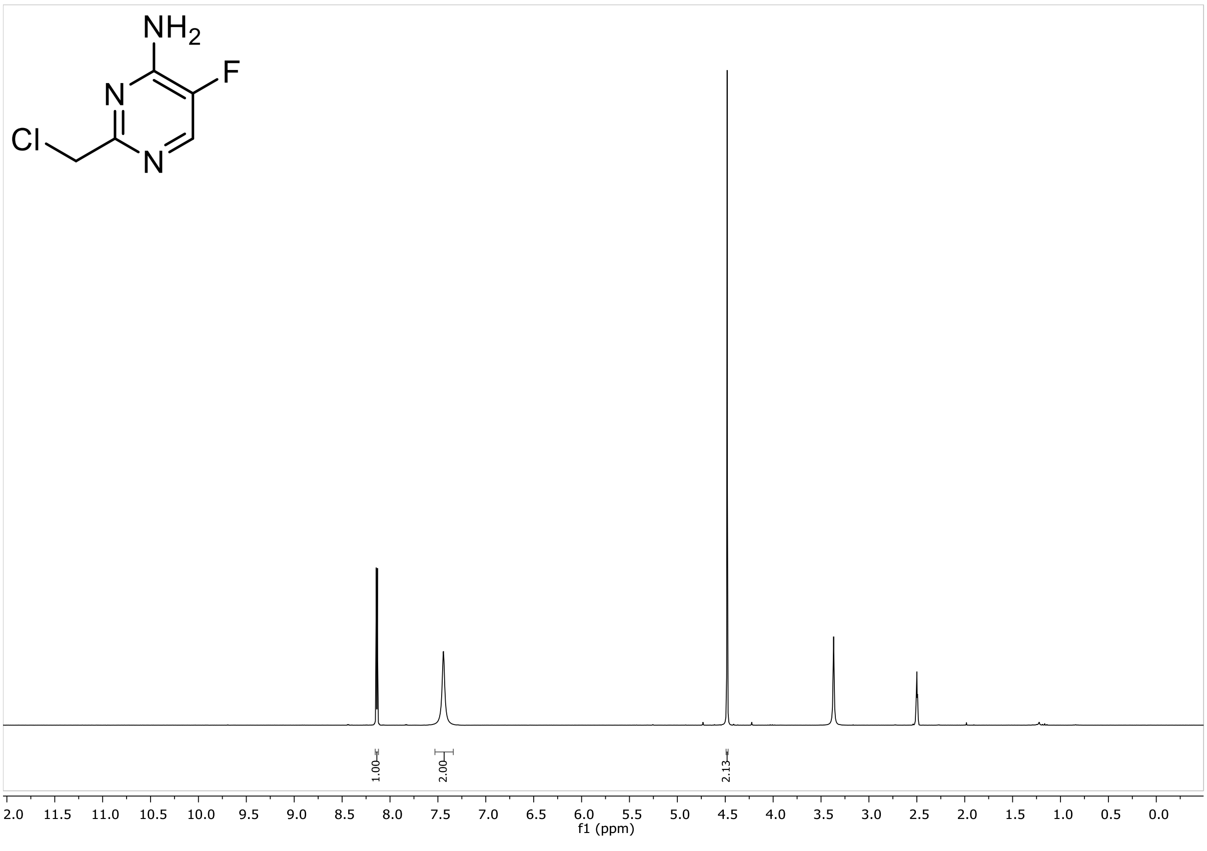

Figure S22: ^{1}H-NMR (300 MHz, DMSO-d$_6$): 2-(Chloromethyl)-5-fluoropyrimidine-4-amine (**10g**).

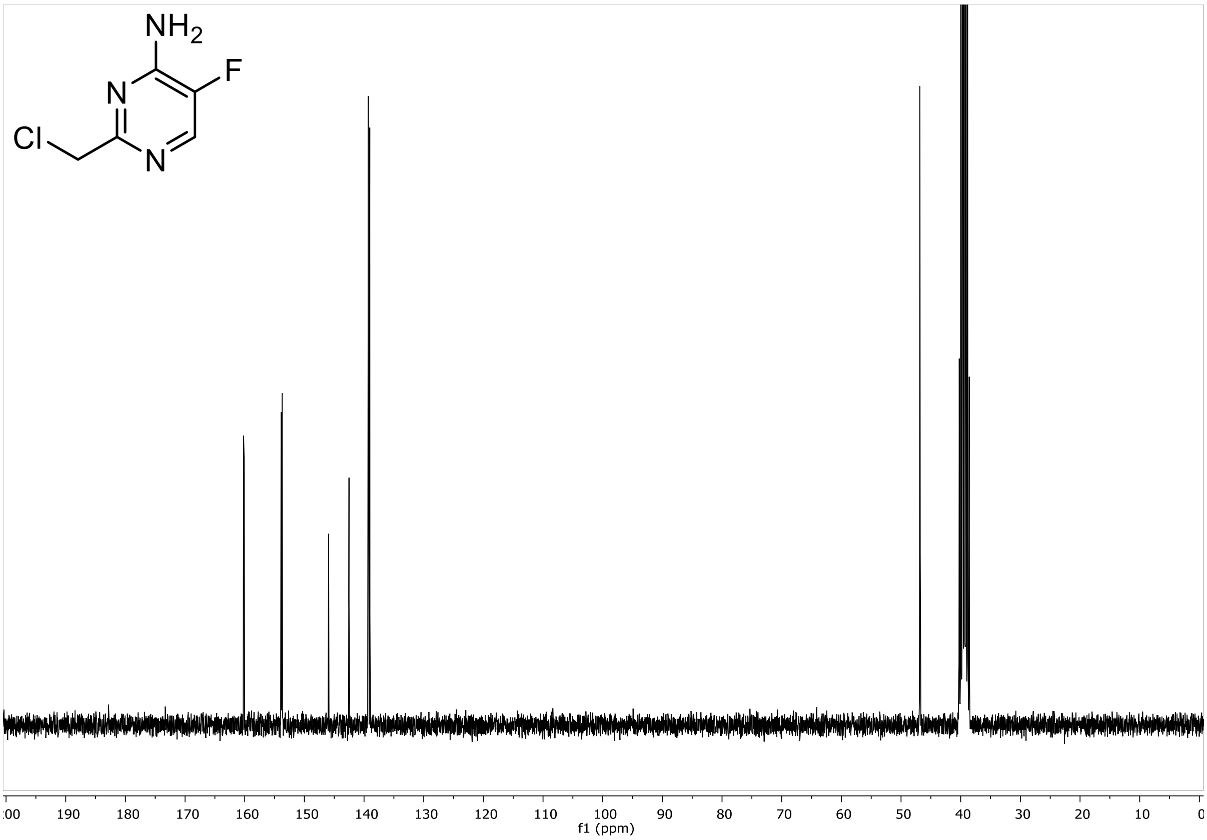

Figure S23: ^{13}C-NMR (75 MHz, DMSO-d_6): 2-(Chloromethyl)-5-fluoropyrimidine-4-amine (**10g**).

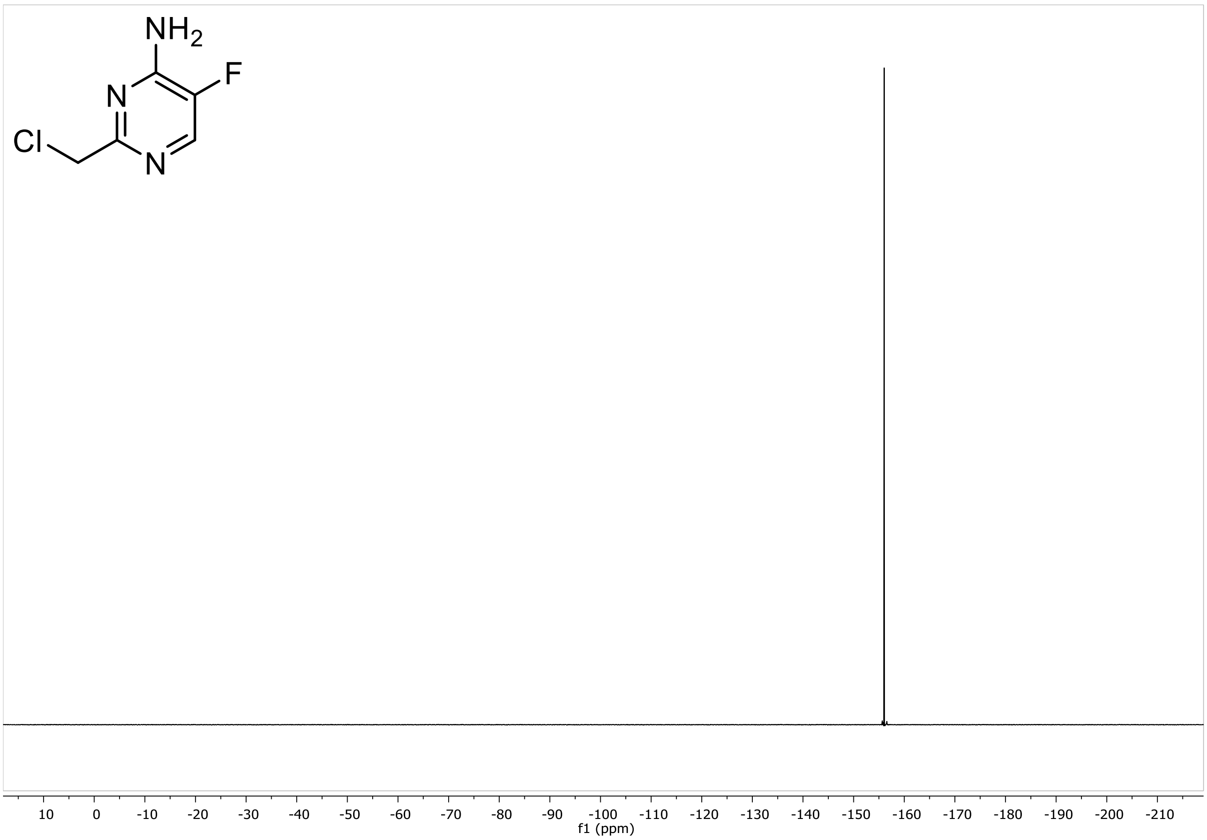

Figure S24: ^{19}F-NMR (282 MHz, DMSO-d_6): 2-(Chloromethyl)-5-fluoropyrimidine-4-amine (**10g**).

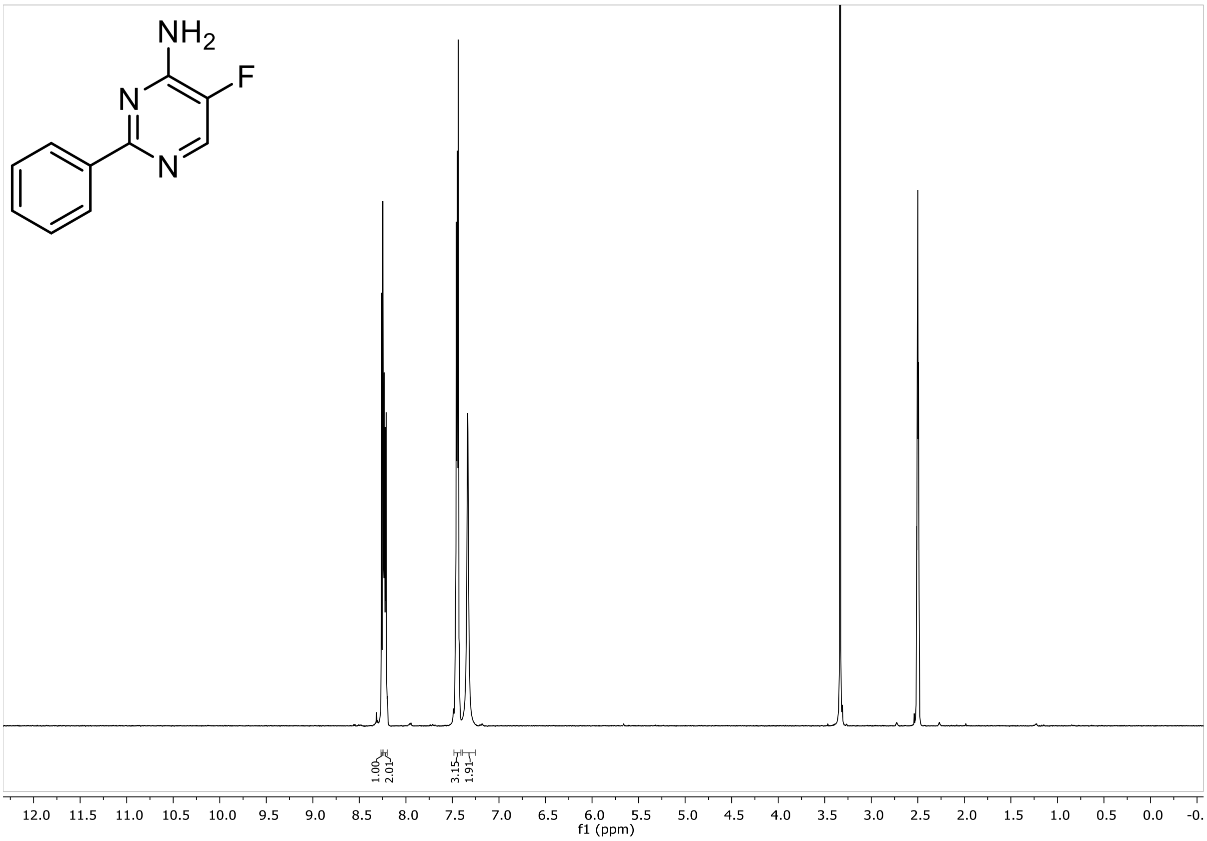

Figure S25: ^{1}H-NMR (300 MHz, DMSO-d_6): 5-Fluoro-2-phenylpyrimidine-4-amine (**10h**).

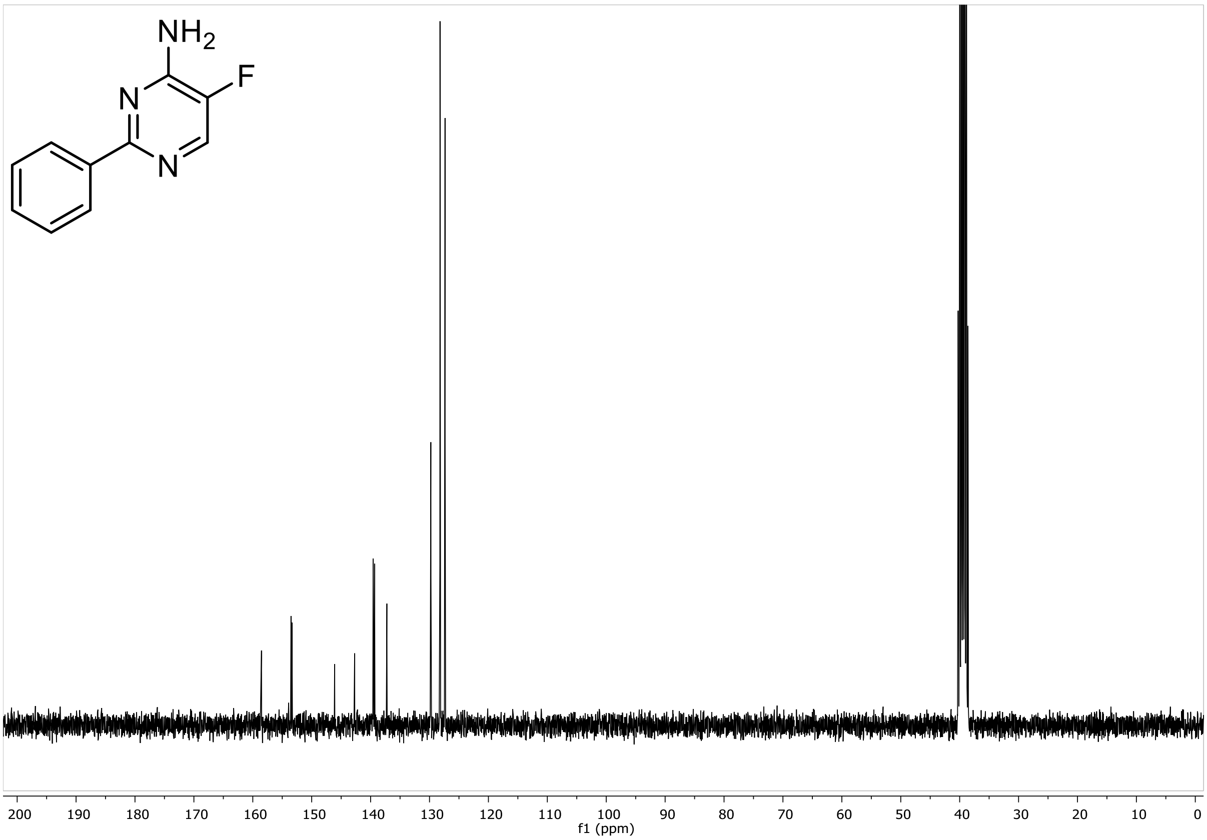

Figure S26: ^{13}C-NMR (75 MHz, DMSO-d_6): 5-Fluoro-2-phenylpyrimidine-4-amine (**10h**).

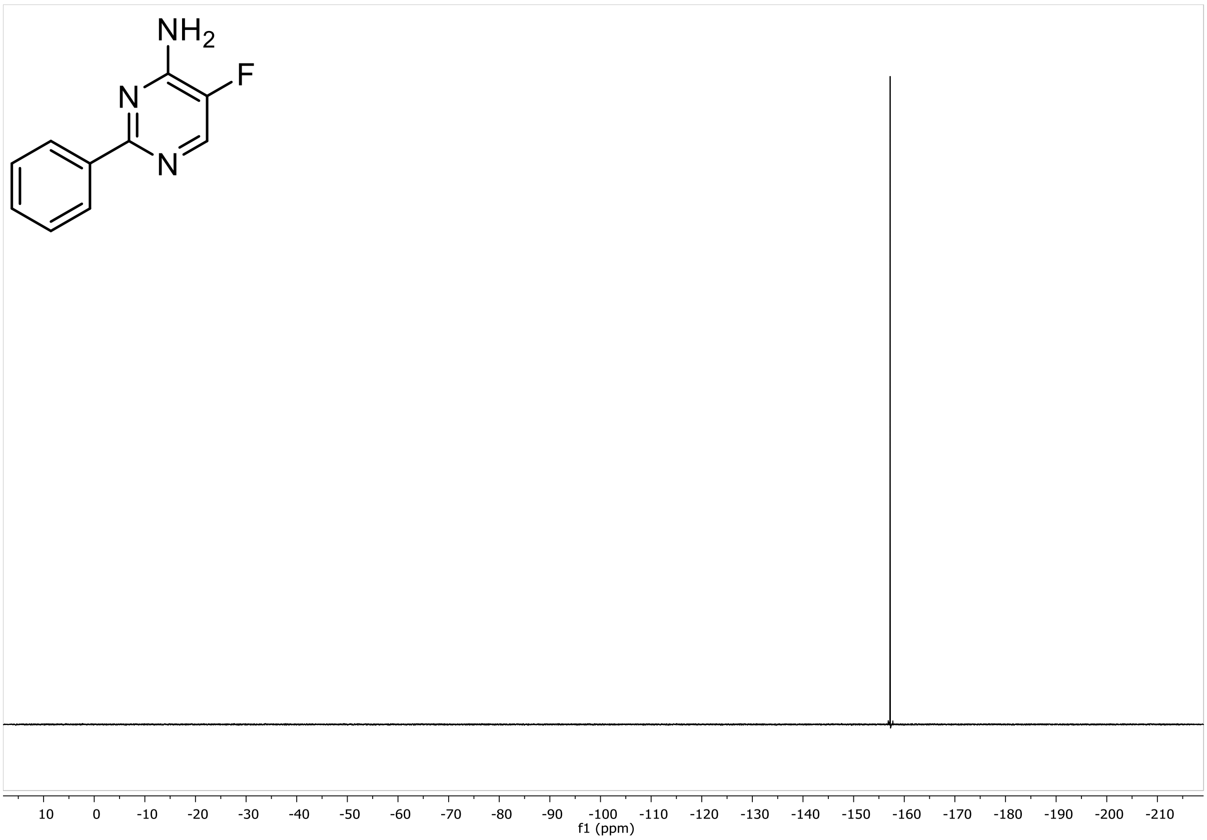

Figure S27: ^{19}F-NMR (282 MHz, DMSO-d6): 5-Fluoro-2-phenylpyrimidine-4-amine (**10h**).

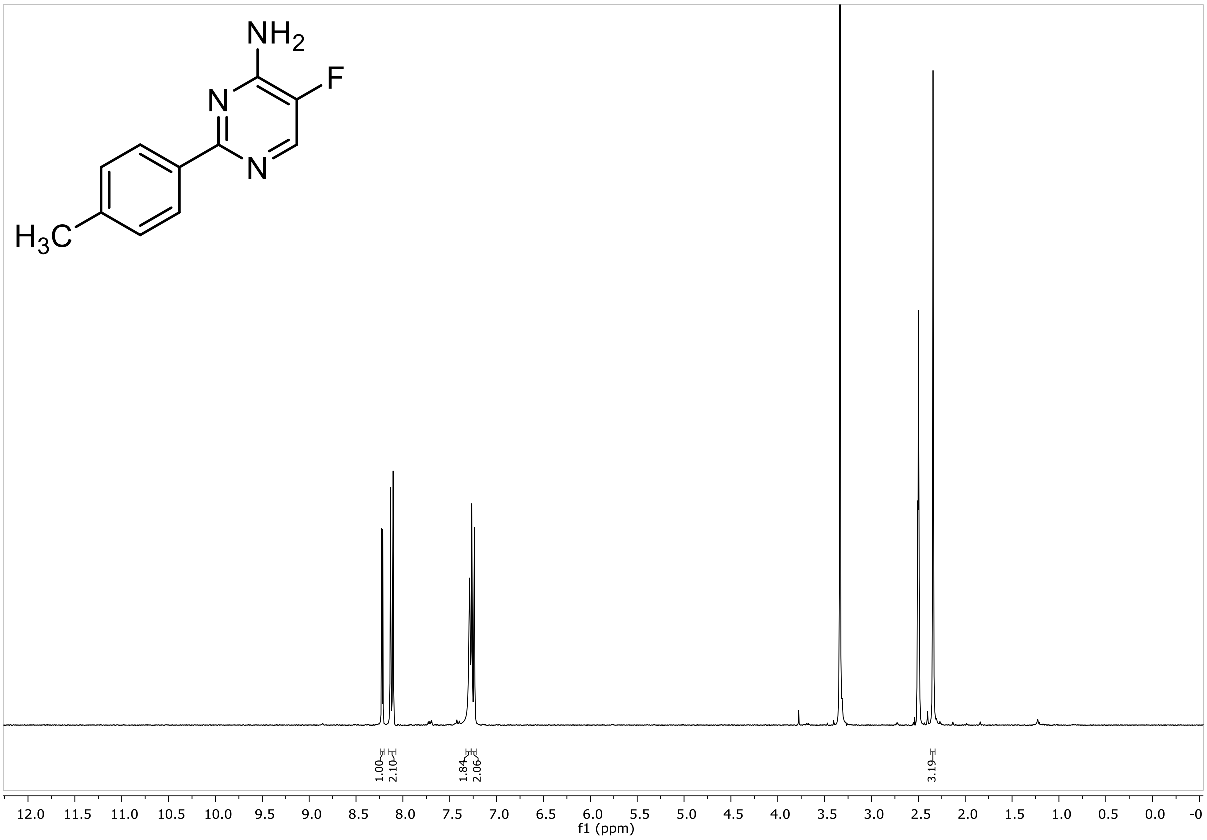

Figure S28: ^{1}H-NMR (300 MHz, DMSO-d6): 5-Fluoro-2-(4-methylphenyl)pyrimidine-4-amine (**10i**).

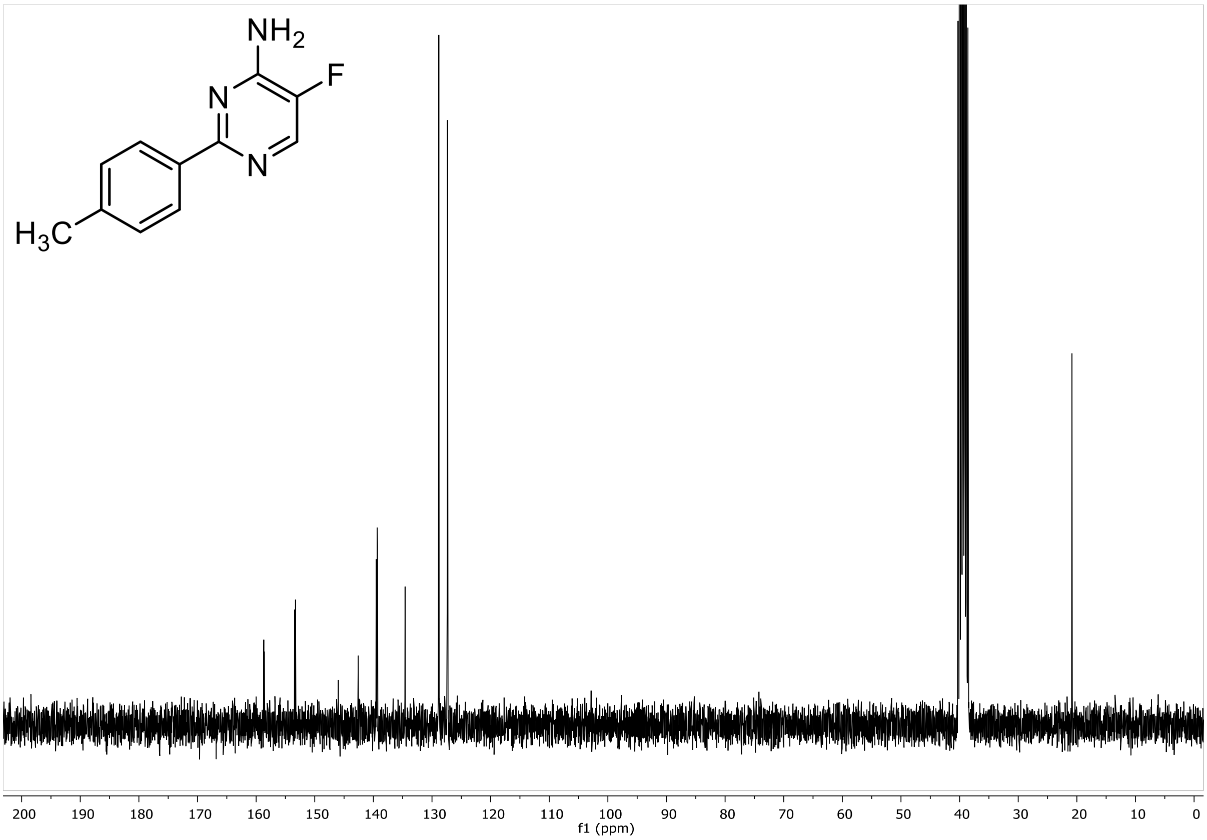

Figure S29: ^{13}C-NMR (75 MHz, DMSO-d_6): 5-Fluoro-2-(4-methylphenyl)pyrimidine-4-amine (**10i**).

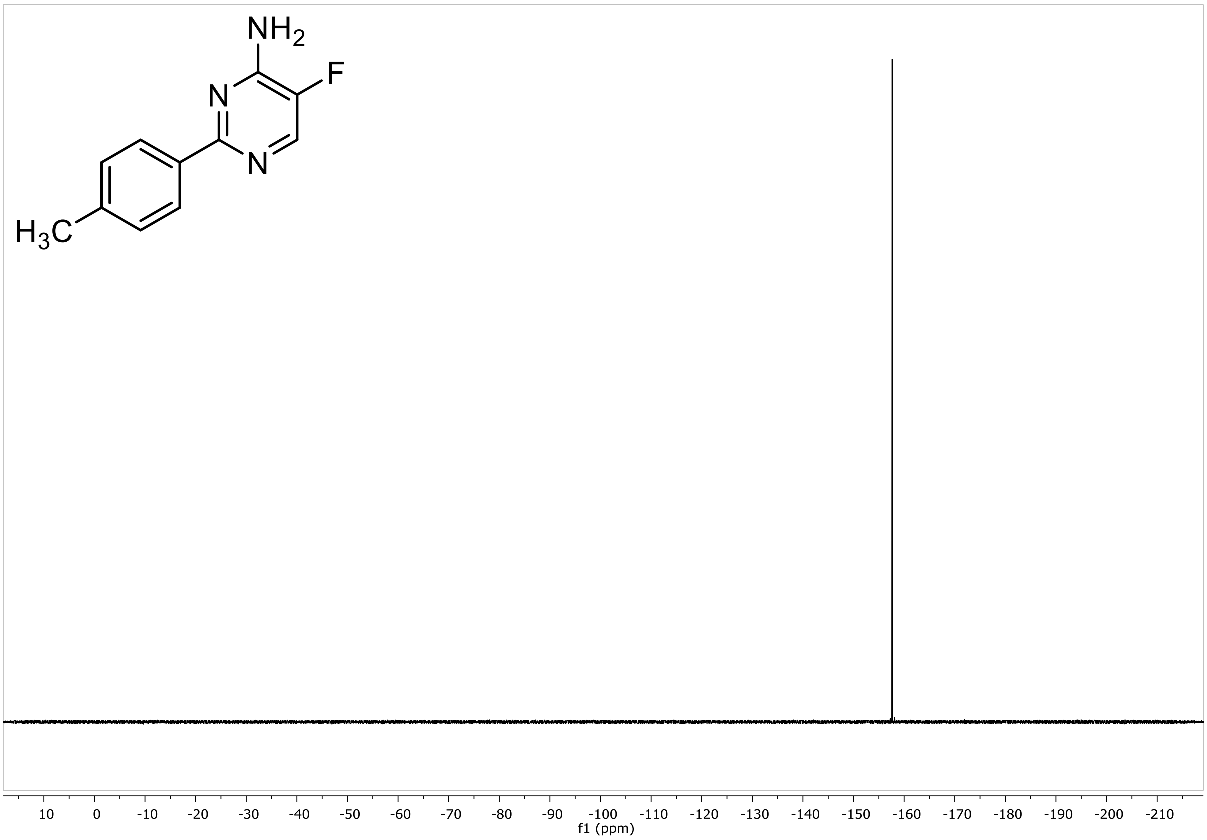

Figure S30: ^{19}F-NMR (282 MHz, DMSO-d_6): 5-Fluoro-2-(4-methylphenyl)pyrimidine-4-amine (**10i**).

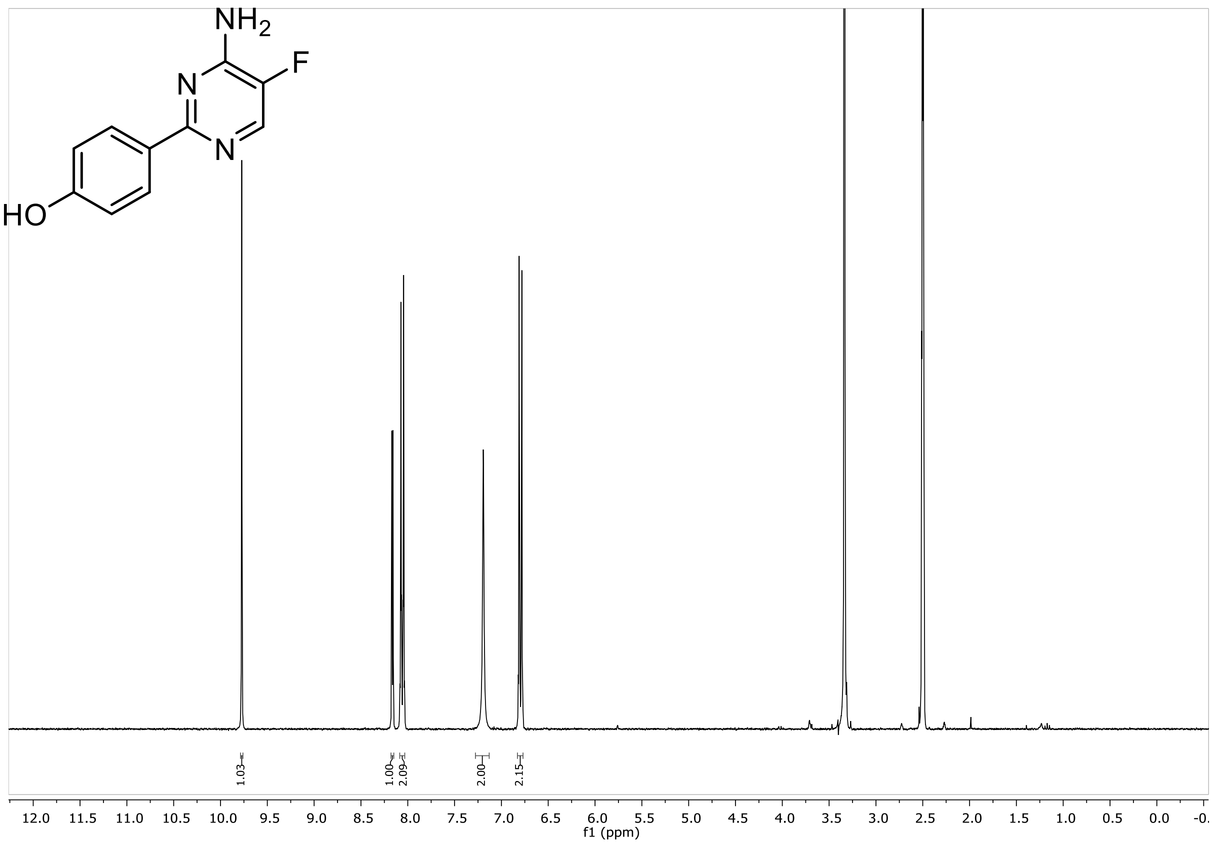

Figure S31: ^{1}H-NMR (300 MHz, DMSO-d6): 4-(4-Amine-5-fluoropyrimidine-2-yl)phenol (**10j**).

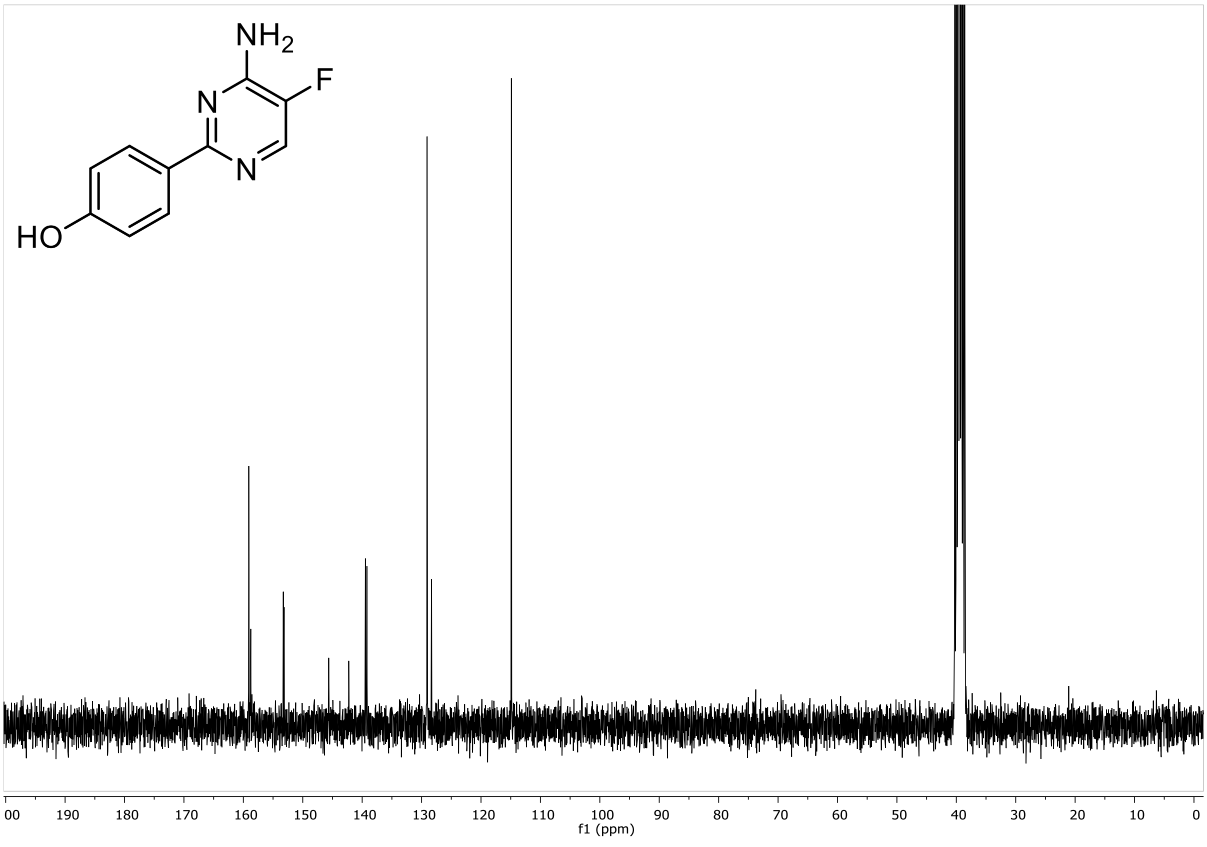

Figure S32: ^{13}C-NMR (75 MHz, DMSO-d6): 4-(4-Amine-5-fluoropyrimidine-2-yl)phenol (**10j**).

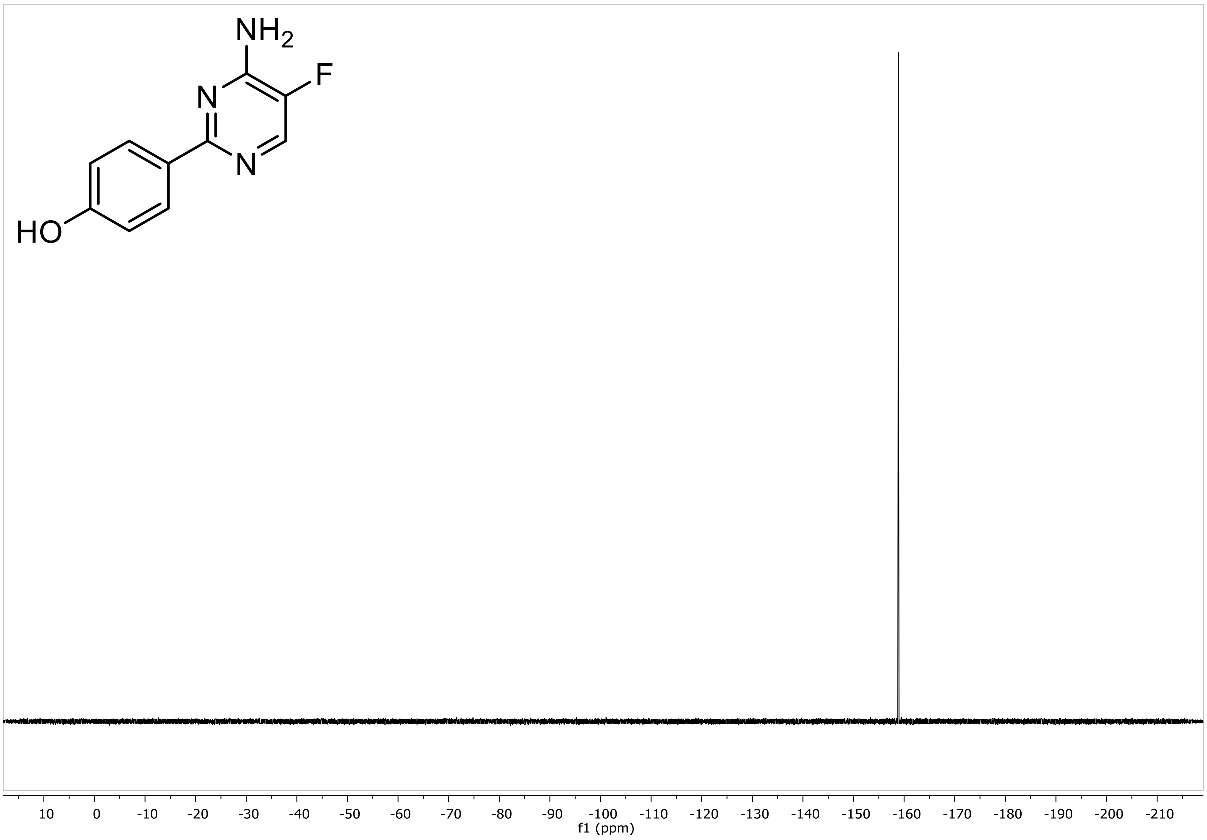

Figure S33: ^{19}F-NMR (282 MHz, DMSO-d6): 4-(4-Amine-5-fluoropyrimidine-2-yl)phenol (**10j**).

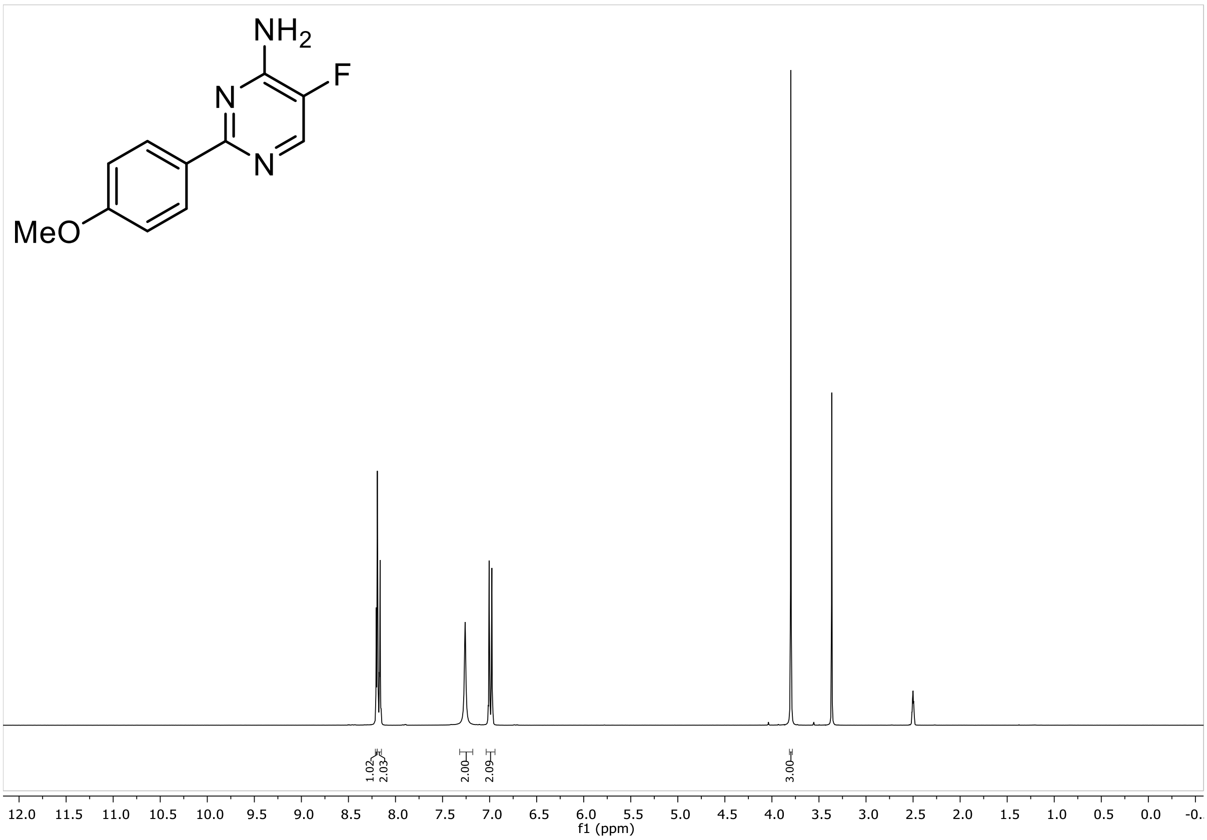

Figure S34: ^{1}H-NMR (300 MHz, DMSO-d6): 5-Fluoro-2-(4-methoxyphenyl)pyrimidine-4-amine (**10k**).

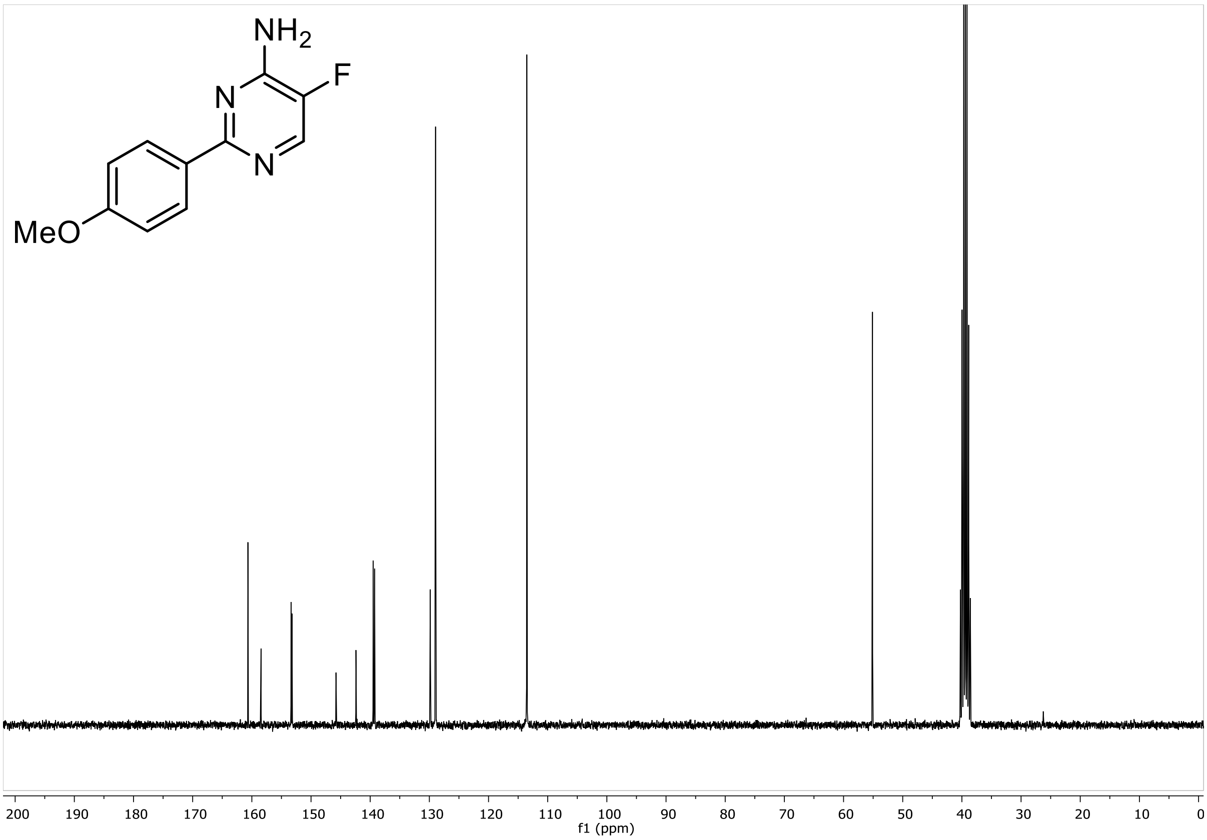

Figure S35: ^{13}C-NMR (75 MHz, DMSO-d_6): 5-Fluoro-2-(4-methoxyphenyl)pyrimidine-4-amine (**10k**).

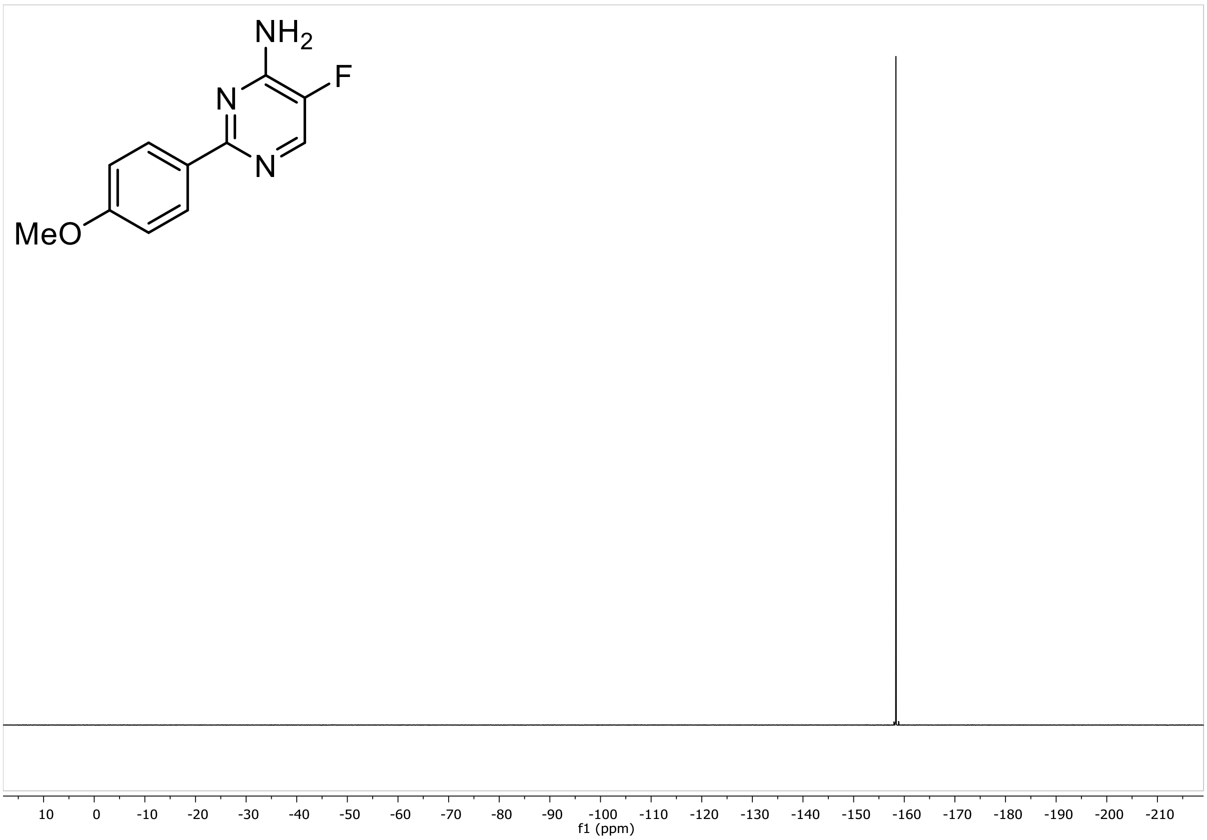

Figure S36: ^{19}F-NMR (282 MHz, DMSO-d_6): 5-Fluoro-2-(4-methoxyphenyl)pyrimidine-4-amine (**10k**).

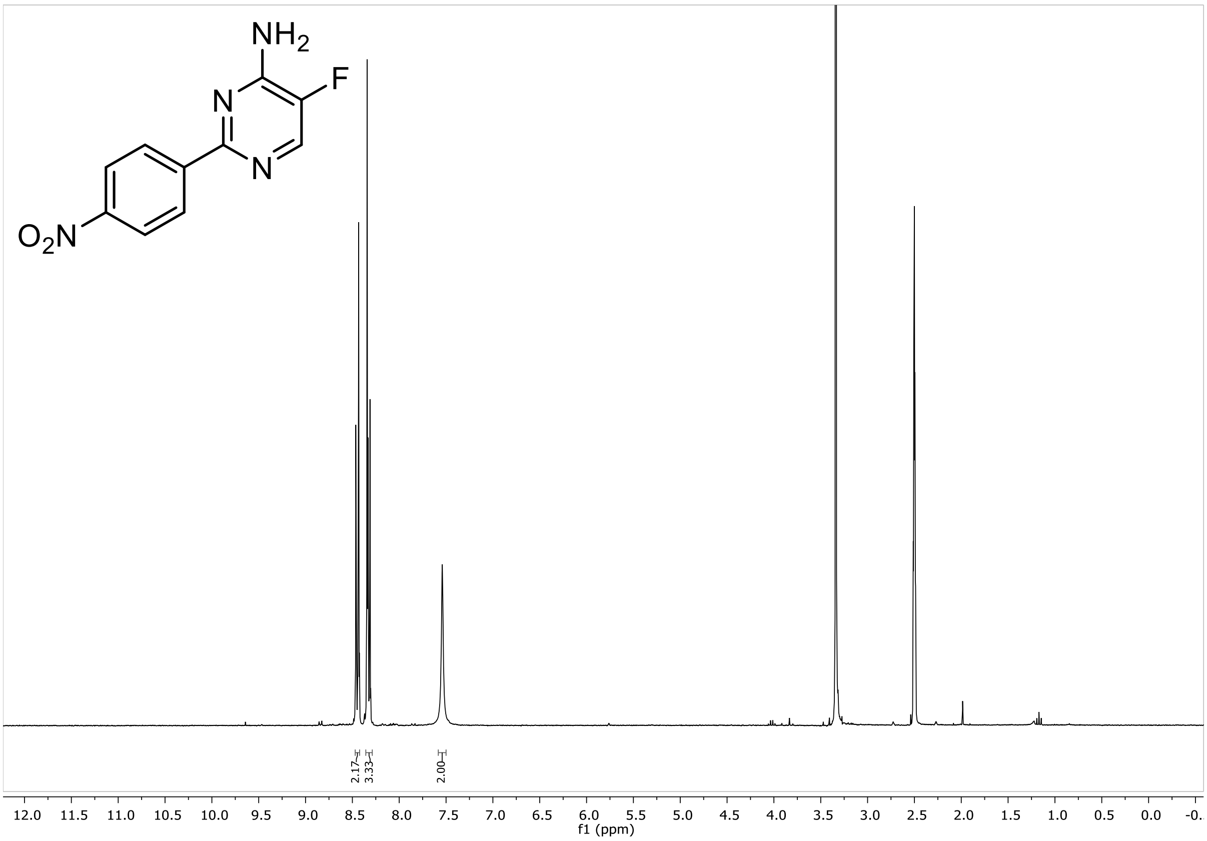

Figure S37: ^{1}H-NMR (300 MHz, DMSO-d$_6$): 5-Fluoro-2-(4-nitrophenyl)pyrimidine-4-amine (**10l**).

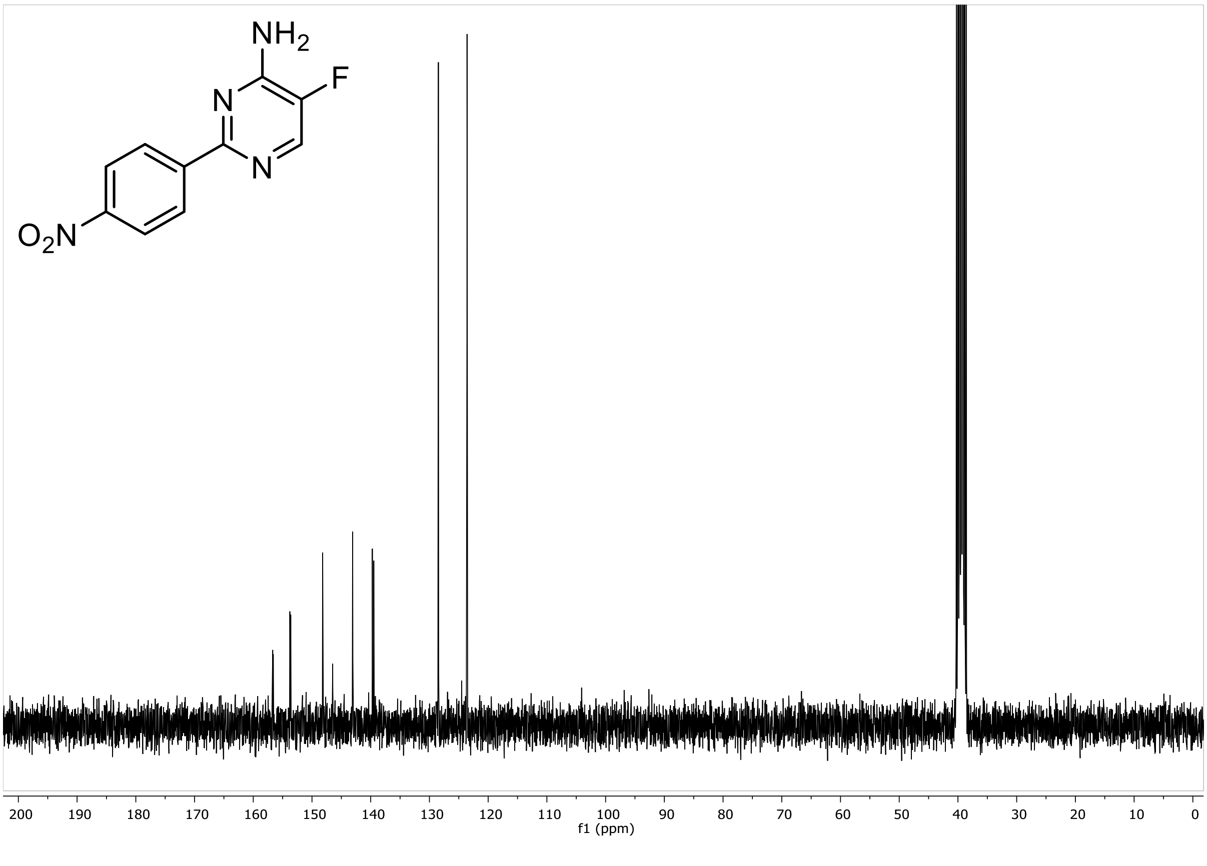

Figure S38: ^{13}C-NMR (75 MHz, DMSO-d$_6$): 5-Fluoro-2-(4-nitrophenyl)pyrimidine-4-amine (**10l**).

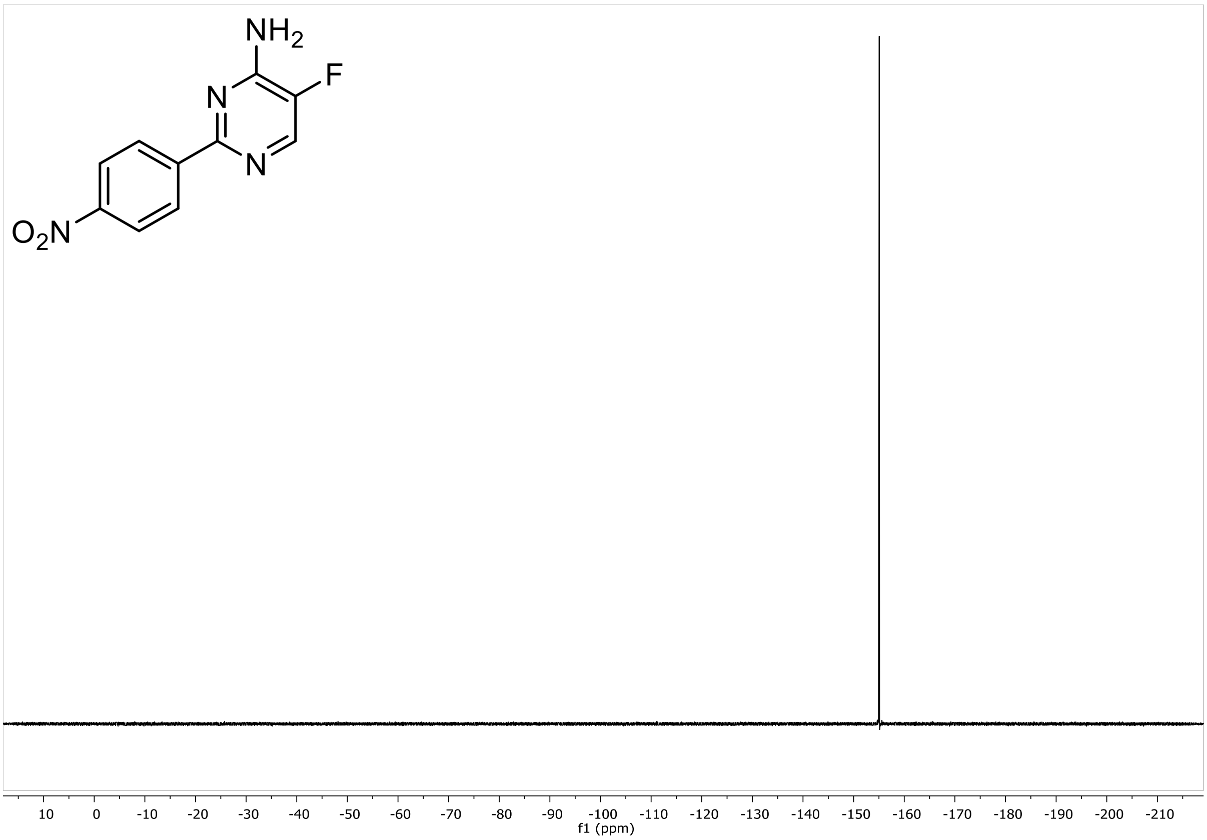

Figure S39: ^{19}F-NMR (282 MHz, DMSO-d$_6$): 5-Fluoro-2-(4-nitrophenyl)pyrimidine-4-amine (**10l**).

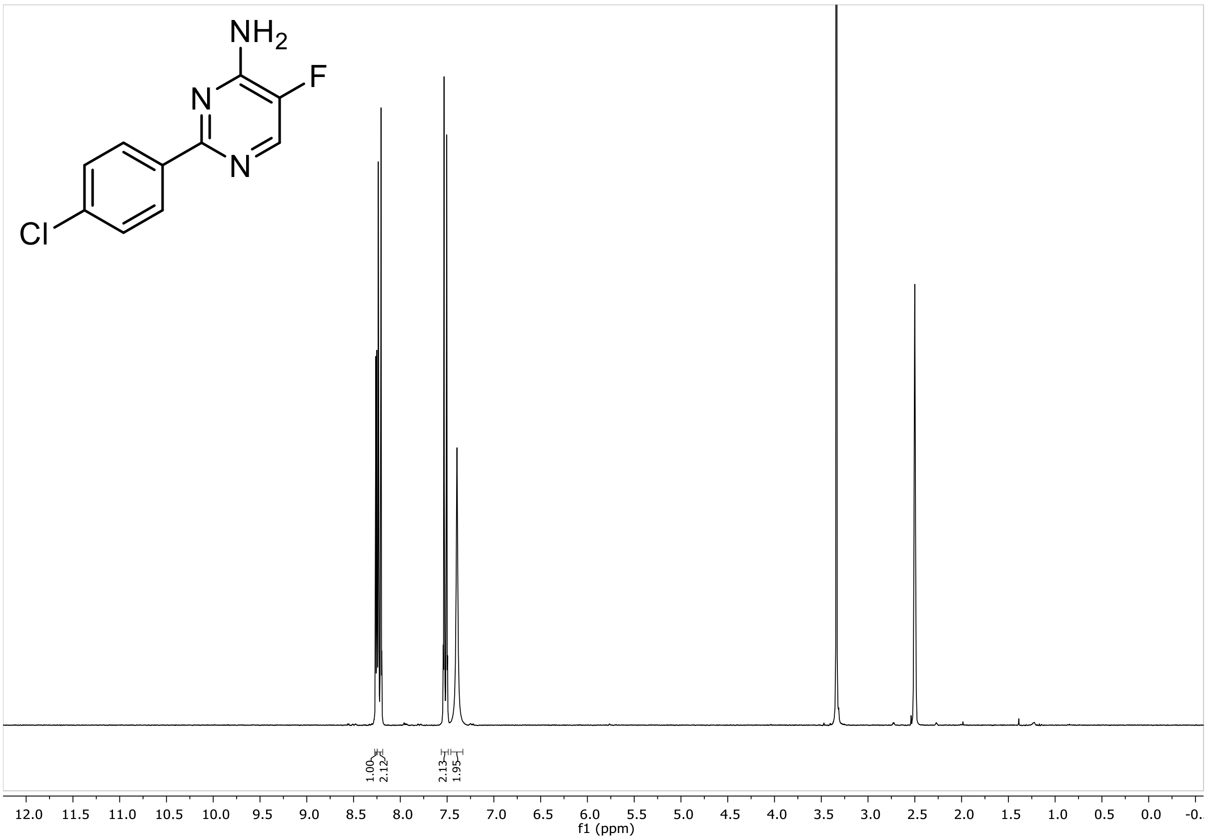

Figure S40: ^{1}H-NMR (300 MHz, DMSO-d$_6$): 2-(4-Chlorophenyl)-5-fluoropyrimidine-4-amine (**10m**).

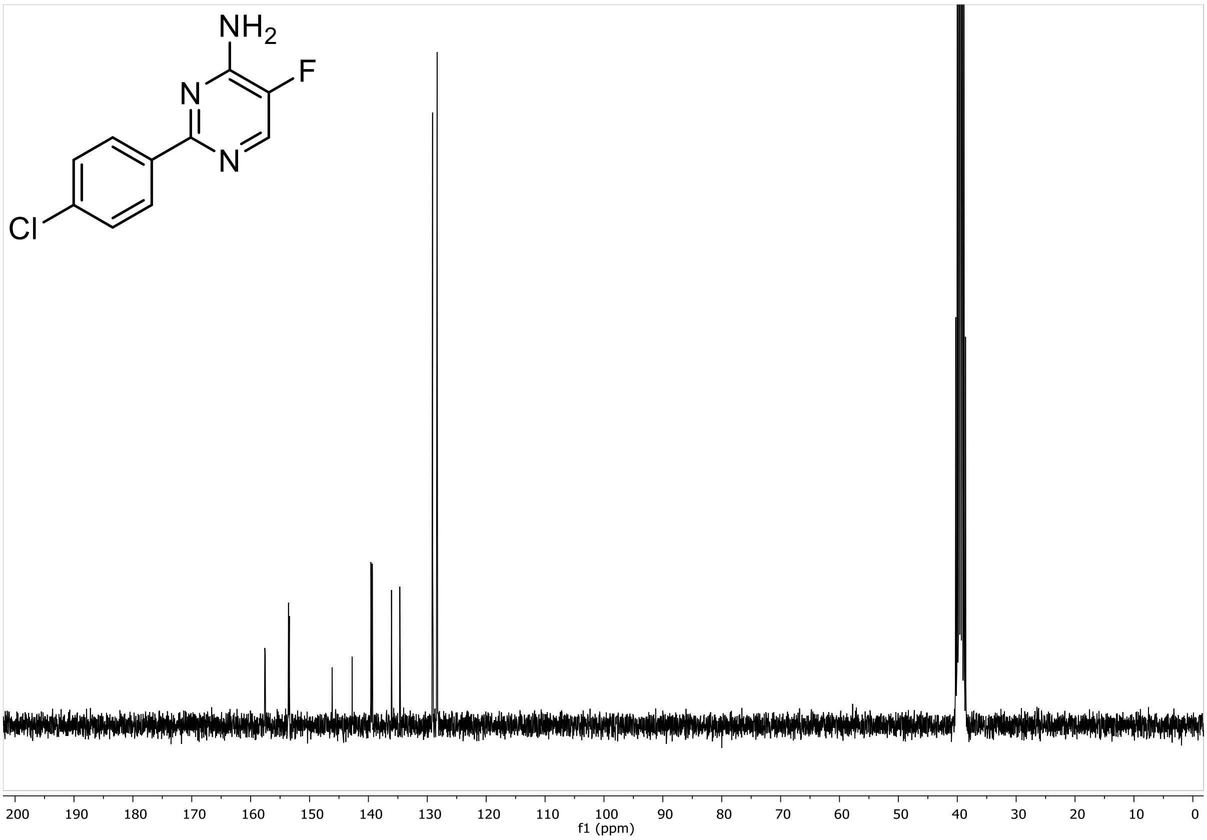

Figure S41: ^{13}C-NMR (75 MHz, DMSO-d$_6$): 2-(4-Chlorophenyl)-5-fluoropyrimidine-4-amine (**10m**).

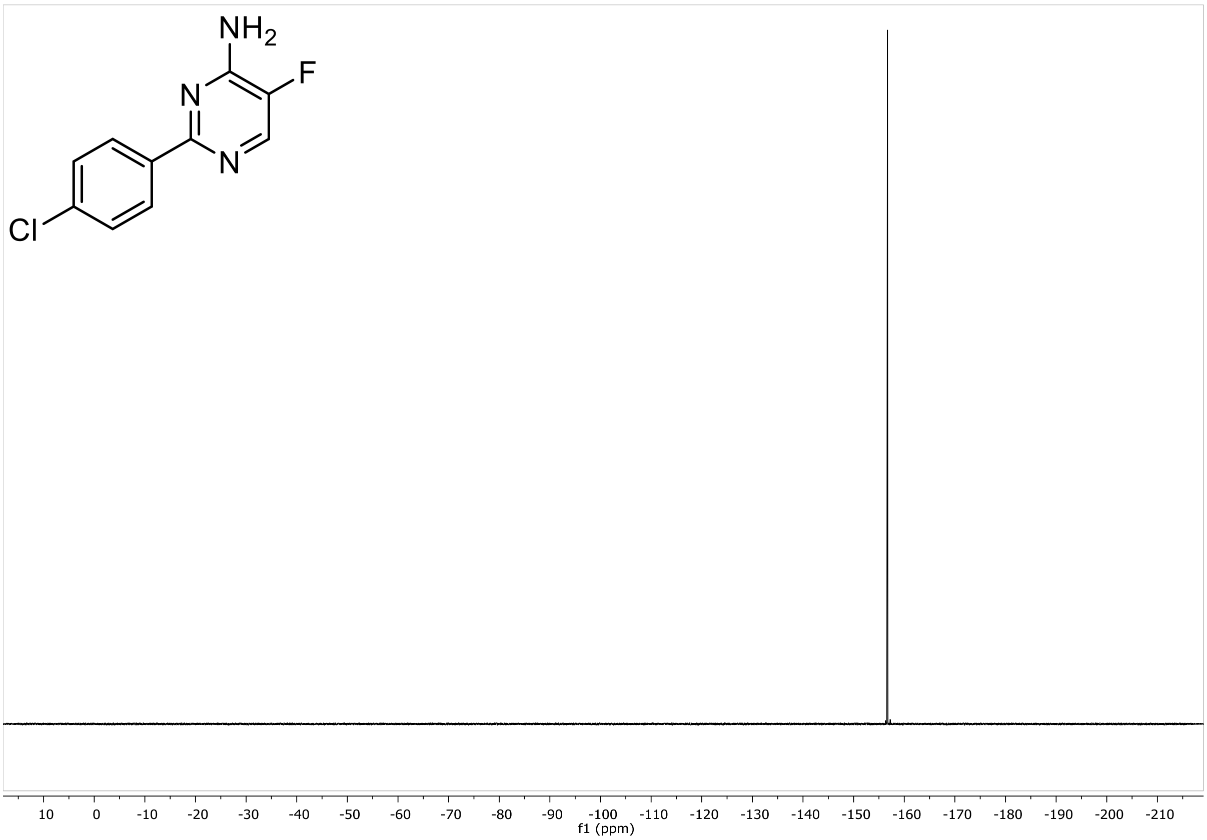

Figure S42: ^{19}F-NMR (282 MHz, DMSO-d$_6$): 2-(4-Chlorophenyl)-5-fluoropyrimidine-4-amine (**10m**).

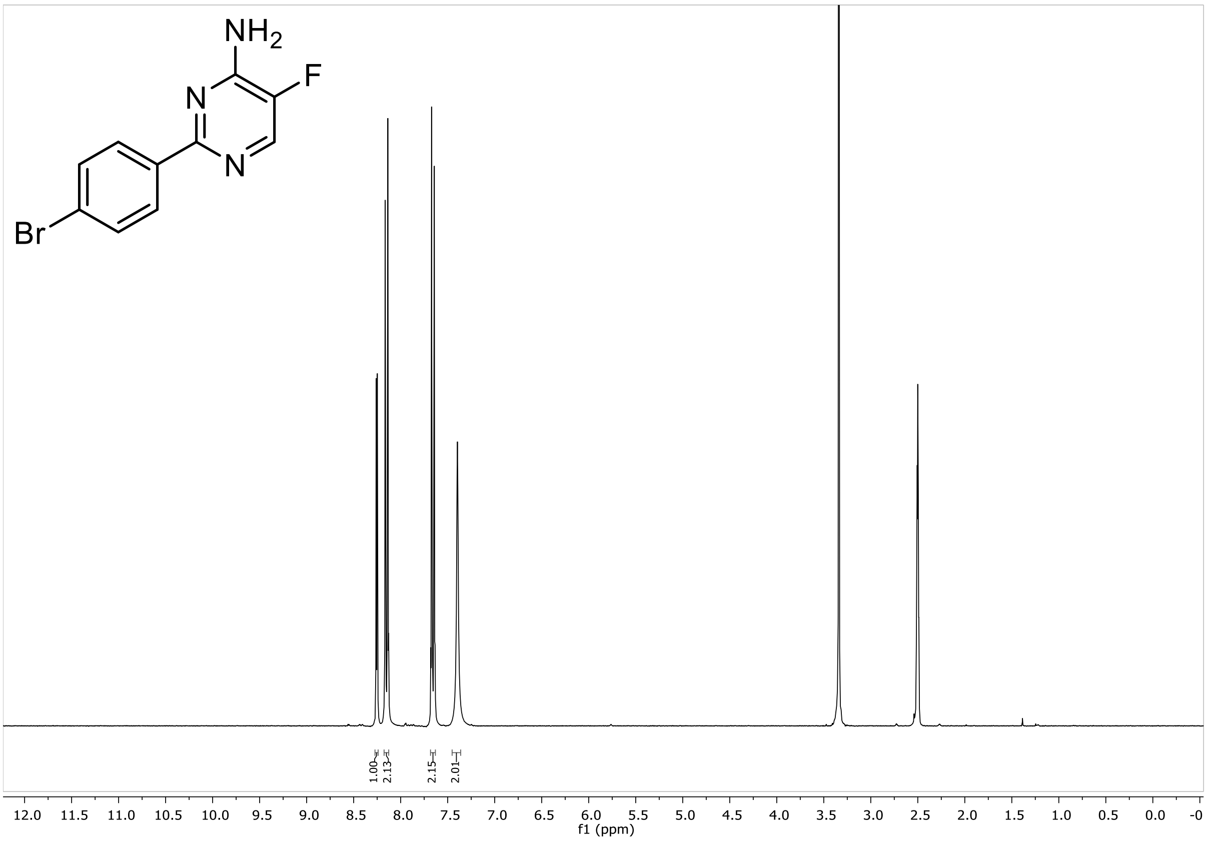

Figure S43: ^{1}H-NMR (300 MHz, DMSO-d_6): 2-(4-Bromophenyl)-5-fluoropyrimidine-4-amine (**10n**).

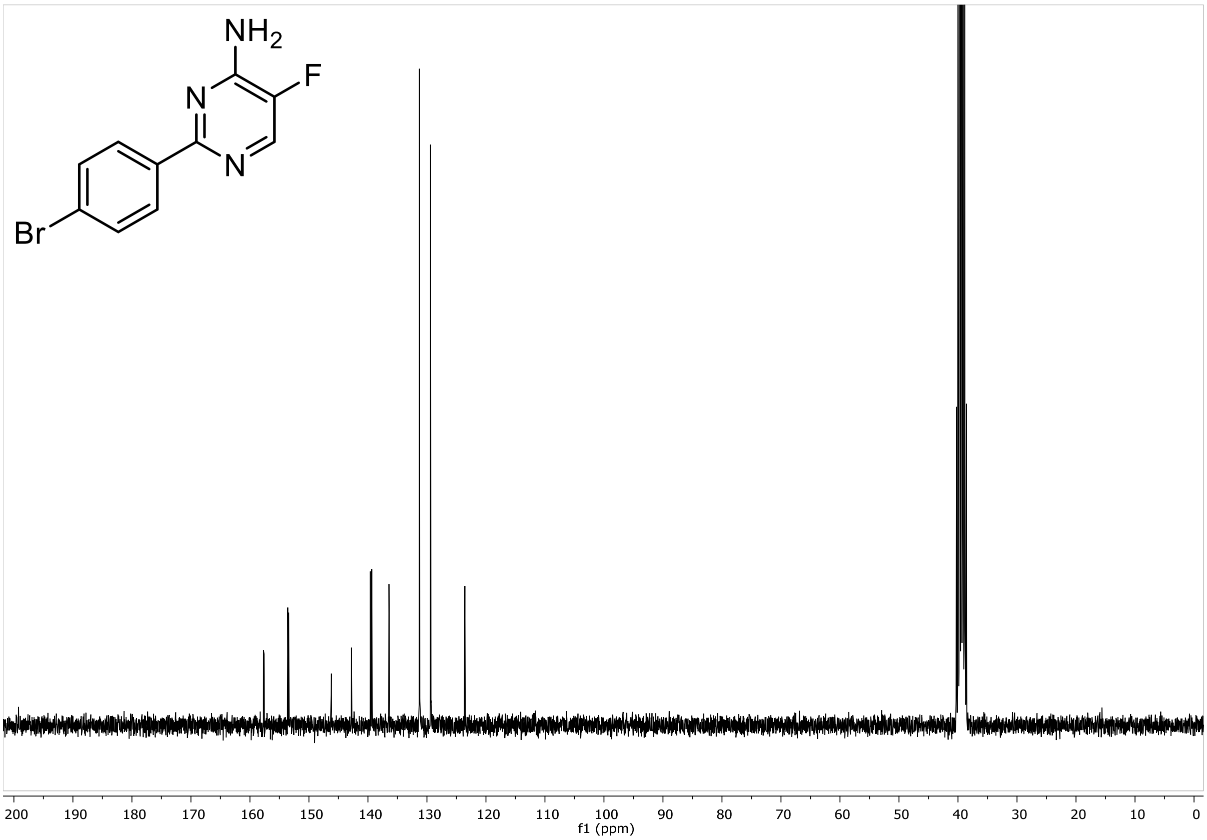

Figure S44: ^{13}C-NMR (75 MHz, DMSO-d_6): 2-(4-Bromophenyl)-5-fluoropyrimidine-4-amine (**10n**).

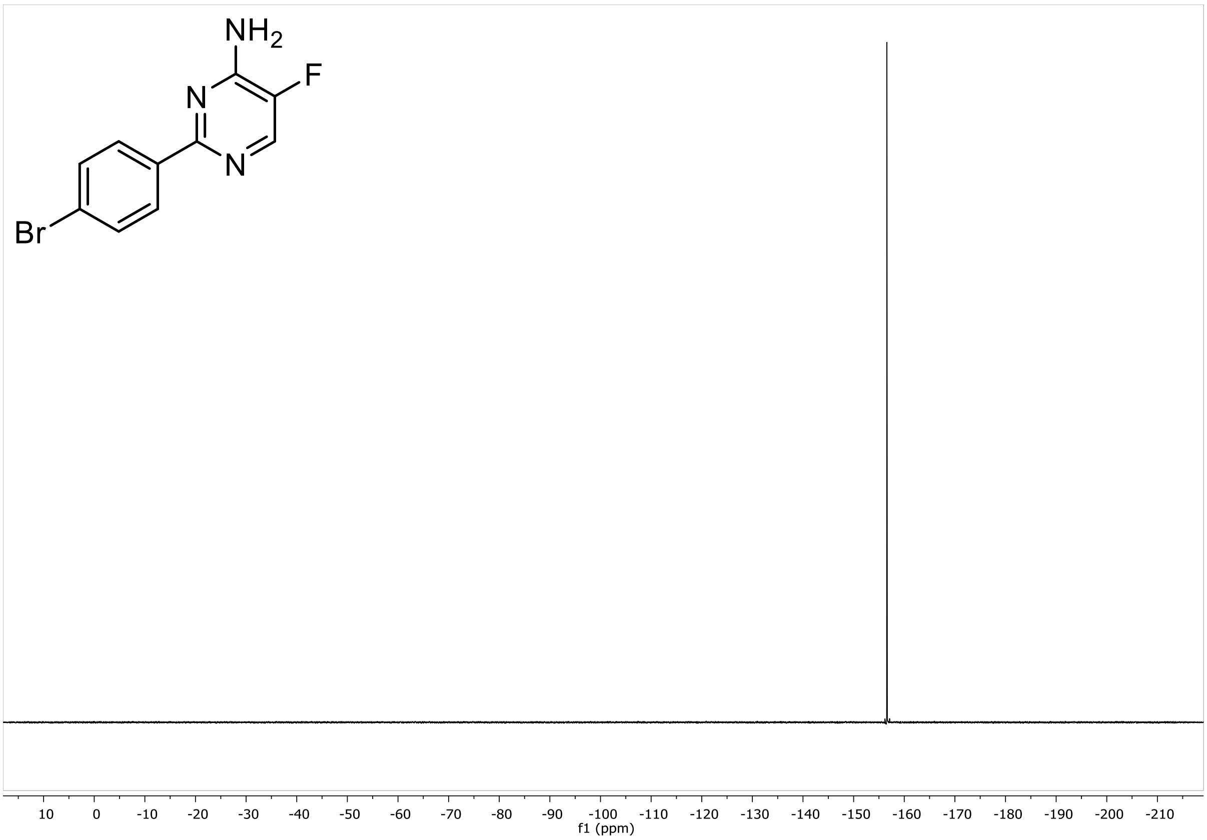

Figure S45: ^{19}F-NMR (282 MHz, DMSO-d6): 2-(4-Bromophenyl)-5-fluoropyrimidine-4-amine (**10n**).

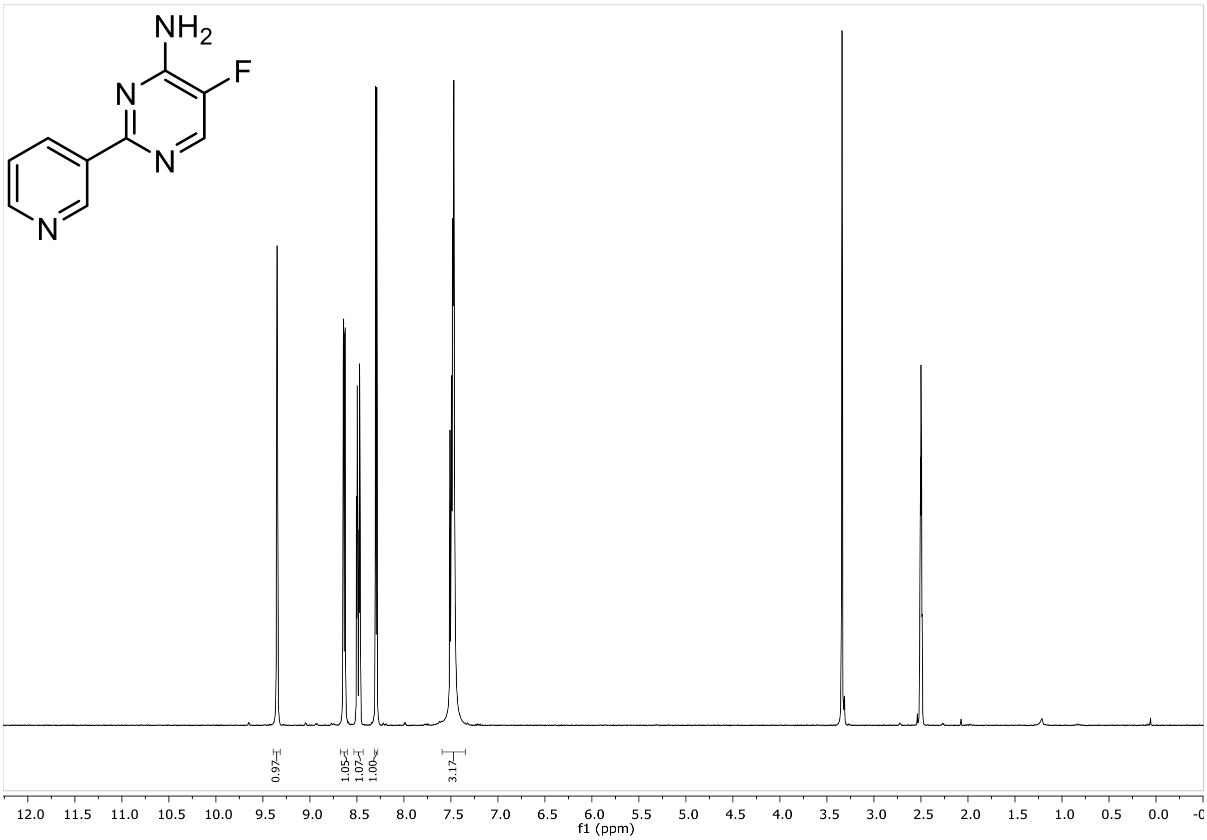

Figure S46: ^{1}H-NMR (300 MHz, DMSO-d6): 5-Fluoro-2-(pyridine-3-yl)pyrimidine-4-amine (**10o**).

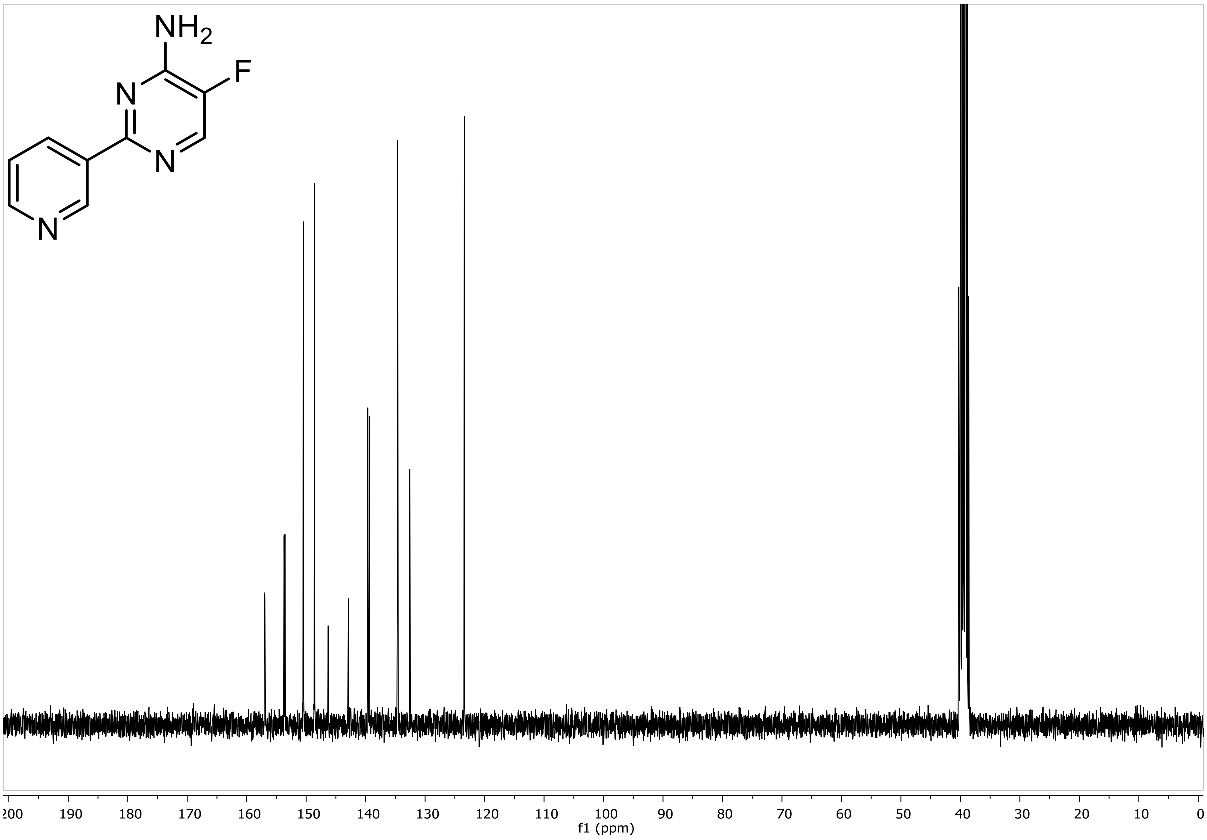

Figure S47: ^{13}C-NMR (75 MHz, DMSO-d$_6$): 5-Fluoro-2-(pyridine-3-yl)pyrimidine-4-amine (**10o**).

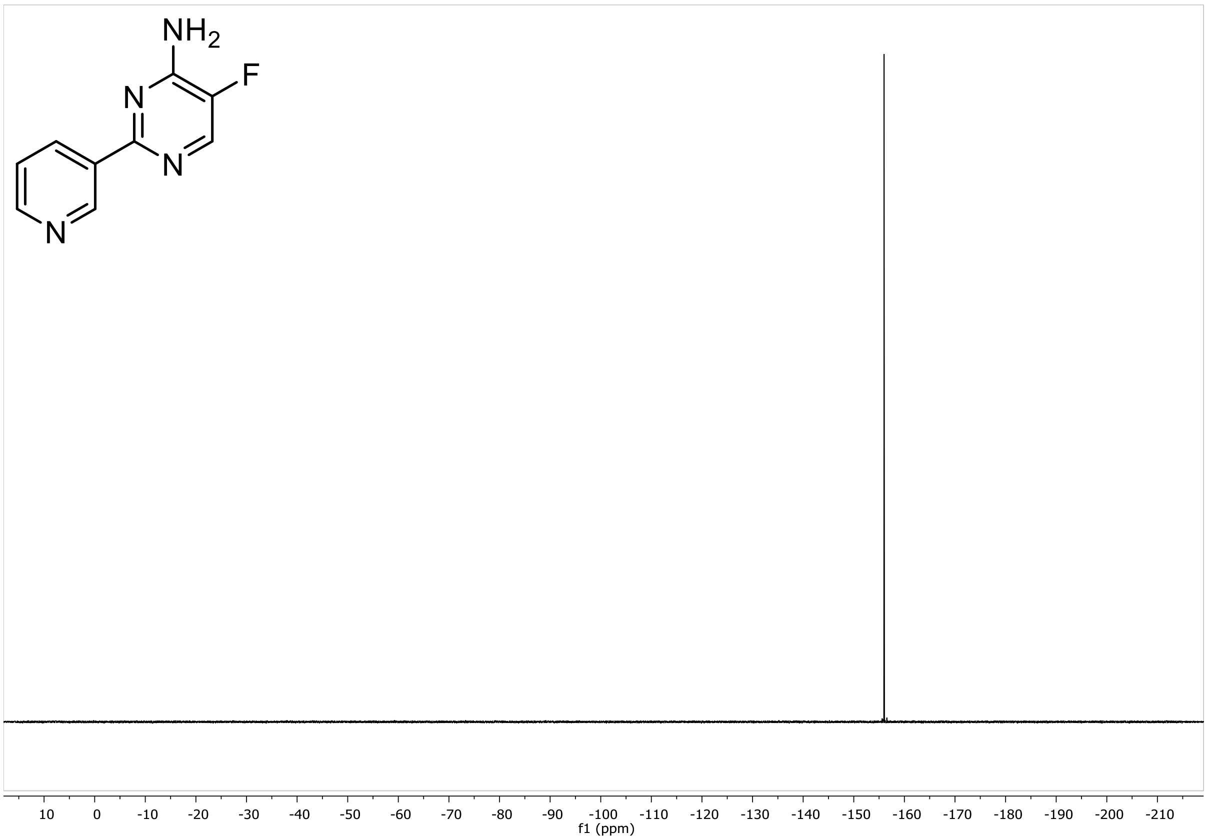

Figure S48: ^{19}F-NMR (282 MHz, DMSO-d$_6$): 5-Fluoro-2-(pyridine-3-yl)pyrimidine-4-amine (**10o**).

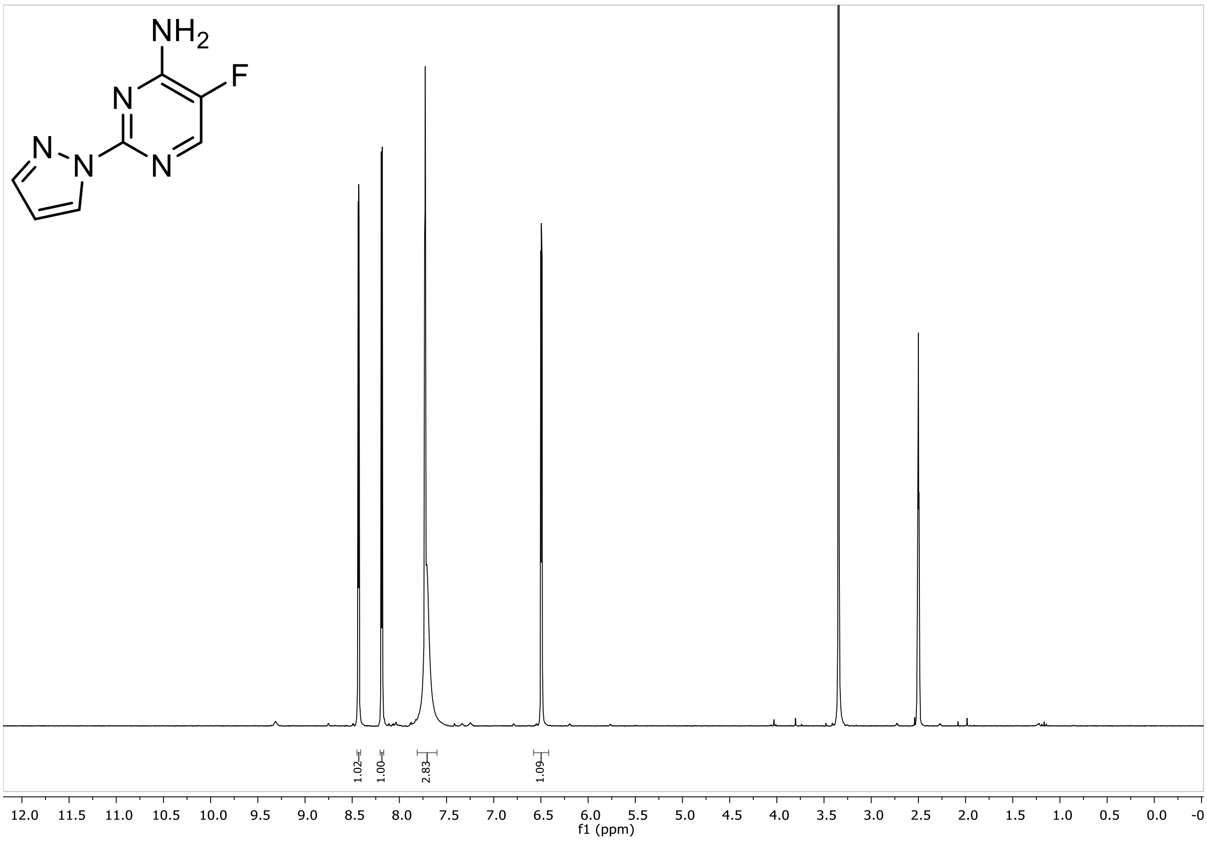

Figure S49: ^{1}H-NMR (300 MHz, DMSO-d6): 5-Fluoro-2-(1*H*-pyrazolo-1-yl)pyrimidine-4-amine (**10p**).

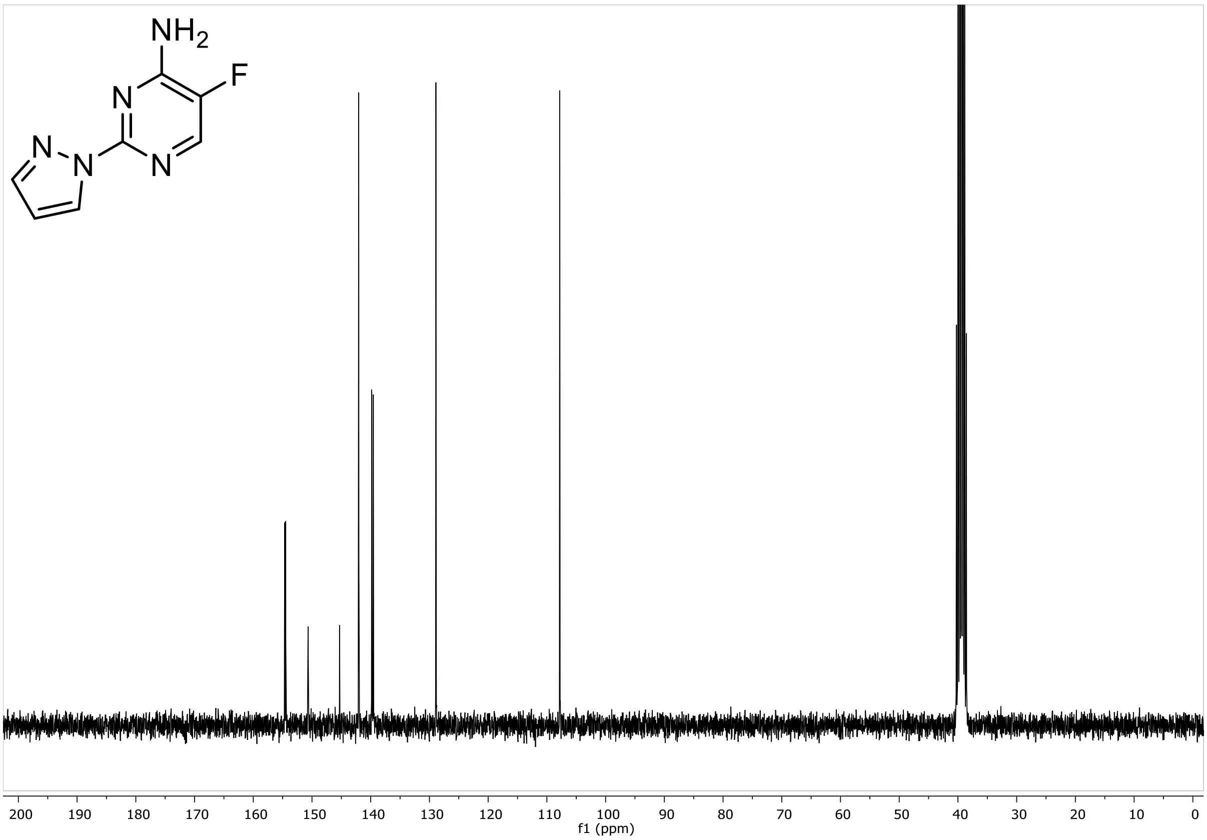

Figure S50: ^{13}C-NMR (75 MHz, DMSO-d6): 5-Fluoro-2-(1*H*-pyrazolo-1-yl)pyrimidine-4-amine (**10p**).

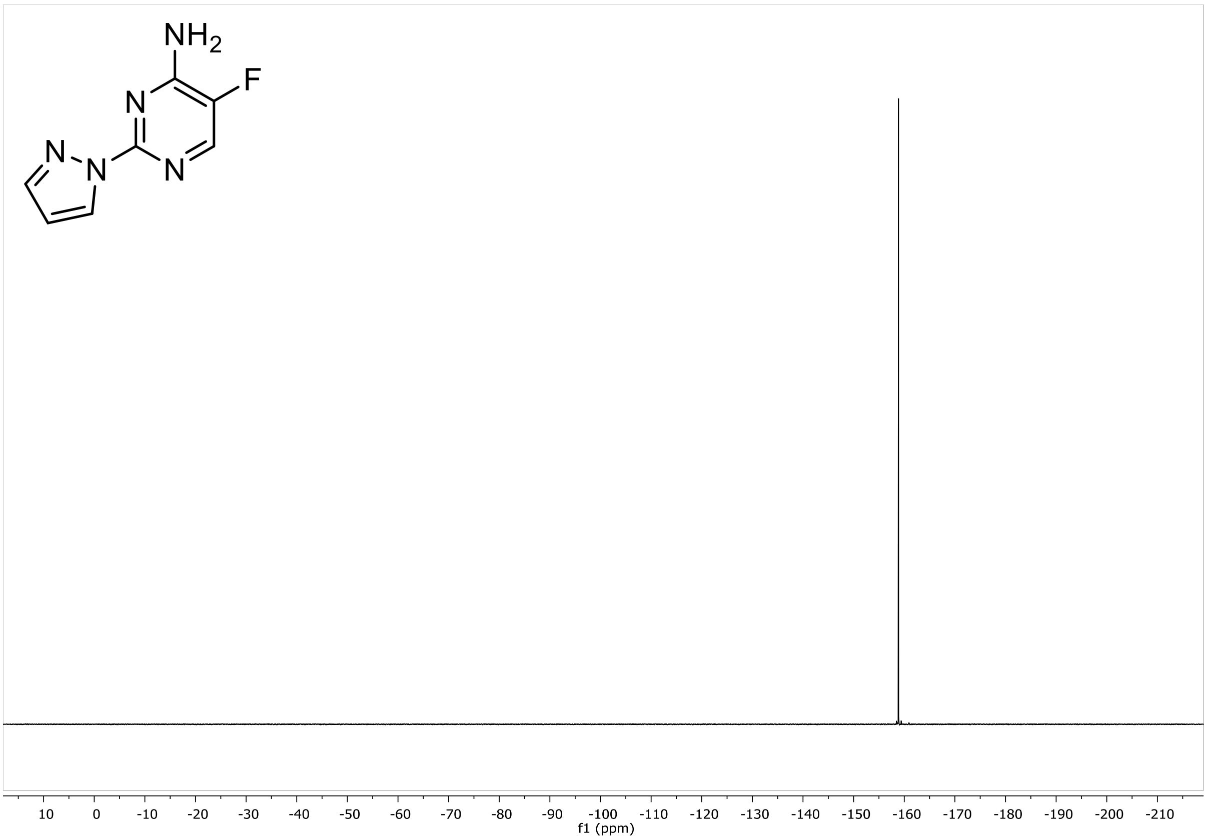

Figure S51: ^{19}F-NMR (282 MHz, DMSO-d$_6$): 5-Fluoro-2-(1*H*-pyrazolo-1-yl)pyrimidine-4-amine (**10p**).

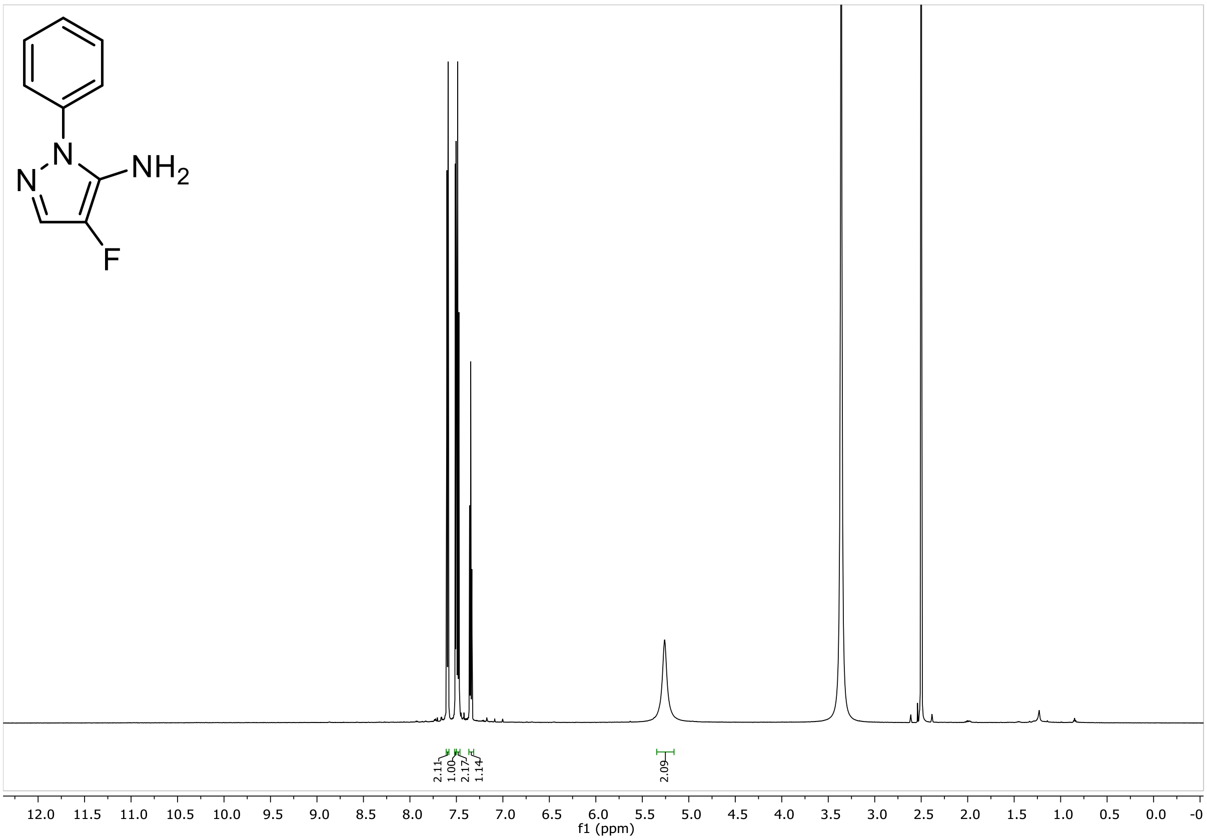

Figure S52: ^{1}H-NMR (600 MHz, DMSO-d$_6$): 4-Fluoro-1-phenyl-1*H*-pyrazole-5-amine (**13a**).

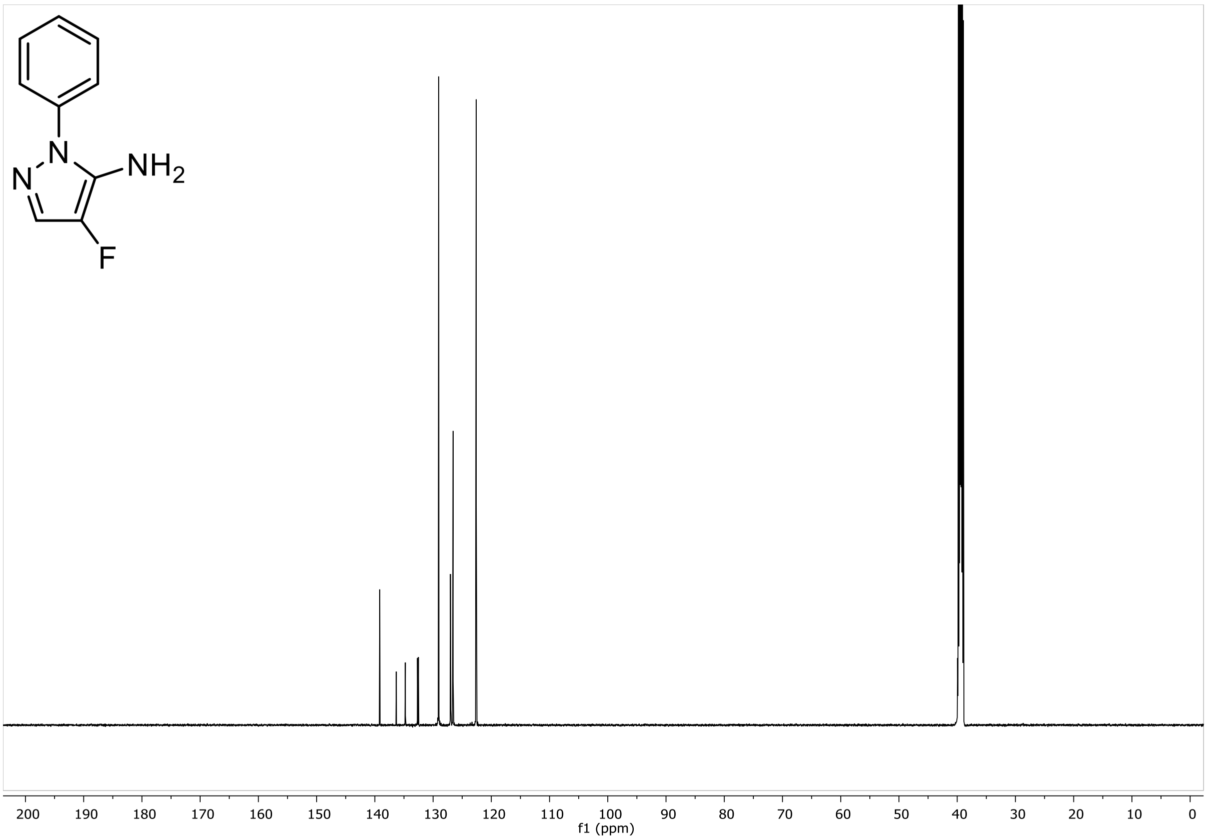

Figure S53: ^{13}C-NMR (151 MHz, DMSO-d$_6$): 4-Fluoro-1-phenyl-1*H*-pyrazole-5-amine (**13a**).

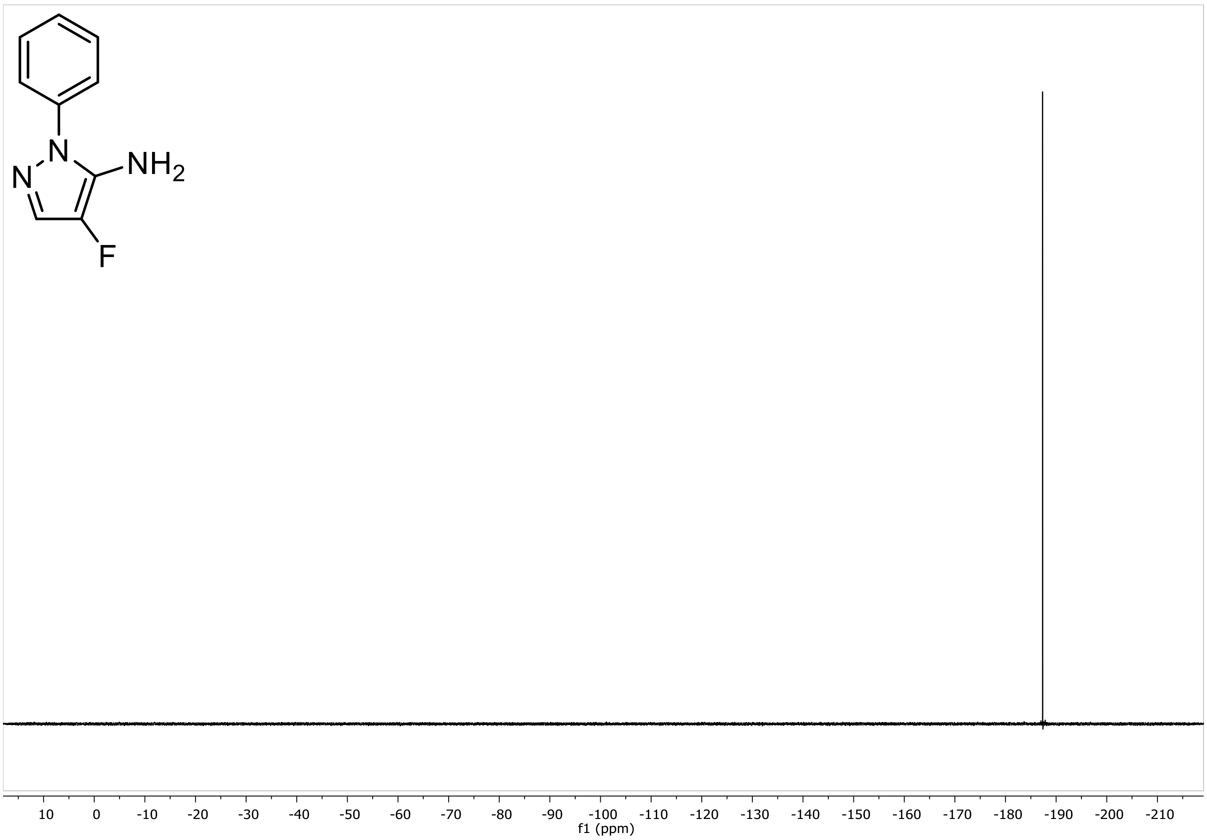

Figure S54: ^{19}F-NMR (282 MHz, DMSO-d$_6$): 4-Fluoro-1-phenyl-1*H*-pyrazole-5-amine (**13a**).

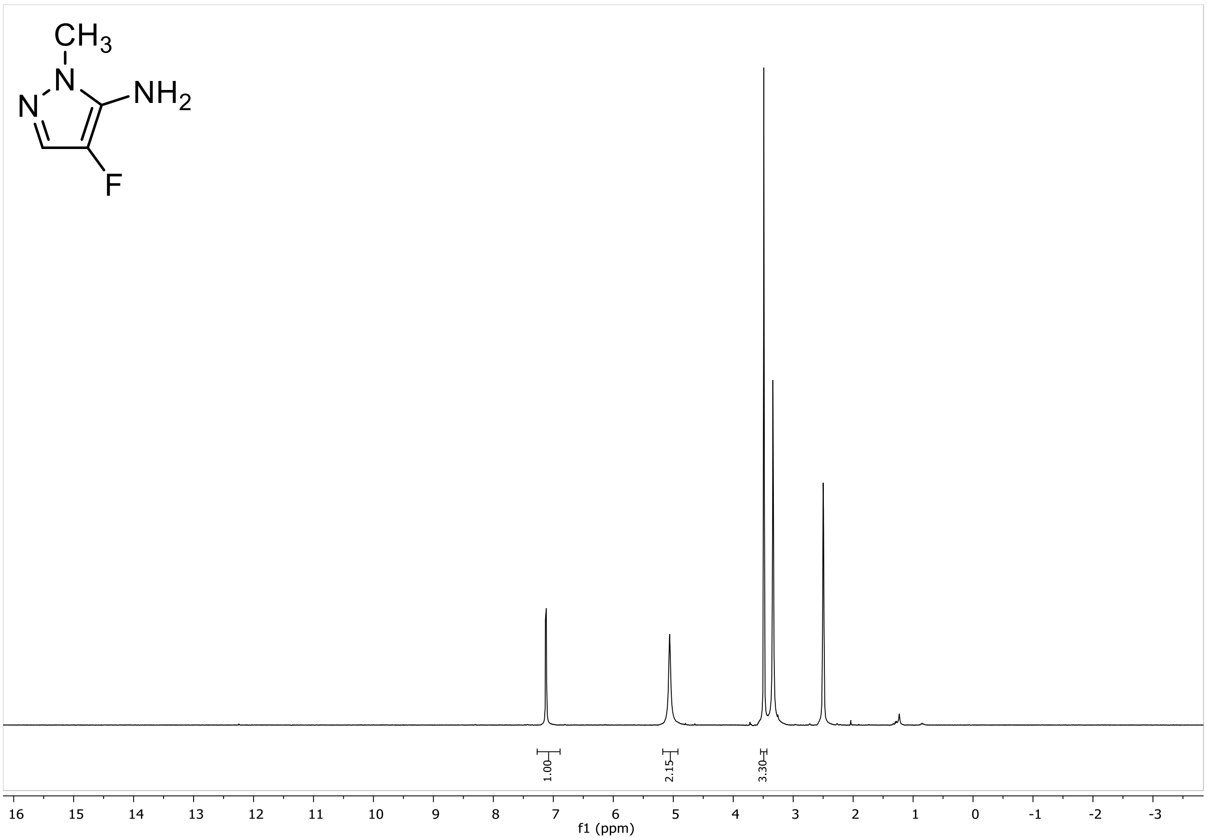

Figure S55: ^{1}H-NMR (300 MHz, DMSO-d6): 4-Fluoro-1-methyl-1*H*-pyrazole-5-amine (**13d**).

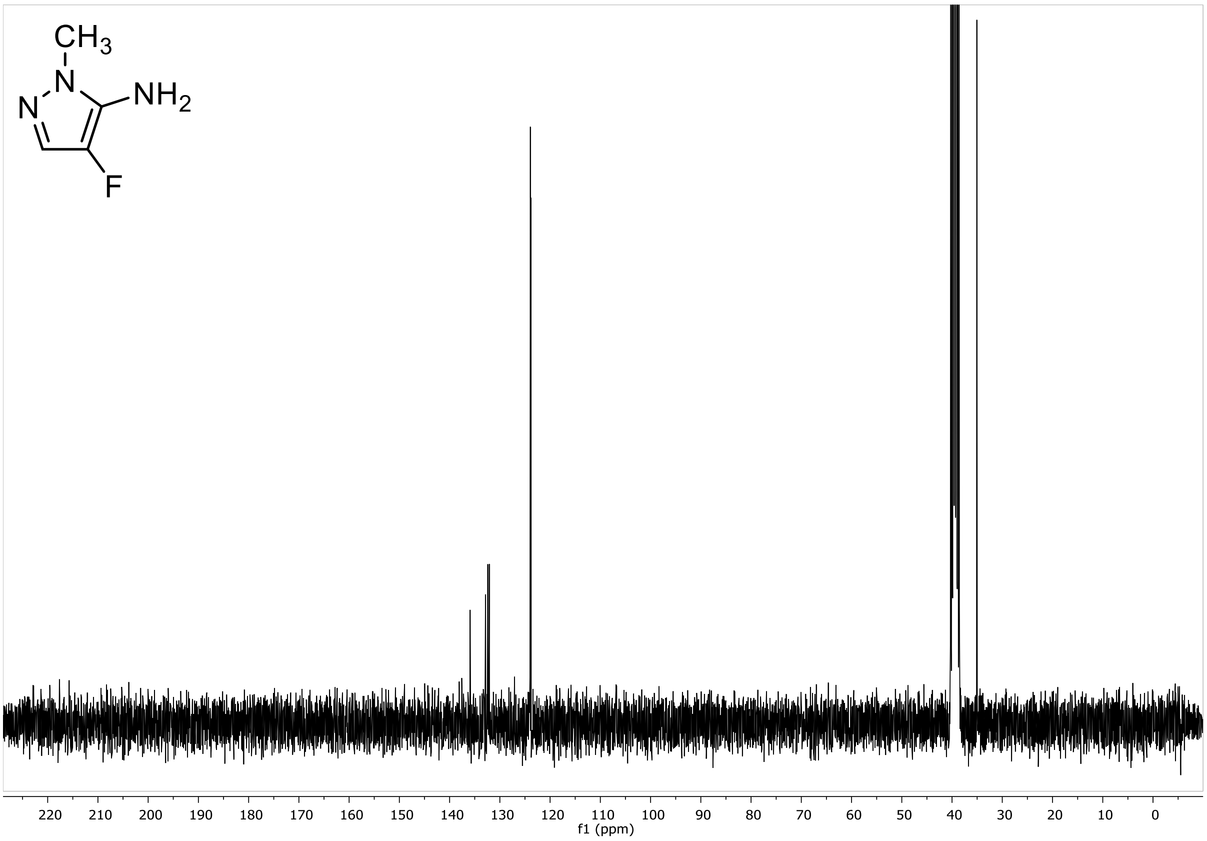

Figure S56: ^{13}C-NMR (75 MHz, DMSO-d6): 4-Fluoro-1-methyl-1*H*-pyrazole-5-amine (**13d**).

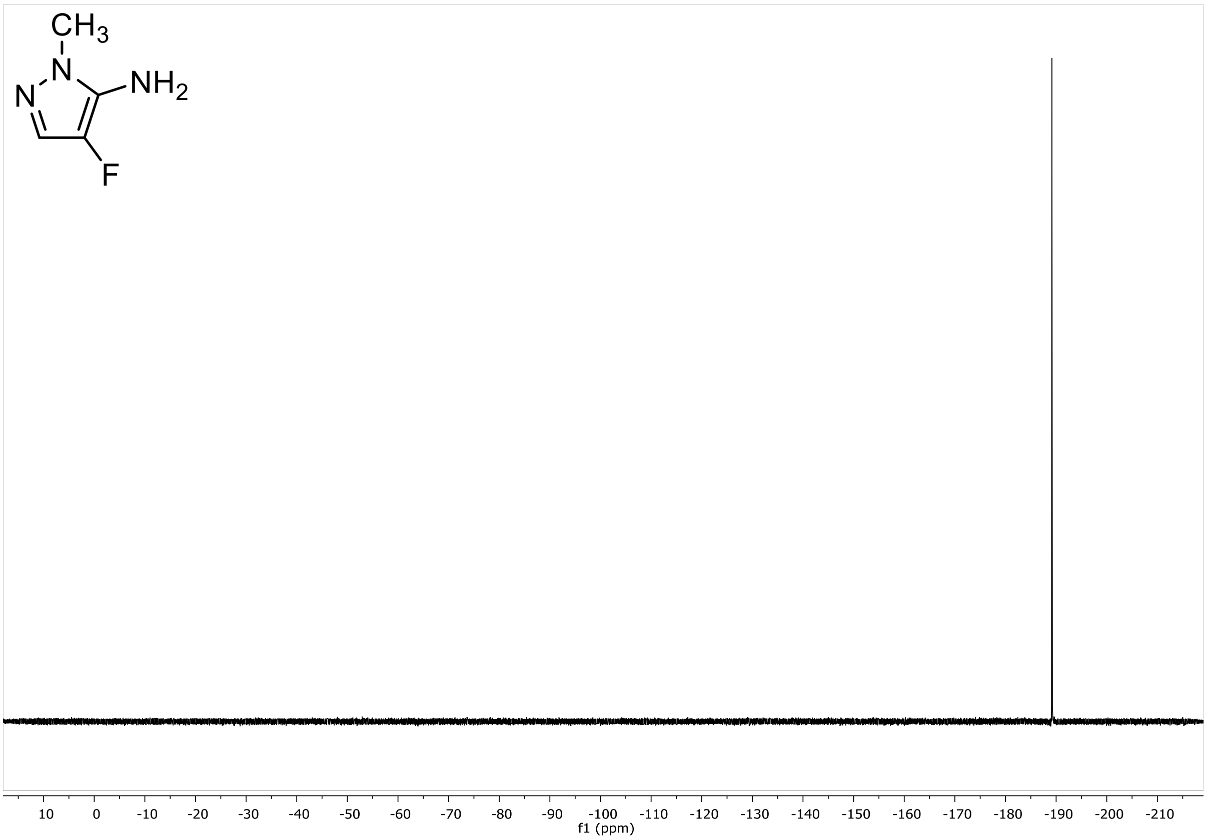

Figure S57: ^{19}F-NMR (282 MHz, DMSO-d_6): 4-Fluoro-1-methyl-1*H*-pyrazole-5-amine (**13d**).

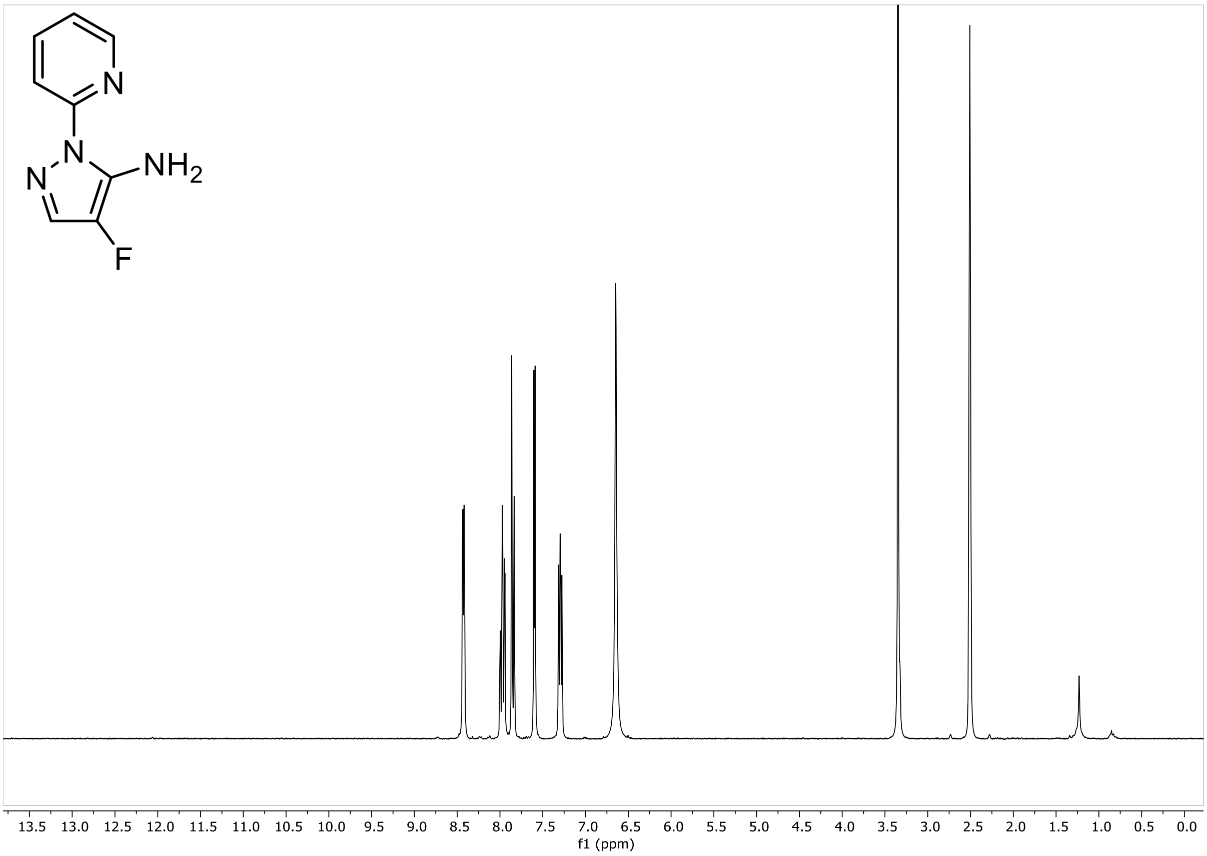

Figure S58: ^{1}H-NMR (300 MHz, DMSO-d_6): 4-Fluoro-1-(pyridine-2-yl)-1*H*-pyrazole-5-amine (**13e**).

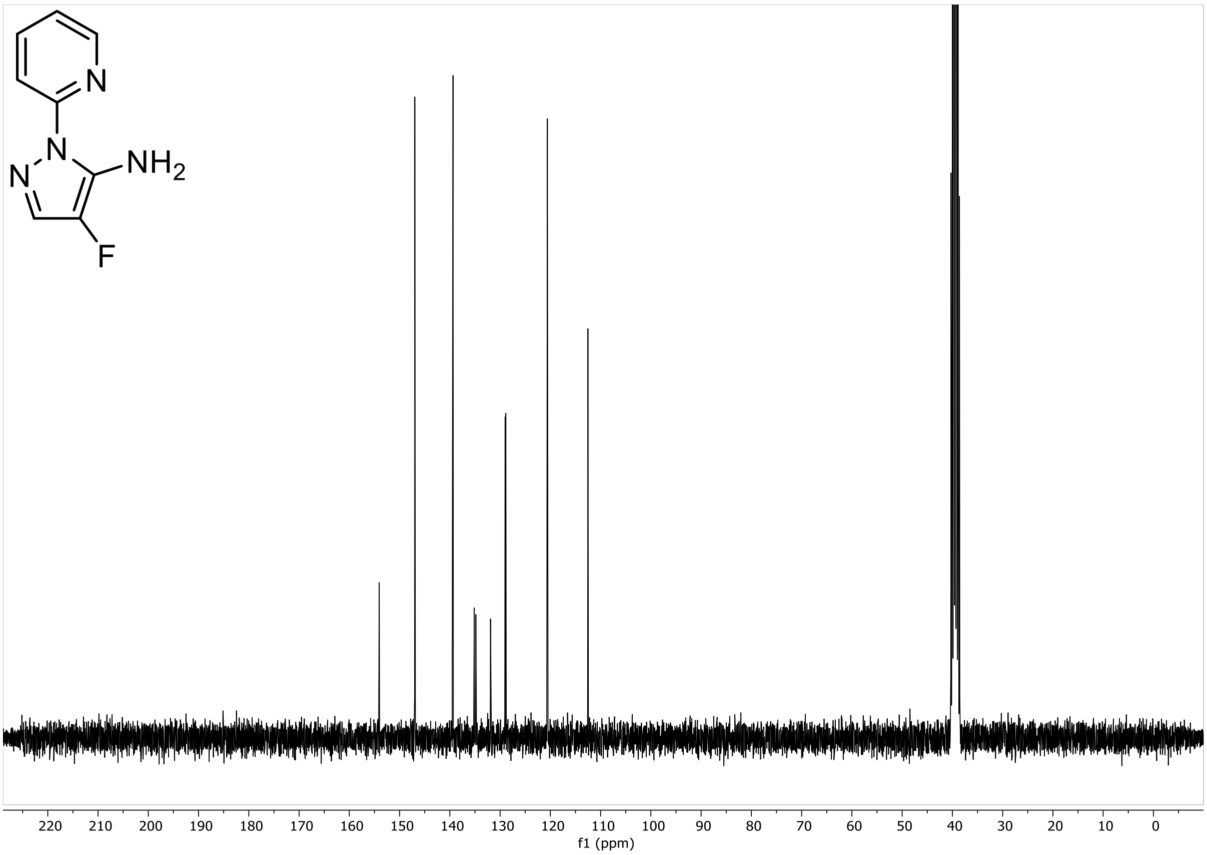

Figure S59: ^{13}C-NMR (75 MHz, DMSO-d$_6$): 4-Fluoro-1-(pyridine-2-yl)-1*H*-pyrazole-5-amine (**13e**).

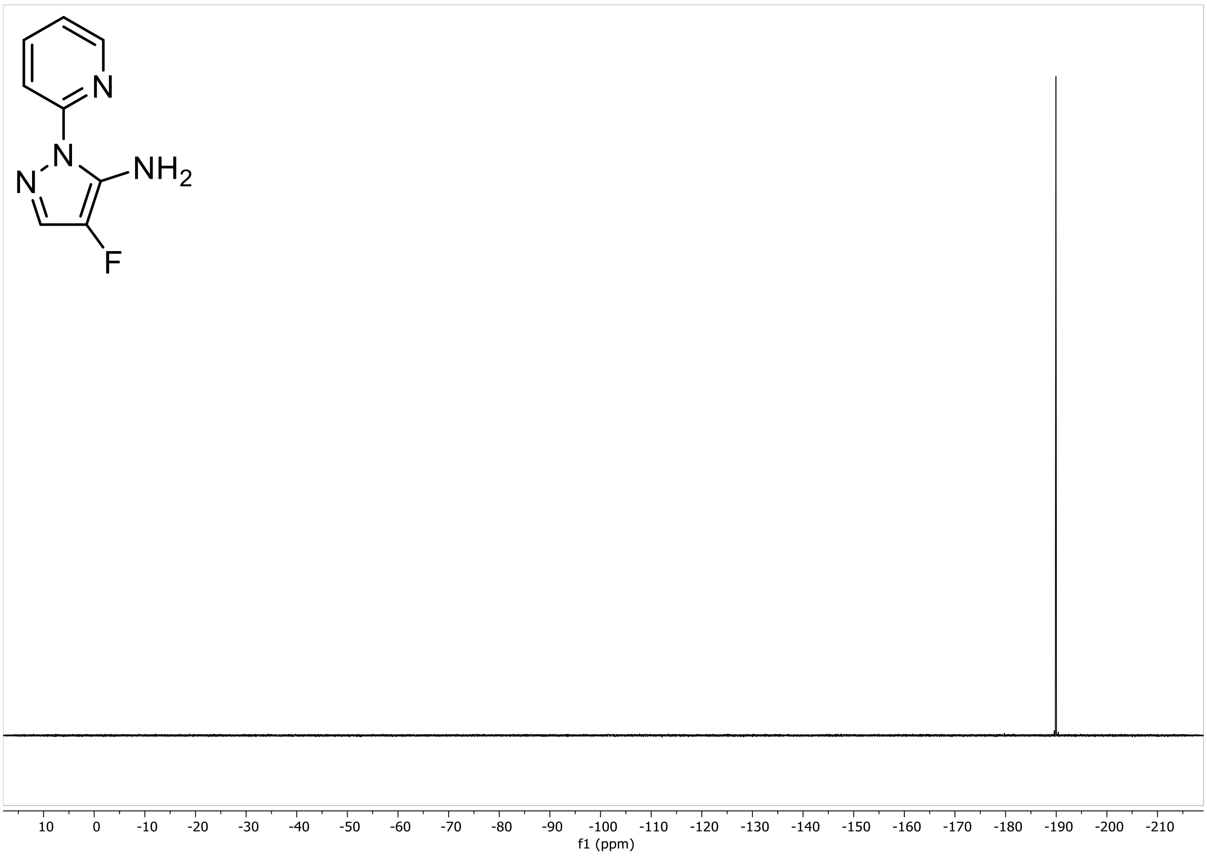

Figure S60: ^{19}F-NMR (282 MHz, DMSO-d$_6$): 4-Fluoro-1-(pyridine-2-yl)-1*H*-pyrazole-5-amine (**13e**).
